# Safety and Efficacy of Repeated Low-Dose LSD for ADHD Treatment in Adults: A Randomized Clinical Trial

**DOI:** 10.1001/jamapsychiatry.2025.0044

**Published:** 2025-03-19

**Authors:** Lorenz Mueller, Joyce Santos de Jesus, Yasmin Schmid, Felix Müller, Anna Becker, Aaron Klaiber, Isabelle Straumann, Dino Luethi, Eline C. H. M. Haijen, Petra P. M. Hurks, Kim P. C. Kuypers, Matthias E. Liechti

**Affiliations:** 1 Division of Clinical Pharmacology and Toxicology, University Hospital Basel, Basel, Switzerland; 2 Department of Clinical Research, University of Basel, Basel, Switzerland; 3 University Psychiatric Clinic Basel, Basel, Switzerland; 4 Department of Biomedicine, University of Basel, Basel, Switzerland; 5 Department of Neuropsychology and Psychopharmacology, Faculty of Psychology and Neuroscience, Maastricht University, Maastricht, the Netherlands

## Abstract

**Importance:**

Microdosing psychedelics, including lysergic acid diethylamide (LSD), has gained attention for its potential benefits in several psychiatric disorders, including attention-deficit/hyperactivity disorder (ADHD). However, LSD’s efficacy in reducing ADHD symptoms remains unknown.

**Objective:**

To determine the safety and efficacy of repeated low doses of LSD in reducing ADHD symptoms compared with placebo.

**Design, Setting, and Participants:**

This was a 6-week, multicenter, double-blind, placebo-controlled, parallel-group phase 2A randomized clinical trial conducted between December 17, 2021, and December 4, 2023. Data were analyzed from March 22, 2024, to August 19, 2024. Outpatient treatment was provided at 2 centers: University Hospital in Basel, Switzerland, and Maastricht University in the Netherlands. Adults aged 18 to 65 years with a prior ADHD diagnosis who presented with moderate to severe symptoms (Adult Investigator Symptom Rating Scale [AISRS] score ≥26 and Clinical Global Impression Severity score ≥4) were eligible for inclusion. Key exclusion criteria included selected current major psychiatric or somatic disorders and the use of potentially interacting medications.

**Intervention:**

Participants received either LSD (20 μg) or placebo twice weekly for 6 weeks (total of 12 doses).

**Main Outcome and Measures:**

The primary outcome was the change in ADHD symptoms from baseline to week 6, assessed by the AISRS and analyzed with a mixed-effects model for repeated measures.

**Results:**

A total of 53 participants were randomized to LSD (n = 27) or placebo (n = 26). Mean (SD) participant age was 37 (12) years, and 22 participants (42%) were female. The LSD group presented a mean AISRS improvement of −7.1 points (95% CI, −10.1 to −4.0). The placebo group presented a mean AISRS improvement of −8.9 points (95% CI, −12.0 to −5.8), with no difference between groups. LSD was physically safe and psychologically well tolerated overall.

**Conclusions and Relevance:**

In this randomized clinical trial, repeated low-dose LSD administration was safe in an outpatient setting, but it was not more efficacious than placebo in reducing ADHD symptoms.

**Trial Registration:**

ClinicalTrials.gov Identifier: NCT05200936

## Key Points

**Question:**

Does twice-weekly low-dose (20 μg) lysergic acid diethylamide (LSD) over 6 weeks reduce symptoms of attention-deficit/hyperactivity disorder (ADHD) in adults with moderate to severe ADHD compared with placebo?

**Findings:**

In this multicenter, double-blind, placebo-controlled randomized clinical trial in 53 individuals, both the LSD and placebo groups exhibited a significant reduction of ADHD symptoms. However, there was no difference in symptom reduction between the 2 groups.

**Meaning:**

LSD was not efficacious in reducing ADHD symptoms compared with placebo; these results question the anecdotal practice and highlight the importance of placebo-controlled trials in low-dose psychedelic research.

## Teaser

This double-blind, placebo-controlled randomized clinical trial determines the safety and efficacy of repeated low doses of lysergic acid diethylamide (LSD) in reducing attention-deficit/hyperactivity disorder (ADHD) symptoms compared with placebo.

## Introduction

Attention-deficit/hyperactivity disorder (ADHD) is a common neurodevelopmental disorder characterized by symptoms of inattention and/or hyperactivity-impulsivity, with an estimated global prevalence of 2.6% among adults.^1^ Adults with ADHD often experience substantial impairment in various aspects of their lives^2^ and have a high frequency of psychiatric comorbidities.^3^ ADHD is typically treated with stimulants (eg, methylphenidate and amphetamines) and nonstimulants (eg, atomoxetine). These medications are generally effective, particularly in the short term, but approximately 20% to 40% of patients do not achieve an adequate response.^4^ Furthermore, adverse effects may lead to treatment discontinuation,^5,6^ and long-term adherence is low. For example, only approximately 50% of patients continue methylphenidate treatment after 6 years.^7^

In recent years, the microdosing of psychedelics has gained considerable attention.^8,9,10,11^ It involves the repeated use of low doses of psychedelics, such as lysergic acid diethylamide (LSD) or psilocybin, with the aim of enhancing well-being, cognitive functions, and emotional processes.^12^ Surveys and naturalistic studies have reported that individuals also use microdosing to self-treat various disorders, including ADHD,^13^ with findings indicating a positive impact on symptoms.^14,15^ A microdose is generally considered to be one-tenth to one-twentieth of a recreational dose, an amount that does not induce significant acute perceptual changes or interfere with daily activities.^12^ Microdoses or low doses of LSD typically range from of 5 to 20 μg, with a common practice of taking the psychedelic once every 3 days over several weeks.^8,9^ However, clinical evidence from controlled studies in patients is lacking.^16^

Here, we present the first double-blind, placebo-controlled phase 2A randomized clinical trial that investigated effects of repeated low doses of LSD in adults with ADHD.

## Methods

### Study Design

This was a 6-week, double-blind, placebo-controlled, parallel-group phase 2A randomized clinical trial that was conducted at 2 centers: University Hospital in Basel, Switzerland, and at Maastricht University in the Netherlands. The trial was conducted in accordance with the Declaration of Helsinki and International Conference on Harmonization Guidelines for Good Clinical Practice. The trial was approved by local ethics committees and the respective competent authorities (eMethods in Supplement 2). Mind Medicine acted as the legal sponsor, monitored the study, and provided the data for the analysis. The study protocol is detailed in Supplement 1.

### Participants

Recruitment occurred via advertisement, referral, and word of mouth. All participants provided written informed consent. Individuals aged 18 to 65 years with a previously established (according to *DSM-IV*/*V* criteria) diagnosis of ADHD and moderate to severe current symptoms, defined by an Adult Investigator Symptom Rating Scale (AISRS)^17^ score of 26 or higher and a Clinical Global Impression Severity (CGI-S)^18^ score of 4 or higher, were eligible. Key exclusion criteria included a past or present diagnosis of psychotic disorders in the participants or their first-degree relatives, current substance use disorder, and other psychiatric or somatic disorders likely to require hospitalization or treatments that could interfere with the study. The complete list of inclusion and exclusion criteria is provided in the eMethods in Supplement 2.

### Randomization and Blinding

Eligible participants were randomly assigned in a 1:1 ratio to receive either LSD or placebo. Randomization was stratified by site, using computer-generated randomization with balanced blocks of varying sizes. The sponsor conducted the randomization and provided the allocation details to the medication producer. All study staff and participants were blinded to treatment allocation until the entire study’s conclusion.

### Study Medication

The study medication was produced by Apotheke Dr Hysek, Biel, Switzerland, in accordance with Good Manufacturing Practice (GMP). LSD was prepared as a drinking solution that contained 29 μg of GMP-grade LSD tartrate (MM-120 [Onyx Scientific]), corresponding to 20 μg of LSD base, dissolved in 1 mL of alcohol solution, 20% (volume per volume [v/v]). The placebo consisted of 1 mL of the same alcohol solution, 20% (v/v), without the active substance.

### Procedures

**Figure 1.**
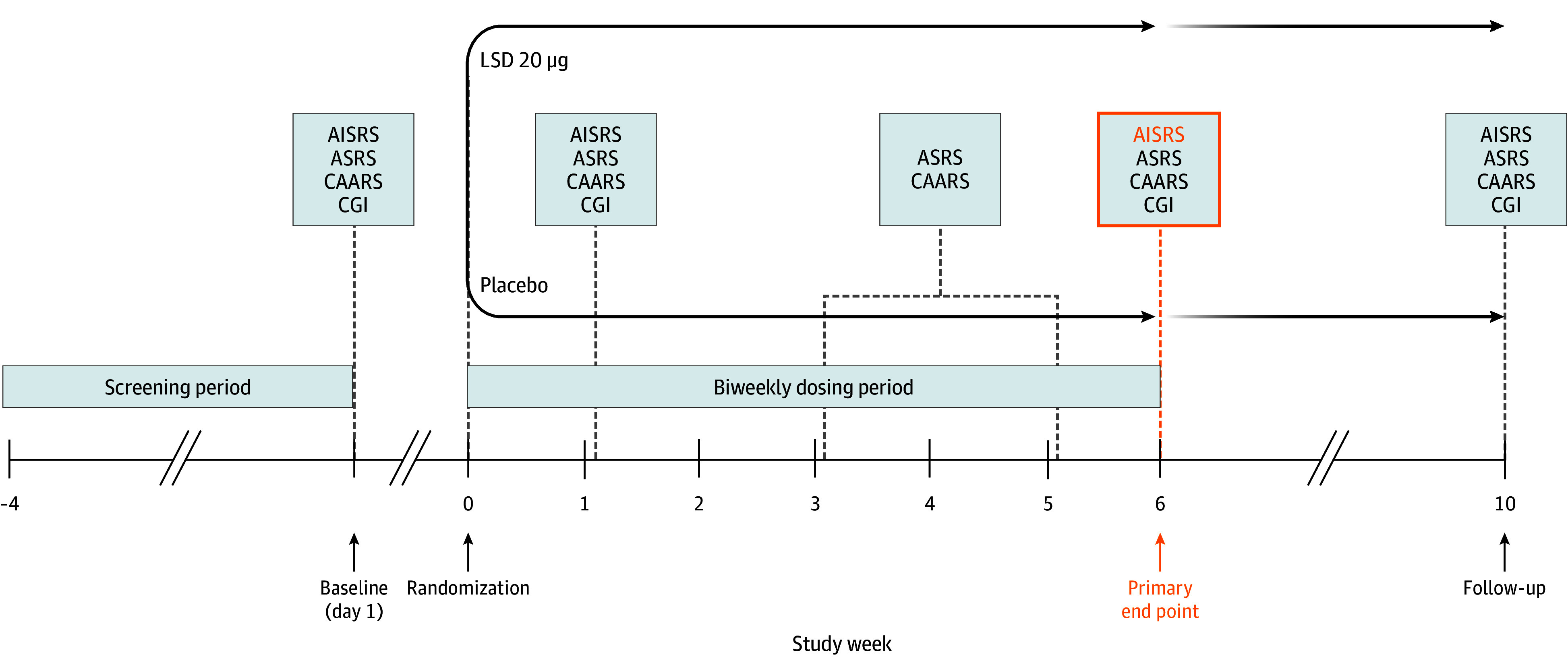
Study Design ADHD indicates attention-deficit/hyperactivity disorder; AISRS, Adult ADHD Investigator Symptom Rating Scale; ASRS, Adult ADHD Self-Report Scale; CAARS, Conners’ Adult ADHD Rating Scale; CGI, Clinical Global Impression; LSD, lysergic acid diethylamide.

Figure 1 shows the study design. A detailed schedule of all assessments is provided in eTable 1 in Supplement 2. The screening process included assessments of demographics, medical history, psychiatric history using the Mini-International Neuropsychiatric Interview (MINI),^19^ lifetime suicidal tendencies using the Columbia-Suicide Severity Rating Scale (C-SSRS),^20^ concomitant medication use, clinical laboratory analysis, urine drug screening, pregnancy test, vital signs, clinical physical examination, and an electrocardiogram.

Any ADHD or psychiatric medications and other drugs that could potentially interact with the study medication were discontinued at least 5 plasma elimination half-lives before the baseline visit, which was scheduled up to 4 weeks after screening. eTable 2 in Supplement 2 lists all prohibited medications.

Dosing occurred twice weekly over a 6-week period for a total of 12 doses. All doses were administered on-site under supervision. After the first dose, participants stayed on-site for 6 hours for clinical surveillance and to assess acute effects and pharmacokinetics. On subsequent dosing days, the participants were allowed to leave the study center immediately after drug administration. During week 10, the participants completed an end-of-study visit to assess safety and efficacy. The participants were asked to guess their treatment allocation after receiving the first and last doses to assess blinding.

### Outcome Measures

#### Efficacy

ADHD symptoms were assessed using the observer-rated AISRS^17^ and CGI-S^18^ at baseline, during treatment weeks 2 and 6, and at the end-of-study visit. The self-rated Conners’ Adult ADHD Rating Scale (CAARS)^21^ and Adult ADHD Self-Report Scale (ASRS)^22^ were administered at baseline, during weeks 2, 4, and 6, and at the end-of-study visit. Assessment occurred on-site before dosing by trained investigators. A detailed description of these measures is provided in the eMethods in Supplement 2.

#### Safety

Before each dosing, urine pregnancy tests were performed, vital signs were measured, and participants were assessed for suicidal ideations using the C-SSRS^20^ and for adverse events (AEs). Every 2 weeks during the dosing period, routine blood laboratory tests were conducted. An electrocardiogram was obtained at screening and 2 hours after the first and last dose administrations. Further details regarding safety parameters are provided in the eResults in Supplement 2.

#### Pharmacokinetics and Acute Effects

Blood plasma samples were collected 0, 0.5, 1, 2, 3, 4, and 6 hours after the first drug administration to determine the pharmacokinetics of LSD (eMethods in Supplement 2). At the same time points, a series of Visual Analog Scales (VASs) were used to measure acute effects. Additionally, the 5 Dimensional Altered States of Consciousness (5D-ASC) scale^23,24^ and 30-item Mystical Experiences Questionnaire (MEQ30)^25^ were administered 6 hours after drug administration on day 1 (on-site) and after the last dose (at home).

### Statistical Analysis

#### Sample Size and Power

A sample size of 52 participants (n = 26 participants per group) was calculated to provide 80% power to detect an effect size of 0.6 for the reduction of ADHD symptoms, measured by the AISRS, in the active treatment group compared with placebo, with a 1-sided significance level (α) of .10.

#### Primary Outcome

The a priori–determined primary outcome was the least squares mean (LSM) change in ADHD symptoms from baseline to week 6, assessed using the AISRS. This was evaluated using a Mixed Model for Repeated Measures (MMRM). Changes in AISRS scores at weeks 2 and 6 were incorporated as dependent variables. Fixed effects included treatment group, visit, visit-by-treatment group interaction, and covariates of baseline AISRS score, sex, and age. An unstructured variance-covariance matrix was used to model within-participant errors with the Satterthwaite method to approximate degrees of freedom. The analysis included all randomized participants who received at least 1 dose per the intention-to-treat principle. Changes in LSM from baseline with 95% confidence intervals were calculated for each group, and between-group LSM differences were analyzed with a 1-sided α level of .10. Missing values and dropouts were handled using multiple imputation, details of which are provided in the eAppendix and in eTable 3 in Supplement 2. Sensitivity analyses included repeating the primary analysis with a missing-at-random imputation approach and stratifying results by disease severity (see the eAppendix in Supplement 2 for more details).

#### Secondary Efficacy Outcomes

The same MMRM and imputation that were used for the primary outcome were applied to secondary efficacy outcomes with the following changes. All available time points were incorporated into the model, and a 2-tailed confidence interval with a 2-sided significance level of .05 was applied. These analyses were post hoc, and we decided to correct them for multiple testing only in the event of significant findings. Furthermore, associations between acute drug effects, guessed treatment allocation, baseline characteristics, and outcomes were explored (see also the eMethods in Supplement 2).

#### Acute Effects

Two-sided *t* tests for independent groups were used to compare acute effects between treatments on the VAS, 5D-ASC, and MEQ30.

#### Software

Statistical analyses were performed using R version 4.3.2 (R Foundation for Statistical Computing) and KNIME version 5.2.5 software (KNIME AG). MMRMs were created with the mmrm package in R,^26^ and the rbmi package in R^27^ was used for imputation.

## Results

### Participants

**Figure 2.**
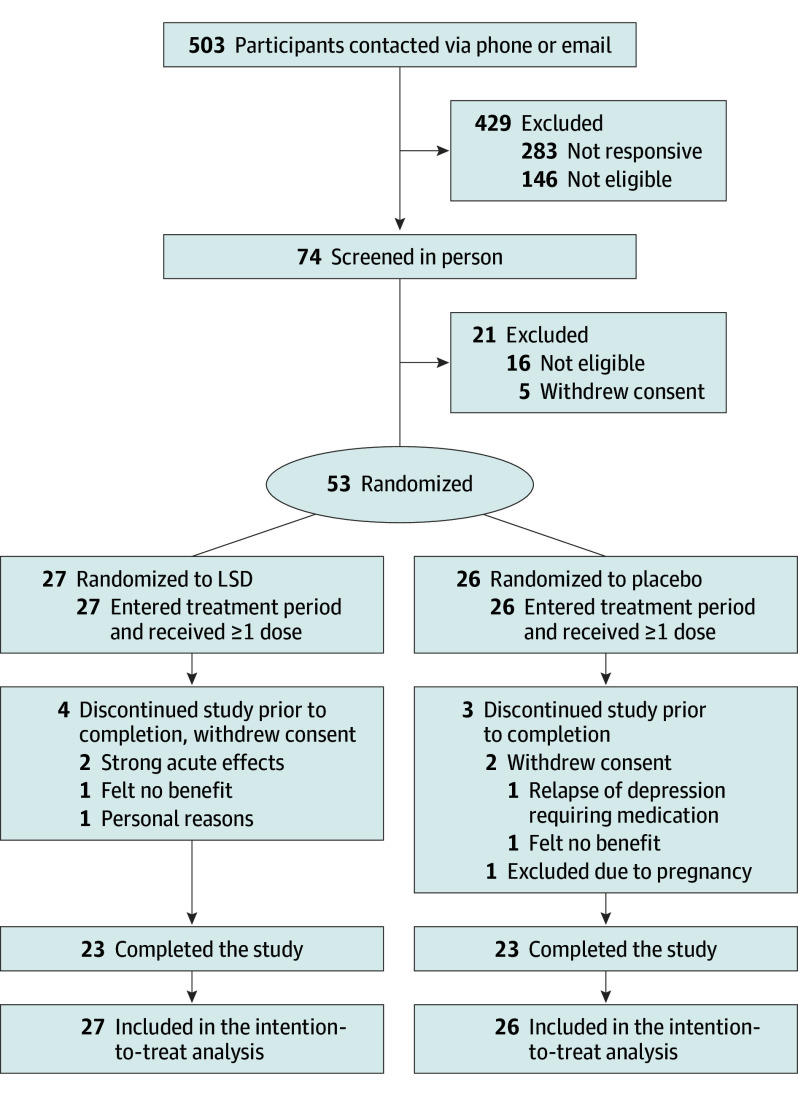
CONSORT Flow Diagram LSD indicates lysergic acid diethylamide.

The trial was conducted between December 17, 2021, and December 4, 2023. The study flow is illustrated in Figure 2. A total of 503 individuals were contacted to participate in the study. Of these, 283 did not respond or were not interested in participating. An additional 146 individuals were deemed ineligible, primarily because they lived too far away from the trial site and thus were not screened on-site. Of the 74 people who underwent in-person screening, 53 met the eligibility criteria, were randomized to LSD (n = 27) or placebo (n = 26), and received at least 1 dose of the study medication. Of all 53 participants, 50 (93%) were treated at the Basel site. Baseline characteristics of all randomized participants are summarized in Table 1. Mean (SD) participant age was 37 (12) years, and 22 participants (42%) were female. The mean (SD) AISRS score at baseline was 36 (5). Seven participants (4 in the LSD group and 3 in the placebo group) dropped out during the dosing period (eTable 3 in Supplement 2). In total, 46 participants completed the study.

**Table 1.** Participant Characteristics

Parameter	Mean (SD)		
	LSD (n = 27)	Placebo (n = 26)	Both groups (N = 53)
Age, y	40 (13)	33 (11)	37 (12)
Sex, No. (%)			
Female	13 (48)	9 (35)	22 (42)
Male	14 (52)	17 (65)	31 (58)
Weight, kg	71 (11)	75 (17)	73 (14)
Height, cm	172 (8)	176 (9)	174 (9)
ADHD medication at screening, No. (%)	10 (37)	15 (58)	25 (47)
Severe ADHD, No. (%)	13 (48)	11 (42)	24 (45)
Study site, No. (%)			
Basel	25 (93)	25 (96)	50 (94)
Maastricht	2 (7)	1 (4)	3 (6)
Highest grade of education, No. (%)			
Secondary	10 (37)	13 (50)	23 (43)
Tertiary	17 (63)	13 (50)	30 (57)
Lifetime illicit drug use, No. (%)^a^			
Cannabis	21 (78)	21 (81)	42 (79)
Hallucinogenics	16 (59)	17 (65)	33 (62)
MDMA	12 (44)	11 (42)	23 (43)
Opioids	4 (15)	1 (4)	5 (9)
Sedatives	13 (48)	8 (31)	21 (40)
Stimulants	13 (48)	14 (54)	27 (51)
ADHD scores at baseline			
AISRS	37 (6)	36 (5)	36 (5)
ASRS	47 (8)	43 (7)	45 (8)
CAARS			
A = Inattention/memory problems	23 (5)	22 (6)	22 (6)
B = Hyperactivity/restlessness	23 (7)	20 (6)	21 (7)
C = Impulsivity/emotional lability	20 (7)	17 (6)	19 (7)
D = Problems with self-concept	13 (4)	11 (4)	12 (4)
E = *DSM-IV* inattentive symptoms	18 (4)	17 (3)	17 (4)
F = *DSM-IV* hyperactive-impulsive symptoms	14 (5)	12 (5)	13 (5)
G = *DSM-IV* ADHD symptoms total	32 (8)	29 (5)	30 (7)
H = ADHD index	23 (5)	21 (4)	22 (5)
CGI-S	4.8 (0.7)	4.7 (0.6)	4.8 (0.7)

Abbreviations: ADHD, attention-deficit/hyperactivity disorder; AISRS, Adult ADHD Investigator Symptom Rating Scale; ASRS, ADHD Self-Report Scale; CAARS, Conners’ Adult ADHD Rating Scale; CGI-S, Clinical Global Impression Severity; LSD, lysergic acid diethylamide; MDMA, 3,4-Methylenedioxymethamphetamine.

^a^ Participants with any lifetime recreational use.

### Efficacy

Primary and secondary efficacy outcomes at week 6 are summarized in Table 2. The mean (SD) baseline AISRS scores were 37 (6) in the LSD group and 36 (5) in the placebo group (Table 1). As the primary end point, there was an improvement in LSM on the AISRS of −7.1 points (95% CI, −10.1 to −4.0) for the LSD group and −8.9 points (95% CI, −12.0 to −5.8) for the placebo group. The difference between groups was not significant, with an LSM difference in score change of 1.8 points (95% CI, −1.0 to ∞). In line with this, none of the sensitivity analyses of the primary outcome reached significance.

**Table 2.** Efficacy Outcomes^a^

Measure	LSD (n = 27)		Placebo (n = 26)		Difference	
	Estimate (95% CI)	*P* value	Estimate (95% CI)	*P* value	Estimate (95% CI)	*P* value
Primary MMRM						
AISRS MNAR	−7.1 (−10.1 to −4.0)	<.001^b^	−8.9 (−12.0 to −5.8)	<.001^b^	1.8 (−1.0 to ∞)	.80
Primary sensitivity MMRM						
AISRS MAR	−7.0 (−10.1 to −4.0)	<.001^b^	−8.9 (−12.0 to −5.8)	<.001^b^	1.8 (−1.0 to ∞)	.80
AISRS MNAR moderate ADHD	−8.3 (−12.1 to −4.5)	<.001^b^	−6.4 (−10.2 to −2.6)	.001^c^	−1.9 (−5.5 to ∞)	.24
AISRS MNAR severe ADHD	−6.3 (−11.2 to −1.4)	.01^d^	−11.8 (−17.0 to −6.7)	<.001^b^	5.5 (0.9 to ∞)	.94
Secondary MMRM						
AISRS	−7.1 (−10.1 to −4.1)	<.001^b^	−8.9 (−12.0 to −5.8)	<.001^b^	1.8 (−2.5 to 6.2)	.41
ASRS	−12.6 (−16.7 to −8.5)	<.001^b^	−12.6 (−16.8 to −8.3)	<.001^b^	−0.0 (−6.0 to 5.9)	.99
CAARS						
A = Inattention/memory problems	−5.9 (−8.1 to −3.6)	<.001^b^	−6.3 (−8.6 to −4.0)	<.001^b^	0.4 (−2.8 to 3.7)	.80
B = Hyperactivity/restlessness	−5.0 (−7.4 to −2.6)	<.001^b^	−5.8 (−8.3 to −3.3)	<.001^b^	0.8 (−2.7 to 4.3)	.65
C = Impulsivity/emotional lability	−6.2 (−8.3 to −4.0)	<.001^b^	−5.8 (−8.0 to −3.5)	<.001^b^	−0.4 (−3.5 to 2.7)	.80
D = Problems with self-concept	−3.2 (−4.7 to −1.7)	<.001^b^	−2.4 (−3.9 to −0.8)	.003^c^	−0.9 (−3.1 to 1.4)	.45
E = *DSM-IV* inattentive symptoms	−4.1 (−6.3 to −2.0)	<.001^b^	−4.4 (−6.6 to −2.2)	<.001^b^	0.3 (−2.8 to 3.3)	.87
F = *DSM-IV* hyperactive-impulsive symptoms	−3.5 (−5.2 to −1.7)	<.001^b^	−3.3 (−5.1 to −1.6)	<.001^b^	−0.1 (−2.6 to 2.4)	.92
G = *DSM-IV* ADHD symptoms total	−7.4 (−10.8 to −4.1)	<.001^b^	−8.0 (−11.5 to −4.6)	<.001^b^	0.6 (−4.2 to 5.4)	.80
H = ADHD index	−6.0 (−8.2 to −3.7)	<.001^b^	−5.8 (−8.1 to −3.5)	<.001^b^	−0.2 (−3.4 to 3.0)	.91
**Secondary descriptive**	**LSD (n = 23)**		**Placebo (n = 23)**		**χ^2^**	***P* value**
CGI, participants with improvement of ≥1 point	16/23	NA	15/23	NA	2.04	.15

Abbreviations: ADHD, attention-deficit/hyperactivity disorder; AISRS, Adult ADHD Investigator Symptom Rating Scale; ASRS, ADHD Self-Report Scale; CAARS, Conners' Adult ADHD Rating Scale; CGI, Clinical Global Impression Severity; LSD, lysergic acid diethylamide; MAR, missing at random; MNAR, missing not at random; MRMM, mixed-effect models for repeated measures; NA, not applicable.

^a^ Primary and secondary efficacy outcomes after 6 weeks of treatment. Estimates are least squares mean change from baseline values determined by MRMM. Models for the primary outcome used data from weeks 2 and 6 and 1-sided testing with a significance level of <.1 for group comparison; models for the secondary outcomes included all available time points and 2-sided testing with a significance level of <.05. χ^2^ and *P* values for descriptive outcomes refer to the McNemar test.

^b^ *P* < .001.

^c^ *P* < .01.

^d^ *P* < .05.

Secondary efficacy analyses tested 2-sided showed no significant differences between treatment groups. Both groups presented significant improvements in the change from baseline over time across all outcome measures. Results for secondary efficacy outcomes at all time points are detailed in eTables 5-16 and eFigures 1-3 in Supplement 2.

### Blinding

A total of 37 of 46 participants (80%) guessed they were allocated to the LSD group after the last dose (21 of 22 participants in the LSD group and 16 of 24 participants in the placebo group). Overall, 29 participants (63%) correctly guessed their allocation (21 of 22 for the LSD group and 8 of 24 for the placebo group; see also eTable 4 in Supplement 2). After 6 weeks, participants who believed they received LSD showed nominally larger LSM reductions compared with those who thought they received placebo (eFigure 4 in Supplement 2).

### Acute Effects

Acute effects are shown in eTables 17 and 18 and eFigures 5-7 in Supplement 2. The mean (SD) maximal responses on the VAS for any drug effect were 52% (33) in the LSD group and 22% (29) in the placebo group (*P* < .001). The mean (SD) 5D-ASC total scores were 13% (11) in the LSD group and 4.9% (7.8) in the placebo group (*P* = .005). The mean (SD) MEQ30 total scores were 22 (20) in the LSD group and 10 (16) in the placebo group (*P* = .02). Several further VAS items and subscales of the 5D-ASC and MEQ30 also showed significantly higher acute effects of LSD compared with placebo (eTable 18 in Supplement 2).

### Pharmacokinetics

Pharmacokinetic parameters for LSD are summarized in eTable 19 and eFigure 8 in Supplement 2.

### Safety

A total of 124 AEs occurred in the LSD group, and 64 occurred in the placebo group. No serious AEs and no deaths were recorded during the study. The 5 most common treatment-related AEs were headache, nausea, fatigue, insomnia, and visual alterations. These AEs were more frequent in the LSD group than in the placebo group (23 vs 8). Complete lists of all AEs and related AEs are provided in eTables 20 and 21 in Supplement 2. One participant in the placebo group was excluded after a positive pregnancy test. Additionally, 2 participants in the LSD group dropped out after the first dose and after 5 doses, respectively, because of uncomfortably strong acute effects or effects that impaired daily activities. No newly occurring suicidal ideations were reported during the dosing period (eTable 22 in Supplement 2). Electrocardiographic parameters are summarized in eTable 23 in Supplement 2, showing no differences between LSD and placebo over time. Acute effects on blood pressure and heart rate are illustrated in eFigure 9 in Supplement 2, with no significant differences between treatment groups. Laboratory parameters are detailed in eTable 24 in Supplement 2. None of the laboratory values outside the reference range were attributed to the study drug.

## Discussion

To our knowledge, this is the first double-blind, placebo-controlled randomized clinical trial to investigate effects of repeated low-dose (20 μg) LSD administration in adults with ADHD. The study did not meet its predefined primary end point (ie, change in ADHD symptoms from baseline to week 6 of treatment). Generally, LSD did not improve ADHD symptoms over placebo on any of the measures. LSD was well-tolerated in the outpatient setting. Treatment-related adverse reactions were mostly mild and included headache, nausea, fatigue, insomnia, and visual alterations.

A study in healthy volunteers who received 10 μg LSD every 3 days for 6 weeks found that LSD microdosing was safe in adult men, but the study noted increases in treatment-related anxiety.^28^

Pharmacokinetic parameters demonstrated rapid absorption of LSD, with maximal plasma concentrations reached after 1.3 hours, and were generally in line with data from previous studies in healthy participants.^29^

Overall, LSD produced substantial and significantly stronger acute subjective effects compared with placebo that were qualitatively similar to high doses of LSD, but to a lesser extent.^30^ Controlled studies in healthy participants have reported that perceptual threshold doses of LSD are around 10 μg of LSD base, with 20 μg producing mild effects that were comparable to, but generally lower than, those in the present study.^29,31,32,33^ This study’s 20–μg dose of LSD base is at the upper end of the microdosing range and might rather be considered a low dose instead. The same dose has been shown to produce stronger acute subjective effects in healthy participants, with high self-rated depressive symptoms compared with participants with low ratings.^34^ Overall, the findings indicate potentially greater acute subjective effects of low doses of LSD in patient populations compared with healthy individuals. High interindividual variability in effects of low doses of LSD on mood and cognition has been observed in healthy people.^35^ In the present study, 2 participants in the LSD group stopped treatment because of strong acute effects. One participant described the effects as very intense and uncomfortable and withdrew after the first dose. The other participant found the effects generally pleasant but felt too impaired to perform daily activities and withdrew after 5 doses.

In this proof-of-concept study, a rather high microdose was selected to increase the likelihood of detecting a positive response and efficacy. Accordingly, we consider it unlikely that the dose was too low to be efficacious. In contrast, we cannot exclude the possibility that a lower dose or a different dosing interval could have yielded beneficial effects on ADHD symptoms, although the present study does not suggest such benefits.

Both the LSD and placebo groups showed improvements in self-rated and observer-rated ADHD measures over the course of treatment, which persisted for 3 weeks after the dosing period. Compared indirectly to methylphenidate and atomoxetine in other trials, the verum group showed slightly lower and the placebo group comparable to slightly higher responses.^36,37^ Most participants, including the majority of those who received placebo, believed that they had been allocated to the LSD group at the end of the dosing period, and they presented nominally larger ADHD symptom reductions on most outcome measures than those who did not.

Relevant placebo responses have been shown for other medications in previous clinical trials with patients with ADHD.^38,39,40,41^ The present study design, with multiple dosing visits and extensive symptom assessments, may have contributed to the placebo response. Additionally, media reports of potential benefits of psychedelics in psychiatric patients could have heightened expectations. A high motivation of participants can be assumed by their commitment to commute to the study center twice weekly over 6 weeks for dosing on-site.

Our findings are consistent with a previous study that used a self-blinding approach in healthy participants,^42^ showing that subjective well-being was enhanced in both recreational microdosing and placebo groups.^42^ These findings indicate that observed benefits of psychedelic microdosing may be attributable more to expectancy than to pharmacological effects of the psychedelic itself. Exploratory analysis in our study did not identify predictors of favorable outcomes, such as acute effect strength, ratings on the 5D-ASC, age, or sex.

Altogether, these results do not confirm possible positive effects of low doses of LSD on ADHD symptoms as suggested by user surveys^15^ or naturalistic studies.^14^ This discrepancy underscores the need for randomized placebo-controlled trials when validly assessing potential benefits of low-dose psychedelics that are likely prone to a placebo response and expectancy bias.

### Strengths and Limitations

The present experimental trial of low-dose psychedelics in a patient population with clinically relevant symptoms has several strengths, including a placebo-controlled design, supervised dosing, and comprehensive assessments of safety, pharmacokinetics, and both self-rated and observer-rated efficacy. Nevertheless, our study has several limitations. The trial was powered to find a rather large effect size, while very small effects may have been missed. Although a multicenter trial, 1 site enrolled 95% of the participants, which was due to logistical problems. Expectancy was not systematically assessed. We only tested twice-weekly dosing with a relatively high (for microdosing) and fixed dose of LSD, which may not account for potential interindividual variability in the response to psychedelics. Daily dosing, alternate-day dosing, or titration^43^ to produce the desired effect without impairing activities of daily life may produce different results. Furthermore, other ADHD-related outcomes, such as emotion regulation, were not comprehensively addressed.

## Conclusions

In conclusion, although repeated low-dose LSD administration was safe in an outpatient setting, it failed to demonstrate efficacy compared with placebo in improving ADHD symptoms among adults.

## Notes

Supplement 1. Trial Protocol

Supplement 2. eTable 1. Study Schedule eMethods. Supplementary Methods eTable 2. List of Prohibited Medications eAppendix. Statistical Analysis eTable 3. Dropouts eResults. Supplementary Results eTable 4. Guessed and Actual Allocation eTable 5. MMRM of the Adult ADHD Investigator Symptom Rating Scale AISRS eTable 6. MMRM of the ADHD Self-Report Scale ASRS eTable 7. MMRM of the Conners' Adult ADHD Rating Scale A (Inattention/Memory Problems) eTable 8. MMRM of the Conners' Adult ADHD Rating Scale B (Hyperactivity/Restlessness) eTable 9. MMRM of the Conners' Adult ADHD Rating Scale C (Impulsivity/Emotional Lability) eTable 10. MMRM of the Conners' Adult ADHD Rating Scale D (Problems with Self-Concept) eTable 11. MMRM of the CAARS E (DSM-IV Inattentive Symptoms) eTable 12. MMRM of the Conners' Adult ADHD Rating Scale F (DSM-IV Hyperactive-Impulsive Symptoms) eTable 13. MMRM of the Conners' Adult ADHD Rating Scale G (DSM-IV ADHD Symptoms Total) eTable 14. MMRM of the Conners' Adult ADHD Rating Scale H (ADHD Index) eTable 15. Descriptive Statistics of Efficacy Outcomes (Change From Baseline) eTable 16. Descriptive Statistics of Efficacy Outcomes (Absolute Values) eFigure 1. AISRS, ASRS, MMRM eFigure 2. Clinical Global Impression-Severity eFigure 3. Conners' Adult ADHD Rating Scale MMRM eFigure 4. Outcomes After 6 Weeks Stratified by Guessed Allocation MMRM eTable 17. Acute Effects Measured by Visual Analog Scales eFigure 5. Acute Effects Measured by Visual Analog Scales eFigure 6. Acute Effects Measured by Visual Analog Scales (Spaghetti Plots) eTable 18. Acute Effects on the 5 Dimensions of Altered States of Consciousness Scale and Mystical Experiences Questionnaire eFigure 7. Acute Effects on the 5 Dimensions of Altered States of Consciousness Scale and Mystical Experiences Questionnaire eTable 19. Pharmacokinetic Parameters of LSD (20 µg) Determined by Non-Compartmental Analysis eFigure 8. Pharmacokinetics of LSD and O-H-LSD eTable 20. Adverse Events eTable 21. Related Adverse Events eTable 22. Suicidal Ideations eTable 23. Electrocardiographic Parameters eFigure 9. Blood Pressure and Heart Rate During the First Dose Administration eTable 24. Laboratory Values at Different Visits eReferences.

Supplement 3. Data Sharing Statement
