# Supplementary material for: Safety and Efficacy of Repeated Low-Dose LSD for ADHD Treatment in Adults: A Randomized Clinical Trial
Source: JAMA Psychiatry. 2025 Mar 19;82(6):555–62. doi: 10.1001/jamapsychiatry.2025.0044 (PMC11923771; doi:10.1001/jamapsychiatry.2025.0044)
Supplement: Supplement 1. — Trial Protocol [file jamapsychiatry-e250044-s001.pdf]

SUPLEMENT 1

CLINICAL TRIAL PROTOCOL

Safety and efficacy of repeated low-dose LSD for ADHD treatment in adults: a multicenter, randomized, double-blind, placebo-controlled Phase 2A trial

Sponsor Protocol Number:	MMED007
Investigational Product:	D-LYSERGIC ACID DIETHYLAMIDE (LSD) D-TARTRATE (MM-120)
Sponsor:	Mind Medicine, Inc.<br>World Trade Center, Suite 8500 New York,<br>NY 10007, USA
Protocol Version/Date:	Version 5, 09-MAR-2022

TABLE OF CONTENTS

1. PROTOCOL SYNOPSIS 4
2. LIST OF ABBREVIATIONS 10
3. INTRODUCTION 12
   3.1. Disease Background 12
   3.2. Drug Background 12
   3.3. Rationale for Investigating Repeated Low-dose LSD Administration in Subjects with ADHD 12
   3.4. Study Drug 13
   3.5. Recent and Current Clinical Studies with LSD in Healthy Subjects and Subjects 14
4. STUDY OBJECTIVES 17
   4.1. Primary Objective 17
   4.2. Secondary Objectives 17
   4.3. Exploratory Objective 17
5. STUDY PLAN AND PROCEDURES 17
   5.1. Overall Study Design 17
   5.2. Endpoints 18
6. SUBJECT SELECTION CRITERIA 19
   6.1. Inclusion Criteria 19
   6.2. Exclusion Criteria 20
7. STUDY CONDUCT 21
   7.1. Subject Enrolment and Randomization 21
   7.2. Procedures for Handling Incorrectly Randomized Subjects 22
   7.3. Treatments 22
   7.4. Identity of Investigational Medicinal Product 22
   7.5. IMP, Concomitant, and Post-Study Treatment(s) 23
   7.6. Prohibited concomitant medications 23
   7.7. Intra-subject Variability due to LSD Metabolism 24
   7.8. Withdrawal from Study 24
   7.9. Audits and Inspections 24
8. COLLECTION OF STUDY VARIABLES 25
   8.1. Schedule of Events 25
   8.2. Table 1: Schedule for study specific assessments 25
   8.3. Visits and Assessments 27
9. SAFETY 31
   9.1. Physical Risks to the Subjects 31
   9.2. LSD-Specific Toxicity Considerations 31
   9.3. Safety Monitoring 33
   9.4. Safety Procedures During the Session 34
   9.5. Risk Benefit Assessment 34
   9.6. Safety Definitions 35
   9.7. Causality 35
   9.8. Documentation 36
   9.9. Medical Follow-Up of Adverse Events 36
   9.10. Screening Procedures 36
   9.11. Urgent Safety Measures 37
   9.12. Special Situations 38
10. COLLECTION OF STUDY VARIABLES 38
   10.1. Recording of Data 38
   10.2. Clinical Laboratory Assessment 38
   10.3. Pharmacokinetics 40
   10.4. Pharmacodynamics 40
11. BIOLOGICAL SAMPLING PROCEDURES 40
   11.1. Volume of Blood 40
   11.2. Handling, Storage and Destruction of Biological Samples 41
12. ETHICAL AND REGULATORY REQUIREMENTS 41
   12.1. Ethical Conduct of Study 41
   12.2. Subject Data Protection 41
   12.3. Ethics and Regulatory Review 41

12.4. Informed Consent ... 41
12.5. Changes to Protocol and Informed Consent Forms ... 41
12.6. Audits and Inspections ... 42
13. STUDY MANAGEMENT ... 42
13.1. Pre-study Activities ... 42
13.2. Data Management by a Delegate ... 43
14. EVALUATION AND CALCULATION OF VARIABLES ... 43
14.1. Calculation or Derivation of Safety Variable(s) ... 43
14.2. Other Significant Adverse Events ... 43
15. STATISTICAL METHODS AND SAMPLE SIZE ... 44
15.1. Data Analysis ... 44
15.2. Analysis Sets ... 44
15.3. Subject demographics and other baseline characteristics ... 44
15.4. Treatment Exposure ... 44
15.5. Prior and Concomitant Medication ... 45
15.6. Efficacy Analyses ... 45
15.7. Safety analyses ... 47
15.8. Pharmacodynamics ... 47
16. IMPORTANT MEDICAL PROCEDURES TO BE FOLLOWED BY THE INVESTIGATOR 47
16.1. Medical Emergencies and Contacts ... 47
16.2. Overdose ... 47
16.3. Pregnancy ... 48
16.4. Maternal Exposure ... 48
16.5. Paternal Exposure ... 48
17. REFERENCES ... 48
18. APPENDICES ... 55
18.1. APPENDIX 1 LIST OF PROHIBITED MEDICATIONS, SUPPLEMENTS, AND OTHER THERAPIES ... 55
18.2. APPENDIX 2 DEFINITION OF WOMEN OF CHILDBEARING POTENTIAL (WOCBP) AND ACCEPTABLE HIGHLY EFFECTIVE METHODS OF CONTRACEPTION ... 57
18.3. APPENDIX 3 LIST OF CYTOCHROME P450 2D6 (CYP2D6) INHIBITORS ... 58

1. PROTOCOL SYNOPSIS

**Study title**	Safety and efficacy of repeated low dose D-lysergic acid diethylamide (LSD) D-Tartrate (MM-120) as treatment for ADHD in adults: a multi-center, randomized, double-blind, placebo- controlled Phase 2a Proof of Concept Trial
**Investigational Medicinal Product**	D-LYSERGIC ACID DIETHYLAMIDE (LSD) D-TARTRATE (MM-120)
**Phase of Development**	Phase 2a
**Study Population**	Fifty-two (52) eligible subjects aged ≥ 18 and ≤ 65 years with attention-deficit/hyperactivity disorder (ADHD) either previously diagnosed or diagnosed at screening
**Sponsor**	Mind Medicine, Inc.
**Study sites**	University Hospital Basel, Switzerland and Maastricht University, The Netherlands
**Objectives**	**Primary Objective**<br>To assess the treatment efficacy vs placebo of repeated low doses (20 µg) of MM-120 for six weeks in adult subjects with ADHD measured by Adult Attention Deficit Investigator Symptom Rating Scale (AISRS).<br>**Secondary Objectives**<br>1. To assess treatment efficacy vs placebo measured by change from baseline in AISRS after 1 week of treatment.<br>2. To assess treatment efficacy vs placebo based on the proportion of subjects who experience at least a 1-point decrease in the Clinical Global Impression - Severity of Illness Scale (CGI-S).<br>3. To assess treatment efficacy vs placebo measured by change from baseline in CGI-S.<br>4. To assess the safety and tolerability by Adverse Event (AE) and Serious Adverse Event (SAE) assessment.
**Study Design**	This study is a multi-center, randomized, double-blind, placebo-controlled Phase 2a study of low dose MM-120 (20 µg) compared with a placebo administered for 6 weeks (twice a week on a 3/4-day schedule [± 1 day]).<br>Low dose MM-120 (20 µg) is about 20% of the dose typically consumed for recreational psychedelic purposes.<br>There will be a 1:1 randomization, double-blind, to MM-120 or placebo with the aim to reach 26 evaluable subjects in each of the 2 arms at Week 6. <u>There will be 2 arms:</u><br>● Arm 1-Placebo: a total of 26 subjects will receive a placebo identical in appearance to the investigational medicinal product (IMP) administered orally twice weekly (e.g., Tuesday/Friday) for 6 weeks.<br>● Arm 2-MM-120: a total of 26 subjects will receive 20 µg of MM120 administered orally twice weekly for 6 weeks.<br>Subjects will be evaluated at Baseline, Week 1, Week 6 and at Week 10, for AISRS and CGI-S by trained study personnel; the primary endpoint will be based on evaluation of AISRS at Baseline and at Week 6.<br>Screening and Baseline visits:

	● Potential study subjects who provide informed consent will have eligibility evaluated/confirmed at 2 visits: 1) Screening (Visit 1); 2) Baseline (Visit 2). The screening visit will occur up to 4 weeks prior to Baseline visit. An assessment will be made to confirm or make the ADHD diagnosis with the Mini International Neuropsychiatric Interview (MINI), and other eligibility criteria. The subject must have an AISRS score of ≥ 26, and a CGI-S of ≥ 4 at screening and must meet all other eligibility criteria.<br>● The baseline visit will occur at the clinic before Day 1 and the AISRS score will be confirmed. If there is a ≥13-point change in the AISRS between the screening visit assessment and the baseline assessment, or if the subject does not return for the baseline AISRS, the subject will not be randomized and will be terminated from the study. Eligible subjects will be randomized on Day 1 to either 20 µg of MM-120 or to matching placebo and begin the double-blind treatment period on Day 1.<br>Test days:<br>● The double-blind treatment period will be 6 weeks in duration, with dosing twice weekly (every 3 to 4 days). Day 1 will be the first day of dosing. At the end of 6 weeks, subjects will be assessed by a study physician. All subjects and site staff will remain blinded to the treatment arm.<br>Follow-up:<br>● There will be a single follow-up visit to occur 4 weeks after the last double-blind treatment visit (i.e., at Week 10).
**Randomization**	This is a double-blind study. Subjects will receive either MM-120 as a single dose containing 20 µg (D-LSD D-tartrate) freebase equivalent or matching placebo.
**Inclusion Criteria**	All inclusion criteria will be based on the judgment of the Investigator. For inclusion in the study, subjects must meet all of the following criteria:<br>1. Ability and willingness to provide written, informed consent prior to initiation of any study-related procedures and to adhere to all study requirements. NOTE: The subject (i.e., not a legally authorized representative) must be cognitively able to understand the requirements of the study and provide the informed consent<br>2. Age ≥ 18 and ≤ 65 years at Screening.<br>3. Subjects with the diagnosis of Diagnostic and Statistical Manual ofMental Disorders-5 (DSM-5) ADHD, as determined by clinical evaluation and confirmed by structured interview (MINI).<br>4. AISRS total score of ≥ 26 at screening.<br>5. CGI-S score of ≥ 4 at screening.<br>6. Must be willing to receive IMP dose twice weekly. On day 1, the subject will come to the site clinic and must be willing to take a taxi or public transportation home or be accompanied by a caregiver and not drive a car, use heavy equipment, or participate in any other dangerous activity for the remainder of the day after receiving IMP (NOTE: at any protocol visit after Day 1 dosing, dosing visits may occur at the subject's home at the discretion of the PI, conducted by one of the study investigators or delegate and administered under supervision followed by the performance of the same procedures done at the clinic including safety monitoring. If early withdrawal is considered due to any safety issue identified, the Sponsor's medical monitor should be notified. If a remote visit is conducted due to any reason related to the COVID-19 pandemic, notification must be sent to the Medical Monitor's dedicated email address and Urgent Safety Measures as outlined in this protocol must be followed.)<br>7. Must be willing to refrain from more than 6 standard alcoholic drinks

	per week (1 standard drink corresponds to 0.1 L wine, 0.3 L beer, or 4 cL liquor), more than 10 cigarettes a day, and more than 2 cups of coffee a day throughout the study treatment period (6 weeks) and until the last study visit is complete (EoS or ET).
**Exclusion Criteria**	Subjects should not be included in the study if any of the following exclusion criteria are met:<br>1. Past or present diagnosis of a primary psychotic disorder or first degree relative with a psychotic disorder.<br>2. Past or present bipolar disorder (DSM-5).<br>3. Other current psychiatric disorders that, in the opinion of the Investigator or medical supervisor, may confound the results of the study (e.g., obsessive-compulsive disorder, dysthymic disorder, panic disorder, dissociative disorder, anorexia nervosa or bulimia nervosa)<br>4. Subjects with past (> 1 month prior to the screening visit) or present history of substance use disorder (except nicotine, provided subject does not smoke more than 10 cigarettes a day).<br>5. Somatic disorders including Central Nervous System (CNS) involvement of cancer, severe cardiovascular disease, untreated hypertension, severe liver disease (liver enzyme increase by more than 3x the upper limit of normal except unconjugated hyperbilirubinemia due to Gilbert's Disease, per Investigator), severely impaired renal function (estimated creatinine clearance < 50 mL/min by CKD-EPI formula), or anything else that, in the judgment of the Investigator or medical supervisor, poses too great a potential for side effects.<br>6. Any lifetime history of suicide attempt; or recent (within 6 months prior to the screening visit) active suicidal thoughts or ideation (defined as a suicidal ideation score of 2 or greater in the Columbia-Suicide Severity Rating Scale [C-SSRS]); or endorsement of any suicidal behavior on the C-SSRS within the past 6 months prior to the screening visit.<br>7. Likely to require psychiatric hospitalization during the course of the study.<br>8. Once consent is signed, subject not willing or safely able to stop any prescription or non-prescription ADHD medications during screening and prior to the baseline visit through final study visit, EoS or ET. *A list of prohibited medications is provided in Appendix 1.*<br>9. Plan to start, stop, or alter the use of any medications, supplements, or other therapeutics from Baseline until the EoS or ET (see Appendix 1 for list of prohibited medications).<br>10. Plan to start, stop or alter the use of psychotherapy, massage, meditation, acupuncture, hypnosis, yoga, or other similar therapy/activity from the time of providing informed consent until EoS or ET.<br>11. Use of potent CYP2D6 inhibitors; moderate CYP2D6 inhibitors by Investigator discretion (see Appendix 3).<br>12. Likely to need any psychiatric medications with the potential to confound interpretation of study results or impact safety, at the discretion of the Investigator, in the 10 weeks following Baseline up to EoS or ET.<br>13. Use of investigational medication/treatment in the past 30 days prior to the screening visit.<br>14. Subjects with a positive urine drug screen (with the exception of THC or metabolites) at Screening OR Baseline.<br>15. Clinically significant abnormal baseline laboratory, VSs, and ECG values which include the following:<br>o Have evidence of clinically significant hepatic disorder (e.g., alanine aminotransferase [ALT] or aspartate aminotransferase [AST] >3X ULN (except for Gilbert's disease).

	o Any clinically significant abnormal metabolic or hematologic screen, per Investigator or medical supervisor decision.<br>o Exclusionary blood pressure: > 140 mm Hg (systolic) or > 90 mm Hg (diastolic); heart rate <45 beats/minute or >90 beats/minute after an approximately 5-minute supine or semi-supine rest. NOTE: If the first measurement of a subject's heart rate is >90 beats/minute, a second recording is allowed after an additional approximately 5-minute supine rest.<br>o Exclusionary ECG parameters: QTcF > 450 msec (men), QTcF >470 msec (women).<br>o Any clinically significant abnormal electrocardiogram (ECG) finding (e.g., uncontrolled atrial fibrillation, ischemia) at Screening (Visit 1) or Baseline (Visit 2), as determined by the Investigator or medical supervisor (in consultation with a cardiologist, if needed).<br>16. Any other condition, therapy, laboratory abnormality, or other circumstance that, in the opinion of the Investigator or medical supervisor, may pose additional risk to the subject from participation in the study, may interfere with the subject's ability to comply with study procedures, may make participation in the study not in the subject's best interest or may confound the results of the study.<br>17. Prior history or ongoing neuropsychiatric signs or symptoms associated with COVID-19 such as development of, or current disorder, during or after a COVID-19 infection including anxiety, memory loss, confusion, depression, delirium, agitation, or psychosis.<br>18. Women of childbearing potential (WOCBP) (i.e., physiologically capable of becoming pregnant) who are unwilling or unable to use a highly effective method of contraception, as defined in Appendix 2, for the duration of the study, OR Men physiologically capable of fathering a child who are sexually active with WOCBP but are unwilling or unable to use barrier contraception (e.g., condom with or without spermicidal cream or jelly) for the duration of the study<br>19. NOTE: See Appendix 2 for definitions of WOCBP and highly effective methods of contraception and for information about unacceptable methods of contraception.<br>20. Women who are currently pregnant or breastfeeding or plan to become pregnant or breastfeed during the study.<br>21. Men who plan to donate sperm during the study.<br>22. Use of weight loss drugs within 21 days of screening until the EoS or ET.<br>23. Subjects who are either unable or unwilling to consume alcohol in any amount (including due to religious or personal reasons).<br>24. Subjects who have a change in AISRS score of ≥13-point change between screening and baseline visits.
**Investigational Product**	Subjects will be treated as described below with doses of MM-120 or placebo.<br>Arm 1 – placebo (Ethanol 0.16 g aqua pur ad 1 mL), twice weekly for 6 weeks, for a total of 12 doses.<br>Arm 2 – MM-120 (LSD-tartrate 0.029 mg [corresp. 0.02 mg LSD], ethanol 0.16 g, aqua pur ad 1 mL), twice weekly for 6 weeks for a total of 12 doses.<br>Patients and study staff will remain blinded to treatment arm.

**Protocol Adherence**	An education program will be in place for subjects and additional for subjects who miss a dosing appointment.<br>IMP administration will be performed at the site on Day 1 or at home after Day 1 per PI decision and supervised by study personnel. On Day 1, subjects must come to the site clinic and be willing to take a taxi or public transportation home or be accompanied by a caregiver. Subjects will be instructed that they cannot drive or operate heavy machinery or any other task that could be dangerous when impaired for the remainder of the day after receiving IMP.
**Endpoints**	**Primary endpoint:**<br>Mean change from baseline in ADHD symptoms, as assessed by the AISRS after 6 weeks of treatment. The AISRS total score consists of 18 items from the original Attention-Deficit/Hyperactivity Disorder - Rating Scale (ADHD-RS), which were derived based on DSM-5 criteria for ADHD. The ADHD-RS includes 9 items that address symptoms of inattention, and 9 items that address symptoms of impulsivity and hyperactivity. Each item is rated from 0 to 3. The AISRS total score can range from 0 to 54. A higher score corresponds to a worse severity of ADHD.<br>**Secondary endpoints:**<br><u>ADHD-related endpoints</u><br>● Key secondary endpoint: change from baseline in AISRS after 1 week (2 doses) of treatment.<br>● Occurrence of subjects who experience at least a 1-point decrease in the CGI-S<br>● Change from baseline in CGI-S after 1 week (2 doses) of treatment and after 6 weeks of treatment.<br>● Change from baseline in subject self-assessment by the Adult ADHD Self-Report Scale (ASRS) and Connors' Adult ADHD Rating Scale (CAARS).<br><u>Safety Endpoints</u><br>● Vital signs (supine blood pressure, heart rate)<br>● 12-lead safety ECG<br>● Adverse events (e.g., psychological and/or physiological adverse events)<br>● Columbia-Suicide Severity Rating Scale (C-SSRS)<br>● Safety laboratory evaluation and Urine pregnancy testing<br><u>Pharmacokinetic outcomes</u><br>● Blood samples for PK evaluation will be collected for 6 hours after the administration of the IMP on day 1.<br>● Cmax, Tmax, AUC, T1/2<br><u>Acute effects (Pharmacodynamic) endpoints</u><br>● 5 Dimensions of Altered States of Consciousness scale (5DASC),<br>● Mystical Experience Questionnaire (MEQ),<br>● Drug effect Visual Analog Scale (VAS)<br>**Exploratory Endpoints:**<br>● Sleep quality/ duration rating will be evaluated based on daily diary entries and details of the analysis will be included in the SAP.

**Statistical Methods**	**Statistical Design and Primary Endpoint**<br>This is a randomized, double-blind, placebo-controlled, Phase 2 trial that will evaluate the effects of MM-120 in adult subjects with ADHD. The primary endpoint is the change from baseline in AISRS score after 6 weeks of treatment. The effect will be estimated using the mean improvement in the active arm vs the placebo arm.<br>Eligible subjects that consent to participate will be randomized in a 1:1 allocation.<br><u>Determination of Sample Size</u><br>Randomizing 26 subjects per arm, if the true efficacy of MM-120 reaches a standardized mean improvement over placebo of 0.60, will allow rejection of the null hypothesis that active is not better than placebo at a one-sided p-value < 0.10 with 80% power.<br>The minimum estimated standardized effect size resulting in a p-value < 0.10 is 0.38 while an estimated standardized effect size < 0.56 will result in a one-sided p-value < 0.025.
**Duration of Study**	The duration of a subject's participation in the trial is up to 14 weeks, including:<br>● Screening period: up to 4 weeks prior to randomization in each cohort.<br>● Treatment period: 6 weeks of blinded treatment<br>● Follow-up: 4 weeks after the end of treatment.
**Lab Parameters + ECG**	● Hematology<br>● Serum biochemistry<br>● Urine pregnancy test<br>● Urine drug screen<br>● 12-lead safety ECG.

123 ## 2. LIST OF ABBREVIATIONS

ADHD	Attention-deficit/hyperactivity disorder
AE	Adverse event
AISRS	Adult ADHD investigator symptom rating scale
ADHD-RS	Attention-deficit/hyperactivity disorder-rating scale
ALT	alanine aminotransferase
AST	aspartate aminotransferase
ASRS	Adult attention-deficit/hyperactivity disorder self-reporting rating scale
AUC	area under the curve concentration in plasma
BMI	body mass index
BP	blood pressure
CBD	cannabidiol
CAARS	Connors' adult ADHD rating scale
CAARS-L-SR	Connors' adult ADHD rating scale self-report long form
CAARS-S-OR	Connors' adult ADHD rating scale observer-rated short screening
CGI-S	Clinical global impression-severity scale
CKD-EPI	Chronic Kidney Disease Epidemiology Collaboration (CKD-EPI) equation
cGMP	current Good Manufacturing Practices
Cmax	maximum serum concentration
COVID-19	coronavirus disease 2019
CRF	Case report form
CRP	C-reactive protein
CSR	clinical study report
C-SSRS	Columbia-suicide severity rating scale
C-SSRS-SLV	Columbia-Suicide Severity Rating Scale-Since Last Visit
CYP	cytochrome P450
5D-ASC	5 dimensions of altered states of consciousness scale
DMT	dimethyltryptamine
DSM-5	Diagnostic and statistical manual version 5
ECG	Electrocardiogram
eCRF	Electronic case report form
EDC	Electronic data capture
EOS	End of Study
eGFR	estimated glomerular filtration rate
ET	Early Termination
FSH	follicle-stimulating hormone
GCP	Good clinical practice
GDP	Good Distribution Practice
GGT	gamma-glutamyl transferase
HR	heart rate
IB	Investigator's brochure
ICF	informed consent form
ICH	International Conference on Harmonisation
IE	intercurrent event
IEC	Institutional Ethics Committee
IMP	Investigational medicinal product

LSD	Lysergic acid diethylamide
MAOI	monoamine oxidase inhibitor
MCH	mean corpuscular hemoglobin
MCHC	mean corpuscular hemoglobin concentration
MCV	mean corpuscular volume
MDMA	3,4-methylenedioxymethamphetamine
MEQ30	Mystical experience questionnaire 30 items
MINI	Mini International Neuropsychiatric Interview
Ml	milliliter
MM-120	Mind Medicine, Inc. compound dose for D-lysergic acid diethylamide D-tartrate
PK	Pharmacokinetic
PV	Pharmacovigilance
QTcF	QT interval corrected using Fridericia’s formula
RDW	red cell distribution width
RR	respiratory rate
SAE	Serious adverse events
SNDRI	serotonin-norepinephrine-dopamine reuptake inhibitor
SNRI	selective serotonin reuptake inhibitor
SSRI	selective serotonin reuptake inhibitor
SUSAR	Unexpected serious adverse reaction
T1/2	half-life
TCA	tricyclic antidepressant
TdP	Torsades de pointes
THC	tetrahydrocannabidiol
TIA	transient ischemic attack
Tmax	time of peak plasma concentration
ULN	Upper limit of normal
VAS	Visual analog scale
WHO	World Health Organization
WOCBP	women of childbearing potential
β-HCG	beta human chorionic gonadotropin

3. INTRODUCTION

3.1. Disease Background

Attention-deficit/hyperactivity disorder (ADHD) is a chronic neuropsychiatric disorder, affecting about 5% of adults (Kessler et al., 2006). The disease is defined in Diagnostic and Statistical Manual of Mental Disorders, 5th ed. (DSM-5) by symptoms of inattention, hyperactivity, and impulsivity, resulting in significant impairment. When compared to the general public, individuals with adult ADHD have increased rates of psychiatric comorbidities, underemployment and unemployment, substance abuse disorders, divorce and marital separation, and motor vehicle accidents (Biederman et al., 2006a; Biederman et al., 2006b; Eakin et al., 2004; Wilens et al., 2004). Nonetheless, adult ADHD remains significantly under-diagnosed and under-treated, at rates of only 10%-25% affected adults receiving a proper diagnosis and less than 25% receiving proper treatment (Castle et al., 2007; Kessler et al., 2005; Kessler et al., 2006).
Medical management of ADHD entails the use of stimulants - methylphenidate (Ritalin), dextroamphetamine (Dexedrine), methamphetamine (Desoxyn), as well as an amphetamine-dextroamphetamine combination (Adderall). Several subjects experience adverse effects that can result in discontinuation (Gajria et al., 2014), some subjects are reluctant to take medication (Matheson et al., 2013), or respond insufficiently to stimulants and experience residual symptoms (Wigal, 2009), and long-term beneficial effects have not been convincingly established (Moriyama et al., 2013). Therefore, new medications are needed to treat ADHD.

3.2. Drug Background

Lysergic acid diethylamide (LSD) is a prototypical classic hallucinogen (Nichols, 2004; Passie et al., 2008) which was first synthesized by the Sandoz chemist Albert Hofmann who also discovered its psychotropic effects (Hofmann, 1979). In the 1950s to 1970s, LSD was initially used as an experimental tool ("psychotomimetic") to study psychotic-like states and model psychosis (Bercel et al., 1956; Koelle, 1958), and as an adjunct in
"psycholytic (substance-assisted) psychotherapy". LSD became one of the best studied substances with several thousands of early scientific reports (Hintzen et al., 2010; Nichols, 2004; Nichols, 2016; Passie et al., 2008). LSD has also been investigated for the treatment of alcoholism (Krebs et al., 2012), addiction (Savage et al., 1973), anxiety associated with terminal illness (Gasser, 2012b; Gasser et al., 2014; Grof et al., 1973; Pahnke et al., 1969), depression (Passie et al., 2008) and different manifestations of headache (Schindler et al., 2015; Sewell et al., 2006). Clinical research with LSD ended in the 1970s due to regulatory restrictions but its use for personal and recreational purposes continued. It is estimated that 38 million US people or 15% over the age of 12 years ingested a hallucinogen once in their lifetime (Johnston et al., 2016; Krebs et al., 2013a). In Europe, the lifetime prevalence of LSD use among young adults is estimated to be in the range of 6-8% (EMCDDA, 2016). Thus, a significant proportion of the western society is familiar with the effects of this substance. LSD is not associated with compulsive drug seeking (addiction), and there are relatively few medical emergencies and adverse effect (Nichols, 2016). Use of LSD or psilocybin is not associated with mental health problems and may even be protective (Johansen et al., 2015; Krebs et al., 2013b). In a safe setting, no drug-related severe adverse effects have been observed after administration of LSD in healthy subjects and subjects (Carhart-Harris et al., 2015; Dolder et al., 2016; Gasser, 2012b; Gasser et al., 2014; Schmid et al., 2015). The main adverse effects are due to the psychotropic effects of LSD at high doses (particularly 70 µg) when used in illicit and non-medical environment settings. Recently, the medical value of hallucinogens has again been studied in several clinical trials (Baumeister et al., 2014; Bogenschutz et al., 2015; Davenport, 2016; Gasser et al., 2014; Gasser et al., 2015; Grob et al., 2011; Johnson et al., 2014; Kupferschmidt, 2014; Nichols, 2016).

3.3. Rationale for Investigating Repeated Low-dose LSD Administration in Subjects with ADHD

There is a growing interest in the use of psychedelic substances for health-related purposes, including symptom relief for disorders like anxiety, depression, and pain (Nichols et al., 2017). Although recent clinical trials have focused on the use of experiential doses of these substances, anecdotal evidence supports the potential therapeutic utility of lower doses of psychedelic substances in reducing symptomatology of a range of mental and physiological disorders (Anderson et al., 2019a; Anderson et al., 2019b; Fadiman et al., 2019; Hutten et al., 2019a; Hutten et al., 2019b; Lea et al., 2020; Passie, 2019; Polito et al., 2019; Prochazkova et al., 2018). The

internet has a number of surveys, chat rooms and coaches touting the benefits of psychedelics, particularly LSD at sub-perceptual doses to treat ADHD, anxiety, and depression. It has yet to be shown whether a psychedelic experience as induced by a “full” regular dose is necessary to produce symptom relief, or whether (repeated) sub-perceptual doses have therapeutic potential as well.

Recently repeated low dosing, the practice of repeatedly using sub-experiential doses of psychedelics like LSD and psilocybin (Kuypers et al., 2019; Passie, 2019), has gained considerable media attention, where it is portrayed as a performance enhancing activity (Hutten et al., 2019b), and as a treatment for certain diseases like depression, anxiety and ADHD. In contrast to a typical recreational dose (approximately 100 µg) that is characterized by significant perceptual changes, a repeated low dose (approximately 10 to 20 µg) does not induce perceptual alterations such as visual distortion or synesthesia (Bershad et al., 2019; Family et al., 2020; Kuypers et al., 2019; Passie, 2019). The most widely suggested practice for repeated low dosing is taking one-tenth of a regular, recreational dose of a psychedelic once every three days (Fadiman et al., 2019; Kuypers et al., 2019; Passie, 2019).

Clinical evidence from controlled studies regarding the efficacy of repeated low dosing psychedelics for symptomatic relief is lacking. However, recent surveys have suggested that there are potential benefits of repeated low dosing LSD. Specifically, repeated low dosing psychedelics was rated more effective than conventional therapies for the treatment of ADHD by persons using psychedelics (Hutten et al., 2019b). Users mostly report repeated low dosing psychedelics for performance enhancement (Hutten et al., 2019a; Lea et al., 2019; Lea et al., 2020) and to improve mental health (Lea et al., 2019; Lea et al., 2020). Other reasons are mood enhancement and symptom relief, curiosity, and enhancing empathy (Hutten et al., 2019a). Taken together, that survey data indicate that people low dose as self-medication therapy for mental health as alternative or adjunct to conventional therapy (Lea et al., 2020). Adverse effects of low dosing include stronger-than-expected psychedelic effects, anxiety, and physical adverse effects (Lea et al., 2020). Other perceived limitations include issues related to dosing, taking illegal substances, limited or no mental health or cognitive improvement, unpleasant “off” days, only short-term benefits, and concerns about dependence and drug-related risks (Lea et al., 2019). Large other nonpublished survey data also indicate that people low dose to enhance mood, creativity, focus, and sociability. There are no controlled data on either the efficacy or adverse effects of repeated low dosing psychedelics, including LSD.

Therefore, the present study aims to investigate the effectiveness of repeated low dosing with LSD for treatment of ADHD in line with a common anecdotal practice. There are no scientific data to inform a possible mechanism of action for how LSD may improve ADHD symptoms. The rationale to test LSD in ADHD subjects is based on anecdotal evidence of efficacy. The same dose will then be administered twice weekly and effects on symptoms of ADHD in adults will be compared with placebo in a double-blind manner over 6 weeks.

3.4. Study Drug

3.4.1. Pharmacology of LSD

LSD is a very potent partial 5-HT2A receptor agonist (Nichols, 2016; Rickli et al., 2016). LSD also stimulates 5-HT1 receptors, adrenergic α1 receptors and dopaminergic D1-3 receptors (Rickli et al., 2015; Rickli et al., 2016). However, these receptor interactions are considered less relevant for the psychotropic action of LSD (Nichols, 2016; Preller et al., 2017) or other psychedelics. The subjective effects of psychedelics are generally considered to be mediated primarily by activation of the 5-HT2A receptor (Kraehenmann et al., 2017b; Preller et al., 2017; Vollenweider et al., 1998; Nichols, 2016).

3.4.2. Clinical pharmacology: Pharmacokinetics of LSD

LSD is well-absorbed after oral administration with an estimated oral bioavailability of 70100%. LSD is metabolized to the inactive metabolite O-H-LSD (Dolder et al., 2017b; Holze et al., 2019; Luethi et al., 2019; Steuer et al., 2017). There are no active metabolites of LSD reaching relevant plasma concentration to be active in vivo (Dolder et al., 2017b; Holze et al., 2019; Luethi et al., 2019; Steuer et al., 2017). After acute administration of LSD, peak plasma concentration of LSD is reached at 1-3 h. The plasma elimination half- life of LSD is approximately 3 h (Dolder et al., 2017b; Holze et al., 2019, Holze et al., 2021b). At doses of LSD of 100-200 µg, LSD is almost completely eliminated from the body within 12-24 h

(Dolder et al., 2017b; Holze et al., 2019). 24 hours after the administration LSD up to 200 µg, no LSD could be detected in the urine (Dolder et al., 2015a; Dolder et al., 2015b). The pharmacokinetics of low doses of 5-20 µg LSD have also been investigated in 20 healthy subjects (Holze et al., 2021a) . After administration of 20 µg of LSD (the dose to be used in the present study), peak plasma concentrations were reached at (geometric mean and range) 1.1 h (0.2-2.4). The elimination half-life was 2.9 h (1.6-2.4).

3.4.3. Clinical pharmacology: Pharmacodynamics - psychological and physiological effects of LSD

LSD produces dose-dependent marked acute subjective (psychedelic) effects and weak autonomic system stimulation. Subjective effects parallel the LSD plasma concentrationtime curves. The acute pharmacodynamic effects of LSD dose-dependently peak at 2 to 3 h and gradually subside, lasting up to 9 and 12 h after administration of high doses of 100 and 200 µg, respectively (Dolder et al., 2015b; Dolder et al., 2016; Dolder et al., 2017b; Holze et al., 2019; Schmid et al., 2015). The pharmacodynamics of low doses of 5-20 µg LSD have also been investigated (Holze et al., 2021a). The dose of 20 µg, which is the dose to be used in the present study, produced subjective drug effects starting after 0.7 h (0.041.3), reaching a maximum effect at 2.3 h (1.2-4.6), and lasting for 6 h. The maximum subjective response (any subjective drug effect) reached 3.6 on a scale from 0-10.
Alterations of the mind as measured as subjectively rated on the 5D-ASC scale reached blissful state scores of 5-10%, and anxiety scores of < 3% of the scale maxima, indicating very weak acute effects of LSD at this dose (Holze et al., 2021a). A dose of 20 µg LSD base did not relevantly change blood pressure or heart rate (Holze et al., 2021a). Other placebo-controlled studies in healthy subjects similarly reported weak acute drug effects of LSD at low doses. A study using single doses of 5-20 µg of LSD tartrate reported a statistically significant and small subjective drug effect (Yanakieva et al., 2019). Low doses of LSD of 5-20 µg tartrate did not significantly alter self-ratings of perceptual distortion and subjective concentration, but there were trends toward impairments (Yanakieva et al., 2019). A study using LSD tartrate in doses of 6.5-26 µg reported acute increases in ratings of feel drug, like drug, vigor and produced no alteration of cognition of physiological measures (Bershad et al., 2019). On the 5D-ASC, the 26 µg LSD dose increased ratings of blissful state and anxiety to approximately 15 and 3% (maximum 100%), respectively (Bershad et al., 2019). Furthermore, effects of 25 µg LSD were assessed in 16 healthy subjects. A dose of 25 µg of LSD produced weak subjective drug effects (ratings of approximately 15% on a 0-100% scale) that were significantly different from placebo. Mean peak responses were 32% and 2% for good and bad drug effects, respectively. On the 5D-ASC, mean ratings of blissful state and anxiety were 6 and 0% (scale maximum: 100%), respectively. The mean effect duration was 7 h (data on file). The dose was distinguished from placebo by most participants. In all the studies discussed above there were no relevant subjective bad drug effects and no relevant adverse reactions. Taken together, available evidence from similar controlled studies suggests that LSD at a dose of

20 µg will produce potentially noticeable but very weak acute positive subjective effects lasting for approximately 6 hours. While LSD moderately increases blood pressure, heart rate and body temperature at high doses (Dolder et al., 2017a; Dolder et al., 2017b; Holze et al., 2019; Holze et al., 2020; Schmid et al., 2015), no clinically relevant cardiostimulant effects are expected at a dose of 20 µg (Holze et al., 2021a).

3.5. Recent and Current Clinical Studies with LSD in Healthy Subjects and Subjects

LSD was extensively investigated in humans in the 1950s and 1960s. Clinical research with LSD ended in the 1970s due to regulatory restrictions, but its use for personal and recreational purposes continued. In recent years, there has been a renewed interest in the research of hallucinogens under laboratory conditions (Carhart-Harris et al., 2015; Dolder et al., 2016; Liechti, 2017; Nichols, 2004; Nichols, 2016; Passie et al., 2014; Schmid et al., 2015; Terhune et al., 2016), and the use of hallucinogenic drugs such as psilocybin and LSD as treatment for depression (Carhart-Harris et al., 2017; Carhart-Harris et al., 2016a), anxiety associated with life-threatening diseases (Gasser, 2012b; Gasser et al., 2014), and substance disorders (Johnson et al., 2016; Krebs et al., 2012). LSD is currently being investigated in several research groups in healthy subjects and subjects. Most of the recent research on LSD was conducted or is being conducted by Liechti et al. at the University Hospital Basel (Dolder et al., 2018; Dolder et al., 2016; Holze et al., 2019; Holze et al., 2020; Liechti, 2017; Liechti et al., 2017; Luethi et al., 2019; Mueller et al.,

2017a; Mueller et al., 2017b; Muller et al., 2018; Schmid et al., 2015; Schmid et al., 2018; Schmidt et al., 2017; Strajhar et al., 2016). Additionally, a study using singly dose administration of small doses of LSD, similar to the present study but in healthy volunteers, has been completed by Kuypers et al. at the University of Maastricht (Holze et al., 2021a; Hutten et al., 2020a; Hutten et al., 2020b; Ramaekers et al., 2020), and a study using multiple dose administration of small doses is ongoing. The single dose administration study documented the pharmacokinetics and effect-profile for microdoses of LSD (Holze et al., 2021a; Hutten et al., 2020b), an increase in circulating brain-derived neurotrophic factor (BDNF) (Hutten et al., 2020a), and found beneficial effects on experimentally induced pain (Ramaekers et al., 2020). Furthermore, clinical trials are currently being conducted in Switzerland in subjects with anxiety disorder, major depression, and cluster headache using the same product to be used in the present study, but at higher doses (clinicaltrials.gov: NCT03781128, NCT03866252, and NCT03153579).

The University Hospital Basel completed several double-blind, placebo-controlled, random-order cross-over Phase 1 studies in healthy subjects. The first study (clinicaltrials.gov: NCT01878942) used a dose of 200 µg oral LSD in 16 subjects (8 male, 8 female), and characterized the psychological, physiological, endocrine, and pharmacokinetic effects of LSD (Dolder et al., 2015b; Dolder et al., 2016; Schmid et al., 2015; Strajhar et al., 2016). Administration of LSD produced pronounced alterations in waking consciousness that lasted up to 12 hours. The predominant effects induced by LSD included visual hallucinations, audiovisual synesthesia, and positively experienced derealization and depersonalization phenomena. Subjective well-being, happiness, closeness to others, openness, and trust were increased by LSD. LSD also increased blood pressure, heart rate, and body temperature. Adverse effects produced by LSD completely subsided within 72 hours, and no severe acute adverse effects were observed (Schmid et al., 2015). No sex differences could be shown. The acute subjective and sympathomimetic responses to LSD were closely associated with the concentrations in plasma over time, and exhibited no acute tolerance (Dolder et al., 2015b). In the second study (clinicaltrials.gov: NCT02308969), effects of a single dose of LSD of 100 µg on brain activation (fMRI) were investigated in 24 healthy subjects (Dolder et al., 2016; Mueller et al., 2017a; Mueller et al., 2017b; Schmidt et al., 2017). At the 100 µg dose, LSD induced lower ratings for blissful state, insightfulness, and changed meaning of percepts compared with the 200 µg dose. LSD also produced lower visual analog scale (VAS) ratings of good drug effects, closeness to others, openness, trust, bad drug effects and anxiety than the higher dose (Dolder et al., 2016). Changes in vital signs such as blood pressure, heart rate, body temperature, and pupil size or acute adverse effects were all similar and relatively moderate at the 100 and the 200 µg dose. Taken together, the lower 100 µg dose of LSD did not produce a similarly strong LSD response with regards to the subjective effects. LSD decreased amygdala reactivity to fearful stimuli (Mueller et al., 2017a), enhanced thalomocortical functional connectivity (Mueller et al., 2017b), and enhanced connectivity between normally more functionally separated resting state networks (Muller et al., 2018). LSD also produced longlasting effects (Carhart-Harris et al., 2016b; Schmid et al., 2018). LSD increased optimism and trait openness at two weeks compared with placebo in a study conducted at Imperial College London (Carhart-Harris et al., 2016b). One year after LSD administration, significant and persisting increases in positive attitudes, positive mood changes, positive social effects, positive behavioral changes, and in well-being/life satisfaction could be observed, which were subjectively attributed to the LSD experience (Schmid et al., 2018). No changes in negative attitudes, negative mood, antisocial/negative social effects, or negative behavior were attributed to the LSD experience (Schmid et al., 2018). None of the participants reported any psychological problems or perceptual changes (e.g., flashbacks) up to 1 month after the LSD session, and no adverse effects were reported spontaneously up to 12 months following LSD administration (Schmid et al., 2018). 5D-ASC total scores, reflecting acutely induced alterations in consciousness, and MEQ total scores correlated with changes in well-being/life satisfaction 12 months after LSD administration (Schmid et al., 2018). The data indicate that in healthy subjects, psychedelics increased openness and psychological well-being mid- to long-term, possibly related to the hallucinogen induced profound alterations of consciousness and mystical-type experiences (Carhart Harris et al., 2016b; Griffiths et al., 2008; MacLean et al., 2011; Schmid et al., 2018). Consistently, there is no evidence of psychological or psychiatric problems associated with the use of psychedelic substances by healthy subjects according to follow-up data from placebo- controlled studies (Carhart-Harris et al., 2016b; Schmid et al., 2018; Studerus et al., 2011) or epidemiological data (Johansen et al., 2015; Krebs et al., 2013b).

A recently completed study (Holze et al., 2021b) assessed the role of the 5-HT2A-receptor in LSD-induced altered states of consciousness (EKNZ 2017-01348), using different doses of LSD (25,

50, 100, and 200 µg) with a pre-treatment with the 5-HT2A-receptor antagonist ketanserin for the LSD 200 µg dose. Ketanserin pre-treatment reduced the response to 200 µg LSD to that of 25 µg of LSD alone indicating a key role of the 5-HT2A-receptor in the mediation of the acute psychedelic effects of LSD, and confirming a previous study conducted in Zurich Switzerland (Preller et al., 2018; Preller et al., 2017; Preller et al., 2019). The study also described the dose-response of LSD in the 25-200 µg dose range (Holze et al., 2021b). Furthermore, a recently published study compared the acute effects of LSD (100 µg) with those of other psychoactive substances such as the prototypical stimulant D-amphetamine and the prototypical entactogen/empathogen MDMA (Holze et al., 2020). LSD induced significantly higher ratings on the 5D-ASC and MEQ scale than MDMA and D-amphetamine. LSD also produced greater subjective drug effects, ego dissolution, introversion, emotional excitation, anxiety, and inactivity than MDMA and Damphetamine. It also induced greater impairments in concentration, sense of time, and speed of thinking compared with MDMA and D-amphetamine (Holze et al., 2020).
In collaboration with psychiatrists, an ongoing study investigated effects of LSD-assisted psychotherapy in subjects suffering from anxiety symptoms in severe diseases or in psychiatric anxiety disorders (EKNZ 2016- 00992, clinicaltrials.gov: NCT03153579). A clinical Phase 2, double-blind, placebo-controlled pilot study previously compared LSD- and placebo-assisted psychotherapy in subjects suffering from anxiety associated with advanced-stage life- threatening diseases (Gasser et al., 2014; Gasser et al., 2015). In this study, twelve subjects were included and eight received 200 µg of LSD twice in two sessions 2-3 weeks apart, and three subjects received placebo (a low dose of LSD of 20 µg). The study documented a significant decrease in anxiety 2 months after the two LSD sessions compared with baseline anxiety scores, whereas State-Trait Anxiety Inventory (STAI) scores did not decrease after the placebo sessions. The study also documented sustained decreases in anxiety up to 12 months after the LSD treatments (Gasser et al., 2015). Additionally, LSD markedly reduced the number of migraine attacks in two migraine subjects in this study (Gasser et al., 2014). There were no drug-related severe adverse effects, no panic reactions, or other medical or psychiatric complications. As a consequence of this pilot study in subjects and previous evidence, the use of LSD in combination with psychotherapy was re-started (after similar activity in the 1980-90s (Gasser, 1996)) in Switzerland with the authorization of the Swiss Federal Office of Public
Health (BAG). Currently, LSD is being used in selected subjects (“compassionate use”) in Switzerland, mainly in those with anxiety, depression, compulsive disorder, cluster headache, and migraine (Schmid et al., 2021).
In Zurich, Vollenweider and colleagues conducted a double-blind, placebo-controlled, cross-over study using a dose of 100 µg oral LSD in 25 healthy participants (Kraehenmann et al., 2017a; Kraehenmann et al., 2017b; Preller et al., 2017). The study included two administrations of LSD in the same participant, 100 µg LSD alone, and 100 µg LSD combined with ketanserin (40 µg orally). All effects of LSD were blocked by ketanserin (Kraehenmann et al., 2017a; Kraehenmann et al., 2017b; Preller et al., 2017) indicating a mediating role for the 5-HT2A- receptor, as previously shown for psilocybin (Vollenweider et al., 1998).
In London, Carhart-Harris and colleagues performed an experimental single-blind, withinsubject, placebo-controlled pilot study in 10 healthy volunteers using 40-80 µg LSD administered intravenously (Carhart- Harris et al., 2015; Kaelen et al., 2015). The same group also conducted another larger placebo-controlled cross-over study in 20 subjects, using a dose of 75 µg LSD (intravenously, corresponding to about 100 µg oral LSD) (Carhart-Harris et al., 2016b; Carhart-Harris et al., 2016c; Kaelen et al., 2016; Lebedev et al., 2016; Roseman et al., 2016; Speth et al., 2016; Tagliazucchi et al., 2016). LSD produced heightened mood, a blissful state, and also acute psychosis-like symptoms including thought disorder, delusional thinking and paranoia. There was only a small increase in anxiety, significantly smaller than the blissful experience. Overall, there was a bias towards positive affect. At two weeks, there was no effect on delusional thinking, and a trend towards less distress and less preoccupation with delusional thoughts (Carhart-Harris et al., 2016b). These recent experimental studies are expected to provide insight into the mechanisms of action of psychedelics that may contribute to their therapeutic potential (Liechti, 2017).
Eleusis Benefit Corporation, in collaboration with researchers in the UK, conducted a clinical trial in older healthy adults aged 55-75 years using small single doses of LSD of 520 µg that were repeatedly administered every fourth day over a 21-day period. LSD did not produce relevant alterations of perception or concentration. LSD was well tolerated, and the frequency of adverse events was no higher than for placebo. Assessments of cognition, balance, and proprioception revealed no impairment (Family et al., 2020; Yanakieva et al., 2019). In terms of dosing schedule, this published study (Family et al., 2020; Yanakieva et al., 2019) and a multiple dose

administration study currently conducted in Maastricht are the most similar to the present one. Both studies support the safety of the current dosing approach.

4. STUDY OBJECTIVES

4.1. Primary Objective

To assess the treatment efficacy vs placebo of repeated low doses (20 µg) of MM-120 for six weeks in adult subjects with ADHD measured by Adult Attention Deficit Investigator Symptom Rating Scale (AISRS).

4.2. Secondary Objectives

- To assess treatment efficacy vs placebo measured by change from baseline in AISRS after 1 week (2 doses) of treatment.
- To assess treatment efficacy vs placebo based on the proportion of subjects who experience at least a 1-point decrease in the Clinical Global Impression - Severity of Illness Scale (CGI-S).
- To assess treatment efficacy vs placebo measured by change from baseline in CGI-S.
- To assess other efficacy endpoints by the Adult ADHD Self-Report Scale (ASRS) and Connors' Adult ADHD Rating Scale
- To assess the pharmacokinetic (PK) profile of MM-120 in subjects with ADHD.
- To explore the pharmacokinetic/pharmacodynamic (PK/PD) relationship of single doses of MM-120 and psychological and physiological effects.
- To assess the safety and tolerability by AE and SAE assessment

4.3. Exploratory Objective

To explore sleep quality/ duration as recorded in the daily diary. For the purpose of future exploratory analysis (not covered in this SAP), for subjects who consent to provide a pharmacogenomics (PGX) sample, a single blood sample (2.5 mL) in a proprietary integrated standardized system for collection and stabilization of specimens (bio banking) will be collected during the study and stored.

5. STUDY PLAN AND PROCEDURES

5.1. Overall Study Design

This study is a multi-center, randomized, double-blind, placebo-controlled Phase 2a study of low dose MM-120 (20 µg) compared with a placebo administered for 6 weeks (twice a week).
There will be a 1:1 randomization, double-blind, to MM-120 or placebo of 52 adults with ADHD with the aim to reach 26 evaluable subjects in each of the 2 arms at Week 6.
There will be 2 arms:

- Arm 1-Placebo: a total of 26 subjects will receive a placebo identical in appearance to the IMP administered orally twice weekly for 6 weeks. Subjects and site staff will remain blinded to the treatment arm.
- Arm 2-MM-120: a total of 26 subjects will receive 20 µg of MM-120 twice weekly for 6 weeks. Subjects and site staff will remain blinded to the treatment arm.

Subjects will be evaluated at Baseline, Week 1, Week 6, and Week 10 (follow-up) for AISRS and CGIS by a trained study personnel; the primary endpoint will be based on evaluation of AISRS at Baseline and at Week 6, with a key secondary endpoint after 1 week of treatment.
Subjects are free to discontinue the study at any time without providing a reason. However, the Investigator should try to identify the reason and document in the eCRF.
Specific reasons for discontinuing from the study should be documented in the eCRF, and include, but are not limited to:

- Subject's decision
- The Investigator or Sponsor judges that it is in the best interest of the subject to stop study treatment due to any abnormality compromising subject safety
- Pregnancy
- Severe noncompliance to the study protocol as judged by the Investigator and/or the Sponsor.

Subjects who prematurely discontinue may be replaced, per Sponsor decision. Subjects will not be allowed to re-enter the study.
The study will stop enrolling subjects once 26 subjects per arm are randomized, or if the Investigator or the Sponsor judges that it is in the best interest of the subjects to stop study enrollment due to low accrual or safety or other reason. Subjects who have given informed consent and have not yet been excluded, completed, or terminated from the study will be allowed to continue in the study. An evaluable subject is defined as a randomized subject (as defined in the Statistical Analysis Plan [SAP]).

5.1.1. Screening and Baseline visits

Potential study subjects who provide informed consent will have eligibility evaluated/confirmed at 2 visits: 1) Screening, and 2) Baseline. The screening visit occurs up to 4 weeks prior to Baseline. An assessment will be made to confirm or make the ADHD diagnosis with the Mini International Neuropsychiatric Interview (MINI). The subject must have an AISRS score of ≥ 26 and a CGI-S of≥ 4 at screening and must meet all other eligibility criteria. The baseline visit will occur on the clinic day before Day 1 and will confirm AISRS score. If there is a ≥ 13-point change in the AISRS between the screening visit assessment and the baseline assessment, or if the subject does not return for the baseline AISRS, the subject will not be randomized, and will be terminated from the study. Eligible subjects will be randomized at Day 1 to either 20 µg of MM-120 or to matching placebo and begin the double-blind treatment period on Day 1.

Test Days

The double-blind treatment period will be 6 weeks in duration, with dosing twice weekly. Day 1 will be the first day of dosing. At the end of 6 weeks, subjects will be assessed by a study physician. All subjects and site staff will remain blinded to the treatment arm.

5.1.2. Study Schedule Diagram

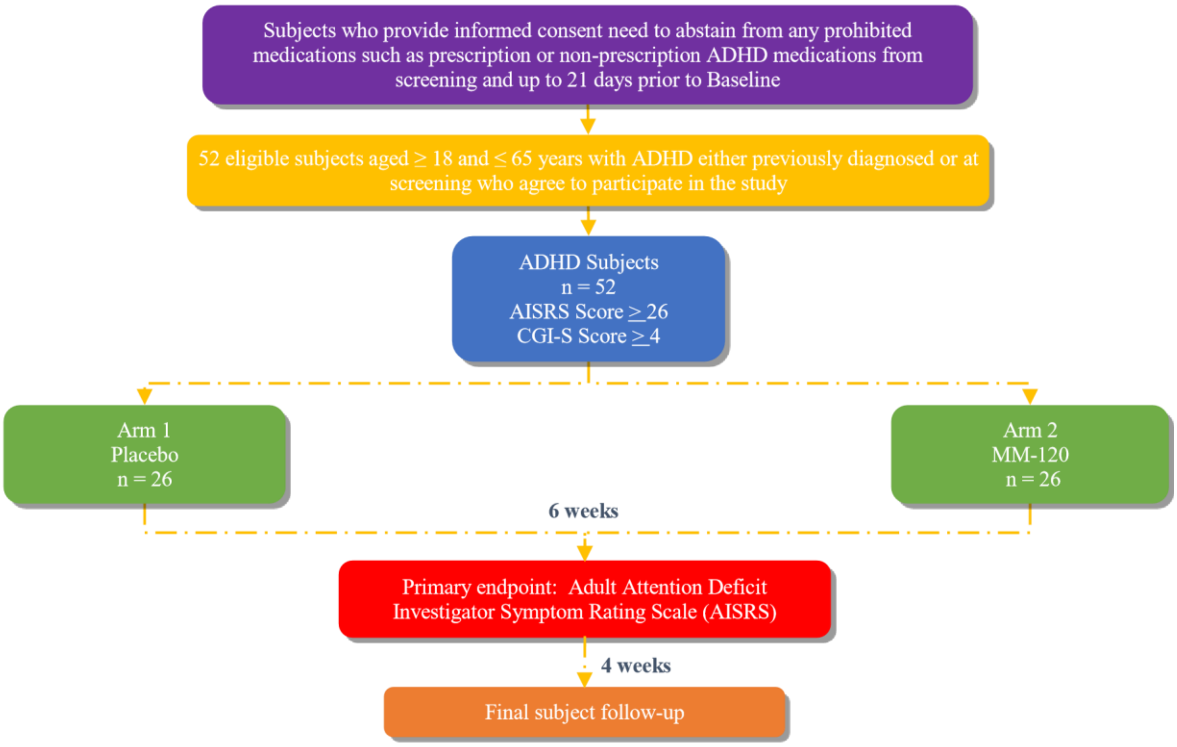

5.2. Endpoints

5.2.1. Primary Endpoints

The primary endpoint is the mean change from baseline in ADHD symptoms, as assessed by the AISRS after 6 weeks of treatment.

The AISRS total score consists of 18 items from the original ADHD-RS, which were derived based on DSM-5 criteria for ADHD. The ADHD-RS includes 9 items that address symptoms of inattention, and 9 items that address symptoms of impulsivity and hyperactivity. Each item is rated

from 0 to 3. The AISRS total score can range from 0 to 54. A higher score corresponds to a worse severity of ADHD.

5.2.2. Secondary Endpoints

ADHD related endpoints

- Key secondary endpoint: change from baseline in AISRS after 1 week (2 doses) of treatment
- Occurrence of subjects who experience at least a 1-point decrease in the CGI-S
- Change from baseline in CGI-S after 1 week and 6 weeks of treatment.
- Change from baseline in subject self-assessment by the Adult ADHD Self-Report Scale (ASRS) and Connors' Adult ADHD Rating Scale (CAARS).

Safety endpoints

- Vital signs (supine blood pressure, body temperature, heart rate, respiratory rate)
- 12-lead safety ECG
- Adverse events (e.g., psychological and/or physiological adverse events)
- Columbia-Suicide Severity Rating Scale (C-SSRS)
- Safety laboratory evaluation and Urine pregnancy testing

Pharmacokinetic endpoints

- Blood samples for PK evaluation will be collected for 6 hours after the administration of the IMP on day 1.
- Cmax
- Tmax
- AUC
- T1/2

Acute effects (Pharmacodynamic) endpoints

- 5 Dimensions of Altered States of Consciousness scale (5D-ASC),
- Mystical Experience Questionnaire (MEQ), drug effect Visual Analog Scale (VAS)

5.2.3. Exploratory Endpoints

- Sleep quality/ duration rating will be evaluated based on daily diary entries and details of the analysis will be included in the SAP.

6. SUBJECT SELECTION CRITERIA

The Investigator should keep a record (i.e., screening log) of subjects who were screened. Each subject must meet all the inclusion criteria and none of the exclusion criteria for this study at the time of randomization. Under no circumstances can there be exceptions to this rule. A repeat test may be allowed in the screening period with the Sponsor's medical monitor approval, with the exception of the AISRS and CGI-S, which can only be assessed once at screening for eligibility purpose.

6.1. Inclusion Criteria

All inclusion criteria will be based on the medical judgment of the Investigator. For inclusion in the study, subjects must meet all the following criteria:

1. Ability and willingness to provide written, informed consent prior to initiation of any study-related procedures and to adhere to all study requirements.
   NOTE: The subject (i.e., not a legally authorized representative) must be cognitively able to understand the requirements of the study and provide the informed consent.
2. Age ≥ 18 and ≤ 65 years at Screening.
3. Subjects with the diagnosis of Diagnostic and Statistical Manual of Mental Disorders5 (DSM-5) ADHD, as determined by clinical evaluation and confirmed by structured interview (MINI).
4. AISRS total score of ≥26 at screening.
5. CGI-S score of ≥4 at screening.
6. Must be willing to receive IMP dose twice weekly. On Day 1, the subject will come to the clinic and must be willing to take a taxi or public transportation home or be accompanied by a caregiver and not drive a car, use heavy equipment, or participate in any other dangerous

activity for the remainder of the day after receiving IMP (NOTE: at any protocol visit after Day 1 dosing, dosing visits may occur at the subject's home at the discretion of the PI, conducted by one of the study investigators or delegate and administered under supervision followed by the performance of the same procedures done at the clinic including safety monitoring. If early withdrawal is considered due to any safety issue identified, the Sponsor's medical monitor should be notified. If a remote visit is conducted due to any reason related to the COVID-19 pandemic, notification must be sent to the Medical Monitor's dedicated email address and Urgent Safety Measures as outlined in this protocol must be followed.)

7. Must be willing to refrain from more than 6 standard alcoholic drinks per week (1 standard drink corresponds to 0.1 L wine, 0.3 L beer, or 4 cL liquor), more than 10 cigarettes a day, and more than 2 cups of coffee a day throughout the study treatment period (6 weeks) and until the last study visit is complete (EoS or ET).

6.2. Exclusion Criteria

Subjects should not be included in the study if any of the following exclusion criteria are met:

1. Past or present diagnosis of a primary psychotic disorder or first-degree relative with a psychotic disorder.
2. Past or present bipolar disorder (DSM-5).
3. Other current psychiatric disorders that, in the opinion of the Investigator or medical supervisor, may confound the results of the study (e.g., obsessive-compulsive disorder, dysthymic disorder, panic disorder, dissociative disorder, anorexia nervosa or bulimia nervosa).
4. Subjects with past (> 1 month prior to the screening visit) or present substance use disorder (except nicotine, provided subject does not smoke more than 10 cigarettes a day).
5. Somatic disorders including Central Nervous System (CNS) involvement of cancer, severe cardiovascular disease, untreated hypertension, severe liver disease (liver enzyme increase by more than 3x the upper limit of normal except unconjugated hyperbilirubinemia due to Gilbert's Disease, per Investigator), severely impaired renal function (estimated creatinine clearance < 50 mL/min by CKD-EPI formula), or anything else that, in the judgment of the Investigator or medical supervisor, poses too great a potential for side effects.
6. Any lifetime history of suicide attempt; or recent (within 6 months prior to the screening visit) active suicidal thoughts or ideation (defined as a suicidal ideation score of 2 or greater in the Columbia-Suicide Severity Rating Scale [C-SSRS]); or endorsement of any suicidal behavior on the C-SSRS within the past 6 months prior to the screening visit.
7. Likely to require psychiatric hospitalization during the course of the study.
8. Once consent is signed, subject not willing or able to stop any prescription or nonprescription ADHD medications during screening and prior to the baseline visit through final study visit (EoS or ET). *A list of prohibited medications is provided in Appendix 1.*
9. Plan to start, stop, or alter the use of any medications, supplements, or other therapeutics from Baseline until EoS or ET (see Appendix 1 for list of prohibited medications).
10. Plan to start, stop or alter the use of psychotherapy, massage, meditation, acupuncture, hypnosis, yoga, or other similar therapy/activity from the time of providing informed consent until EoS or ET.
11. Use of potent CYP2D6 inhibitors; moderate CYP2D6 inhibitors by Investigator discretion (see Section 5.5.1.1 and Appendix 3).
12. Likely to need use of any psychiatric medications with the potential to confound interpretation of study results or impact safety, at the discretion of the Investigator, in the 10 weeks following Baseline up to EoS or ET (see Appendix 1 for list of prohibited medications).
13. Use of investigational medication/treatment in the past 30 days prior to the screening visit.
14. Subjects with a positive urine drug screen (with the exception of THC or metabolites) at Screening OR Baseline.
15. Clinically significant abnormal baseline laboratory values, VSs, and ECG that include the following:
    a. Have evidence of clinically significant hepatic disorder (e.g., alanine aminotransferase [ALT] or aspartate aminotransferase [AST] > 3X ULN (except for Gilbert's disease), and

b. Any clinically significant abnormal metabolic or hematologic screen, per Investigator or medical supervisor decision

c. Exclusionary blood pressure: >140 mm Hg (systolic) or >90 mm Hg (diastolic); heart rate <45 beats/minute or >90 beats/minute after an approximately 5minute supine or semi-supine rest

NOTE: If the first measurement of a subject's heart rate is > 90 beats/minute, a second recording is allowed after an additional approximately 5-minute supine rest

d. Exclusionary ECG parameters: QTcF > 450 msec (men), QTcF >470 msec (women)

e. Any clinically significant abnormal electrocardiogram (ECG) finding (e.g., uncontrolled atrial fibrillation, ischemia) at Screening (Visit 1) or Baseline (Visit 2), as determined by the Investigator or medical supervisor (in consultation with a cardiologist, if needed.

16. Any other condition, therapy, laboratory abnormality, or other circumstance that, in the opinion of the Investigator or medical supervisor, may pose additional risk to the subject from participation in the study, may interfere with the subject's ability to comply with study procedures, may make participation in the study not in the subject's best interest or may confound the results of the study.
17. Prior history or ongoing neuropsychiatric signs or symptoms associated with COVID19 such as development of, or current disorder, during or after a COVID-19 infection including anxiety, memory loss, confusion, depression, delirium, agitation, or psychosis.
18. Women of childbearing potential (WOCBP) (i.e., physiologically capable of becoming pregnant) who are unwilling or unable to use a highly effective method of contraception, as defined in Appendix 2, for the duration of the study, OR Men physiologically capable of fathering a child who are sexually active with WOCBP but are unwilling or unable to use barrier contraception (e.g., condom with or without spermicidal cream or jelly) for the duration of the study.

    NOTE: See Appendix 2 for definitions of WOCBP and highly effective methods of contraception and for information about unacceptable methods of contraception.
19. Women who are currently pregnant or breastfeeding or plan to become pregnant or breastfeed during the study.
20. Men who plan to donate sperm during the study.
21. Use of weight loss drugs within 21 days of screening until the end of study.
22. Subjects who are either unable or unwilling to consume alcohol in any amount (including due to religious or personal reasons).
23. Subjects who have a change in AISRS score of ≥13-points between screening and baseline visits.

7. STUDY CONDUCT

7.1. Subject Enrolment and Randomization

The Investigator will ensure:

- Signed informed consent is obtained from each potential subject before any study specific procedures are performed.
- The eligibility of each subject is determined, as described in Sections 4.1 and 4.2, before administering any IMP.

Each screened subject will be identifiable by a unique screening number. The randomization number will be assigned to the subject on Day 1 before first dosing.

This will be a double-blind study, with the random code generated by the study biostatistician, and provided to the GMP facility for packaging. The subject and Investigator will remain blinded to treatment assignment until the conclusion of the study unless it necessary to unblind the data for safety reasons. Only the GMP facility producing the IMP and the Principal Investigator (PI) at each site have access to the code (sealed envelopes). In case of a medical emergency, and in case of premature study termination, the code can be accessed by the PI.

In case of an emergency requiring immediate knowledge of the study treatment and appropriate management of the subject requires knowledge of treatment randomization, the treatment code

may be broken by the Investigator. The Investigator should discuss the case with the Sponsor's Medical Monitor immediately after breaking the treatment code.
The Sponsor's unblinded personnel may unblind treatment assignment for an individual subject's SAE in order to fulfill expedited regulatory requirements.
Changes that may be implemented, if needed, due to the coronavirus disease 2019 (COVID19) pandemic are described in Section 7.11 and a detailed impact will be included in the clinical study report (CSR).

7.2. Procedures for Handling Incorrectly Randomized Subjects

Subjects who fail to meet the inclusion/exclusion criteria should not, under any circumstances, be randomized into the study. There can be no exceptions to this rule.
A subject who does not meet the eligibility criteria but who is randomized in error, and is identified before dosing, should be withdrawn from the study and another subject should be enrolled and receive the same treatment.
Sponsor's Medical Monitor should be notified if an ineligible subject is dosed.

7.3. Treatments

It is the Investigator's responsibility to establish a system for handling the IMP, to ensure that:

- Deliveries of such products are correctly received by a responsible person.
- Deliveries are recorded and IMP is handled and stored safely and properly.
- IMP provided for this study will be used only as directed in the study protocol. The study personnel will account for all IMP received at the site, dispensed to the subject, and returned to the pharmacy/site. Any discrepancies should be documented, investigated, and appropriately resolved.

Any unused IMP are accounted for and returned to the IMP manufacturer (Basel), or destroyed by the hospital pharmacy of the research site. Receipt of the IMP and confirmation of destruction in writing will be obtained by each study site after study termination.

7.4. Identity of Investigational Medicinal Product

The drug substance is d-lysergic acid diethylamide (LSD). The identity of the IMP is presented below:

IMP	Dose
MM-120	LSD-tartrate 0.029mg (corresp. 0.020 mg LSD), ethanol 0.16 g, aqua pur ad 1 mL
Placebo	Ethanol 0.16 g aqua pur ad 1 mL

Substance preparation and quality control: Analytically LSD d-tartrate salt will be obtained from Onyx Scientific, United Kingdom. LSD will be formulated as an oral drinking solution in dark vials. A Swissmedic approved Good Manufacturing Practice (GMP) facility (Apotheke Dr. Hysek, Biel Switzerland) will prepare the IMP and the placebo (solution without MM-120 in identical vials), as well as perform the individualized packaging based on the randomization list, labeling and quality control (QC).

7.4.1. Packaging and labelling

The IMP will be packaged and labelled in accordance with current Good Manufacturing Practices (cGMPs) and provided to the sites. The shipping of the IMP to the sites will be in accordance with Good Distribution Practice (GDP). Re-supply will be executed as needed by the same manufacturer.
Labelling of the IMP will be performed in accordance with cGMPs and will comply with the applicable laws and regulations of the countries in which the study sites are located.
It is the responsibility of the Investigator to ensure that accurate accountability records are maintained throughout the study.

7.5. IMP, Concomitant, and Post-Study Treatment(s)

IMP will be administered twice a week, every 3 to 4 days. The IMP can be administered at the clinic on Day 1 or at home after Day 1 per PI decision under supervision of study personnel (i.e., the Investigator or delegate). Dosing/dispensing is based on the delegation log by authorized study personnel. The IMP cannot be provided to the subjects to take home. However, instead of administering the IMP at the clinic, IMP may also be brought to the subject's home by one of the study investigators or delegates on the delegation log and administered at home. A supportive person of the subject's choosing may be present with the subject in the home. Dosing at home is not allowed on Day 1 or if protocol procedures cannot be completed at the home visit or under urgent safety measures per Section 7.11. Furthermore, adverse effects and the use of concomitant medication are recorded based on the delegation log by authorized study personnel and the diary is checked; all safety assessments will be obtained and AEs recorded per Schedule of Events. Administration at home is mainly intended to facilitate trial conduct. If a remote visit is conducted due to any reason related to the COVID-19 pandemic, notification must be sent to the Medical Monitor's dedicated email address and Urgent Safety Measures as outlined in this protocol must be followed.

Apart from paracetamol/acetaminophen (not more than 3 g per day) if needed, no concomitant medication or therapy will be allowed that may interfere with the IMP (see Section 5.5.1). The subject should be instructed that no other medication is allowed, including herbal remedies, vitamins, mineral supplements, and over the counter products, without the consent of the Investigator.

The administration of all medication, (including the IMP), should be recorded in the appropriate sections of the eCRF. Treatment adherence will be assured by supervised administration of the IMP by the Investigator or delegate. The dose, time, and date of administration of the IMP will be recorded on the IMP accountability log and checked by the monitor at monitoring visits. Unused IMP should be destroyed at the site or returned to the manufacturer.

7.6. Prohibited concomitant medications

The use of medications, supplements, and other therapeutics (over the counter or prescribed) for treating attention-deficit/hyperactivity disorder (ADHD), anxiety, depression or mood disorders are prohibited from Day -1/Baseline (Visit 2) until End of Study or Early Termination (except if needed for care and treatment of the subject during the study per Investigator discretion). Additionally, the use of other substances, herbal treatments, etc. that may confound the results of the study are prohibited from Day 1/Baseline (Visit 2) until End of Study or Early Termination. Subjects must also avoid starting, stopping, or altering non-medicinal therapies/activities from the time of providing informed consent until End of Study or Early Termination. Any prohibited substance(s) being taken at the time of signing consent at screening (Visit 1) should be tapered prior to Day -1/Baseline (Visit 2), if safe for the subject, in the opinion of the Investigator; tapering will be done with oversight from the Investigator or medical supervisor and in accordance with information provided in Appendix 1. A listing of specific restrictions for concomitant therapy use during the course of this study was provided, with any necessary washout periods or allowable conditions described. The subject must meet all eligibility criteria for which prohibited medication was prescribed, including but not limited to, psychotic disorder, bipolar disorder, or major depressive disorder with psychotic features OR any disorder that puts a subject at risk for a challenging experience, e.g., panic disorder.

Subjects will be tested for cannabis (THC metabolite) but will not be excluded in case of a positive test. Subjects will be strongly advised and reminded against using cannabis throughout the study duration.

The table in Appendix 1 provides a listing of prohibited medications; however, it is not a comprehensive list of all restricted medications, supplements, and other therapeutics.

Consult the Sponsor's Medical Monitor or designee if there is any uncertainty regarding a subject's use of a particular drug, drug class, supplement, or other therapeutic.

Once an ICF is signed, subjects on prohibited medications, supplements, or other therapeutics may taper off that substance and participate in the study if the required washout conditions have been met. For prescribed medications to treat ADHD, anxiety, depression or other mood disorders, the medication taper will be overseen by the treating physician or a study physician based on acceptable local practice standards.

7.7. Intra-subject Variability due to LSD Metabolism

Use of potent CYP2D6 inhibitors is excluded; moderate CYP2D6 inhibitors are allowed by Investigator or medical supervisor discretion. See Appendix 3 for a list of potent and moderate CYP2D6 inhibitors.

In vitro studies suggest that certain cytochrome P450 (CYP) enzymes contribute to the metabolism of LSD (Luethi 2019). In particular, genetically determined CYP2D6 functionality significantly influenced the pharmacokinetics of oral LSD in healthy volunteers (Vizeli 2021). Participants with no functional CYP2D6 (i.e., poor metabolizers) had longer LSD half-lives and approximately 75% higher parent drug and O-H-LSD plasma exposure compared with carriers of functional CYP2D6.

The CYP2D6 phenotype of study subjects will not be known prior to dosing and thus, subjects must be reminded not to take any medication, herbal, or over the counter medication without consulting the site Investigator.

7.8. Withdrawal from Study

Subjects may be withdrawn at any time. Once dosing has occurred, every attempt should be made to continue assessments to ensure the safety of the subject. Specific reasons for withdrawing a subject should be documented in the eCRF, and include, but are not limited to:

- Subject’s decision
- The Investigator or Sponsor judges that it is in the best interest of the subject to stop study treatment due to any abnormality compromising subject safety
- Pregnancy
- Severe noncompliance to the study protocol as judged by the Investigator and/or the Sponsor.

Subjects who prematurely discontinue may be replaced, per Sponsor decision. Subjects will not be allowed to re-enter the study.

7.8.1. Procedures for withdrawal of a subject from the study

Subjects are free to withdraw their consent to participate in the study at any time, without prejudice. Such subjects will always be asked about the reason(s), and the presence of any adverse events. If possible, subjects who withdraw from the study after dosing and before completion should be seen by an Investigator, and undergo the assessments and procedures scheduled for the follow-up visit/end of study visit. Adverse events should be followed up.

7.9. Audits and Inspections

Authorized representatives of the Sponsor, Health Authority, or an Ethics Committee may perform audits or inspections at the site, including source data verification. The purpose of an audit or inspection is to examine all study-related activities and systematically and independently document, to determine whether these activities were conducted, and data were recorded, analyzed, and accurately reported according to the protocol, GCP, guidelines of the ICH, and any applicable regulatory requirements. The Investigator will contact the Sponsor immediately if contacted by a regulatory agency about an inspection at the center.

8. COLLECTION OF STUDY VARIABLES

8.1. Schedule of Events

The study assessments are described in the sections below and the timing of these assessments are detailed in the study plan.

8.2. Table 1: Schedule for study specific assessments

	Screening	Baseline[a]	Dosing begins	6-week Blinded Treatment Period (20 µg LSD vs. Placebo)			End of Study/Early Termination
Time	Up to 4 weeks prior to Day -1	Day -1	Day 1	Twice weekly Dosing* (start = Day 1)	Every 2 weeks (first dose of the week)**	Week 6 (before final dose)***	Week 10 follow-up or final visit if withdrawing****
Medical/surgical history	X						
Inclusion/exclusion criteria	X	X					
Pregnancy test (for women without documentation of sterilization)	X		X	X			X
Physical examination	X					X	X
Weight and BMI	X					X	X
Height	X					X	X
Vital signs	X	X	X	X		X	X
Clinical Laboratory Tests	X				X	X	X
Pharmacokinetics			X[1]				
Urine Drug test	X	X	X		X[2]	X[2]	
Pharmacodynamics (5DASC, & MEQ30)			X			X[3]	
12-lead ECG[b]	X		X[4]			X[4]	
Cognitive Performance Test		X				X	
CAARS[c]	X	X			X	X	X
ASRS		X			X	X	X
AISRS, CGI-S	X	X			X	X	X
MINI	X						
C-SSRS	X						

C-SSRS since last visit (SLV)		X	X	X	X	X
VAS			X[5]			
Check daily diary	X	X	X	X	X	X
Randomization			X			
Drug administration			X	X		
Adverse events	X	X	X	X	X	X
Concomitant medication	X	X	X	X	X	X

5D-ASC = 5 dimensions of altered states of consciousness scale; AISRS = Adult ADHD Investigator Symptom Rating Scale; ASRS = Adult ADHD Self-Report Scale; BMI = body mass index; BP = blood pressure; CAARS = Conners' Adult ADHD Rating Scale; CGI-S = Clinical Global Impression Scale; C-SSRS = Columbia-Suicide Severity Rating Scale; ECG = electrocardiogram; MEQ30 = Mystical Experience Questionnaire (30-item); VAS = Visual Analog Scale.

* Twice weekly dosing occurred every 3 to 4 days (e.g., every Monday and Wednesday) with a ± 1 day window.

** Outcomes of weeks 2 and 4 were assessed at the beginning of the respective dosing week. Week 2 was at day 8 or after 2 doses; week 4 at day 22 or after 6 doses. AISRS and CGI week 6 was assessed before the last dose at day 40.

*** CAARS and ASRS were assessed twice during week 6 (at day 36 and 40).

*** For persons withdrawing from the study the final visit was coordinated to occur up to 4 weeks after the last administered dose.

a Baseline visit could be done up to 3 days prior to Day 1 e.g., if it was done on a Friday and Day 1 on a Monday.

b Resting in supine position for 5 minutes prior to ECG collection.

c CAARS-O-RS conducted at screening. All other assessments and shown results used CAARS-L-SR.

X[1] PK samples collected pre-dose, 0.5, 1, 2, 3, 4, and 6 hours post-dose.

X[2] 2 random drug tests were administered during the study.

X[3] Pharmacodynamic scales completed at home up to 6 hours after dosing.

X[4] 2 hours after dosing.

X[5] Collected pre-dose, 0.5, 1, 2, 3, 4, and 6 hours post-dose

8.3. Visits and Assessments

All visits must be monitored for safety and AEs recorded.

8.3.1. Screening visit

Screening visits can be scheduled over the course of 4 weeks prior to the baseline visit.

Study activities performed:

Informed consent, urine drug screen, concomitant medication assessment, demography (age, sex, profession, level of education), medical/surgical history, inclusion/exclusion criteria check, MINI, pregnancy test, physical examination (including weight, height, calculated BMI, vital signs [body temperature, heart rate, supine BP, RR]), clinical laboratory tests, 12-lead ECG, AISRS and CGI-S, CAARS-O-RS, C-SSRS (Screening), and record adverse events. If eligible for participation, the subject is also given a diary at least 7 days prior to the scheduled baseline visit and instructed on its use. Subjects will be shown the cognitive performance tests and receive training in how to complete them at the Screening visit.

8.3.2. Baseline (within 3 days of Day 1, [± 1 day])

The baseline visit (also called Day -1) should be the nearest day to the first day of dosing (i.e., Day 1). Because clinics may be closed on the weekends, a Friday visit is allowed for subjects whose Day 1 is on Monday.

Study activities performed:

Urine drug screen, vital signs, cognitive performance tests, AISRS and CGI- S, ASRS and CAARS-L-SR, C-SSRS -SLV, record adverse events and concomitant medications, check daily diary.

After completion of the study activities above, the inclusion/exclusion criteria are verified.

8.3.3. Day 1 dosing (± 1 day)

Randomization to MM-120 or placebo will occur before the first dose on Day 1. The first dose must be administered in the clinic. For this visit, the subject will remain at the testing facility for 6 hours after drug administration for blood sampling and getting familiar with the effects of LSD.

Study activities performed before dosing:

Urine drug screen, vital signs, pregnancy test, C-SSRS (Since Last Visit), record adverse events and concomitant medications, check daily diary.

Study activities performed after dosing:

Blood samples for PK are taken repeatedly at 0, 0.5, 1, 2, 3, 4, 6 hours after dosing.
Pharmacodynamics are measured over 6 hours including subjective effects as assessed using VAS scales, blood pressure and heart rate. 12-lead ECG will be performed at 2 hours post dosing. The 5D-ASC and MEQ30 are assessed 6 hours after dosing.

8.3.4. Post Day 1 dosing visits

Following Day 1 dosing, all visits may be conducted remotely per Investigator decision as long as all activities can be conducted per protocol, e.g., calibrated blood pressure cuff, cognitive scale.

Any visit conducted remotely due to COVID-19 requires Sponsor Medical Monitor and operations team notification and conduct under urgent safety measures and all protocol deviations captured in EDC designated COVID-19 deviation.

The reason for a remote visit must be captured in the EDC. The site will choose one of the following reasons:

1. COVID-19
2. Convenience for study subject
3. Site space considerations to conduct in clinic visits
4. Other

8.3.4.1. Week 2 visit

At the first visit after 1 week of treatment (Day 8 or after), AISRS and CGI-S will be conducted in addition to other required study activities. These activities will be performed pre-dose on the 3rd dosing visit.

8.3.4.2. Twice weekly dosing

Dosing (placebo/MM-120) must be completed by a trained study personnel with delegated authority for drug dispensing/ dosing. At each visit, adverse events and concomitant medications will be recorded, vital signs, C-SSRS (Since Last Visit) administered, and daily diary checked. Pregnancy testing will occur predose at each dosing visit or a minimum of once weekly.

8.3.4.3. Every 2 weeks prior to first dose of the week

At each visit that occurs every 2 weeks, clinical laboratory tests, ASRS and CAARS-L-SR, and pregnancy test will be conducted. Two (2) random urine drug screens will also be conducted during the study. When combined with a dosing visit, regular activities scheduled to be conducted at each dosing visit will also be completed (see Section 6.2.4.2).

8.3.4.4. Week 6 (last dose)

Study activities performed before dosing:

Urine drug screen, C-SSRS (Since Last Visit), record adverse events and concomitant medications, check daily diary, physical examination (including weight, height, calculated BMI, vital signs [body temperature, heart rate, supine BP, RR]), cognitive performance tests, clinical laboratory tests, ASRS and CAARS-L-SR, AISRS, and CGI-S.

Study activities performed after dosing:

12-lead ECG conducted at 2 hours post dosing. Pharmacodynamics using 5D-ASC and MEQ30 at 6 hours after dosing (subject completes at home).

8.3.5. Week 10 follow-up / End of Study or Early Termination (if subject withdraws before last follow-up visit [± 3 days])

The Week 10 follow-up visit may be conducted remotely per Investigator decision. The reason for conducting the visit remotely must be recorded in the EDC (as listed in Section 6.2.4), and if the reason is due to COVID-19, the Sponsor Medical Monitor and operations team must be notified and the visit conducted under urgent safety measures and all protocol deviations captured in the EDC.
Pregnancy test, end of study physical examination (including weight, height, calculated BMI, vital signs [body temperature, heart rate, supine BP]), clinical laboratory tests, ASRS, CAARSL-SR, AISRS, CGI-S, C-SSRS (Since Last Visit) will be conducted at the last visit, adverse events and concomitant medications will be recorded, and daily diary checked.

8.3.6. ADHD assessments

8.3.6.1. Adult ADHD Investigator Symptom Rating Scale

The Adult ADHD Investigator Symptom Rating Scale (AISRS) is a clinician-administered scale that assesses each of the DSM-5 symptoms of ADHD. The AISRS not only uses the adult ADHD prompts from the Adult ADHD Clinical Diagnostic Scale (ACDS) v1.2, but each of the stem questions are designed to better capture symptoms of the disorder as they present in adulthood. It has been used and validated many times in a variety of clinical drug trials.
The AISRS total score consists of 18 items from the original ADHD-RS, which were derived based on DSM- 5 criteria for ADHD. The ADHD-RS includes 9 items that address symptoms of inattention, and 9 items that address symptoms of impulsivity and hyperactivity. Each item is rated from 0 to 3. The AISRS total score can range from 0 to 54. A higher score corresponds to a worse severity of ADHD.

8.3.6.2. Clinical Global Impression – Severity scale (Investigator Assessed)

The Clinical Global Impression (CGI) rating scales are measures of symptom severity, treatment response, and the efficacy of treatments in treatment studies of subjects with ADHD. The scale has been validated.
The Clinical Global Impression – Severity scale (CGI-S) is a 7-point scale that requires the clinician to rate the severity of the subject's illness at the time of assessment. Possible ratings are:

- normal, not at all ill
- borderline mentally ill
- mildly ill

- moderately ill
- markedly ill
- severely ill
- among the most extremely ill subjects

The CGI-S is administered by a trained investigator.

8.3.6.3. Adult Attention-Deficit/Hyperactivity Disorder Self-Reporting Rating Scale

The Adult Attention-Deficit/Hyperactivity Disorder Self-Reporting Rating Scale (ASRS) was developed in conjunction with the World Health Organization (WHO), and the Workgroup on Adult ADHD. The ASRS has 18 questions, which are consistent with the DSM-5 criteria, and address ADHD symptoms in adults. The ASRS was externally validated on approximately 60 adult subjects and showed high internal consistency and high concurrent validity with the physician administered ADHD rating system. Self-rating scales are generally useful tools because they create a way to collect a large, accurate amount of data in an organized, quick, and cost-effective way.
It uses a scale that ranges from 0-4 based on the individuals mark in either "never, rarely, sometimes, often, very often" column for a possible total score of 72. Each column is used to describe the severity of the individual's symptoms based on the question asked. The participant is asked to make a mark within one column for each question that best describes their answer. The questions are split up into 2 separate parts; Part A consists of questions 1-6, and Part B consists of questions 7-18. If four or more answers are scored “positive” then a high consistency of ADHD is indicated and further follow-up with a licensed clinician is necessary. Positive scores in Part B can be further identifying cues of the individual's symptoms.
There have been studies done to test the validity and reliability of the ASRS by comparing results of participants that were diagnosed with childhood ADHD, after completing the selfreporting on their own, and then were subsequently tested with a clinician. There was a high correlation between the grouped symptoms (hyperactivity/impulsiveness and inattentiveness) when comparing the administration results of the tests. After an experiment with 60 adults whom completed the self-rating scale, and whose results were compared to their ratings with an administered ADHD rating scale, the results showed that the self-rating scale has a high internal reliability and also validity.

8.3.6.4. Connors' Adult ADHD Rating Scale

The Connors' Adult ADHD Rating Scale (CAARS) Self-Report Long Form (CAARS-L-SR) is a 66-item measure of ADHD symptoms that was designed as a self-report assessment for adult ADHD. Responses are scored on a 4-point scale, where 0 = not at all, 1 = just a little, 2 = pretty much, and 3 = very much. Test – retest reliabilities have been shown to be strong. The CAARS is highly correlated with other self-report ADHD measures, and initial studies on diagnostic accuracy for adult ADHD found the CAARS to have a good diagnostic sensitivity and specificity relative to healthy controls. The Connors' Adult ADHD Rating Scale Observer Short Screening (CAARS-S-OR) version will be used during screening. The 30-item version includes DSM-IV criteria for ADHD and takes approximately 10 min to complete. The scale yields scores for 1) inattentiveness, and 2) hyperactivity/impulsivity symptoms according to DSM-IV, and 3) an ADHD index which allows best to differentiate between persons with ADHD and normal persons. Item scores are summed to three main scores which are then transformed using population-derived age- and sex-adjusted norm values to a T-score. A Tscore < 60 indicates no ADHD. A T-score of 60-64 indicates borderline ADHD. A T-score of > 64 indicates ADHD.

8.3.7. Daily Diary

To explore sleep quality and duration, the subjects will be given a home diary for recording sleep parameters. Details of the home diary will be described in a separate document. Analysis will be specified in the SAP.

8.3.8. Cognitive Performance Tests (Computer Test)

The cognitive performance tests are objective measurements of attention to a task(s). Tests are performed at Baseline and Week 6 (prior to last dose).

8.3.8.1. Stop-signal task

The stop-signal task requires subjects to make quick key responses to visually presented go signals, and to inhibit any response when a visual stop signal is presented (Fillmore et al., 2002; Kuypers et al., 2007). The go signals are four 1.5 cm letters (A, B, C, D) presented one at a time in the center of a computer screen.

A single test consists of 176 trials in which each of the four-letter stimuli will be presented equally. A stop signal occurs in 48 trials during a test. The stop signal consists of a visual cue, i.e., “*”, that appears in one of the four corners of the screen. The subjects are required to withhold any response in case a stop signal is presented. The task has a duration of 10 minutes. The dependent variables are the proportion of correct Go and Stop responses, and the stop reaction time. The stop reaction time (SSRT) to stop signal trials represent the estimated mean time required to inhibit a response. The SSRT is used as a clinical index of inhibitory control, primarily in the study of ADHD, where impulsive subjects have slower SSRTs (Logan et al., 1997; Oosterlaan et al., 1998; Rubia et al., 1998). Higher numbers indicate more difficulty inhibiting responses. Task duration is 10 minutes.

8.3.8.2. Discounting task

In the delay discounting task subjects are presented 114 questions. For each question, subjects have to indicate which of two hypothetical rewards they prefer, the ‘standard’ or the ‘alternative’ item. The standard item, 100 Swiss francs, is available after one of five delays (0, 7, 30, 180, or 365 days). The alternative item is an amount of money (1, 25, 50, 100, 150, 200, 250, 300, 350, 400, 450, 500, 550, 600, 650, 700, 750, 800, 850, 900, 950, 1000, or 1050 Euros (Maastricht site)/Swiss francs (Basel site) available after 0 days). Each combination of standard and alternative item is only used once and presented in random order. Subjects are asked to express their preference for an item by pressing one of two buttons. Recorded response choices are used afterwards to calculate the switch points per delay at which the subject preferred the alternative item over the standard item. Task duration is 10 minutes. Previous research demonstrates excessive delays discounting among individuals with ADHD (Kuypers et al., 2007; Mitchell, 1999).

8.3.8.3. Psychomotor vigilance task

The psychomotor vigilance task (PVT) is a sustained attention task, measuring reaction time in response to a visual stimulus over a period of 10 minutes. Subjects have to react to the onset of a counter as quickly as possible by pressing a response button. Outcome measures are mean reaction time (milliseconds) and number of attention lapses, defined as a failure to react, or any reaction exceeding 500 msec (Dinges et al., 1985).

8.3.8.4. Time Production task

During the time production (TP) task (Hurks et al., 2014), an aspect of time perception is measured, namely the ability to estimate and produce the duration of a time interval that is presented in numbers. Subjects are presented with a stimulus depicting a candle and a number indicative of a time interval (3, 6, 12, 15, 30, or 45 seconds). When this stimulus appears, subjects have to press the space bar and are asked to hold the button as long as the number next to the candle indicates (e.g., 12 s). As soon as the space bar is released, the stimulus disappears from the screen and the procedure repeats itself. The six-time intervals (3, 6, 12, 15, 30, or 45 seconds) will be presented twice in random order, summing up to 12 trials in total. It will take about 5 minutes to complete the task. Dependent variables include the mean duration judgement ratio score; first the duration judgement ratio is calculated by taking the ratio of subjective and objective duration in milliseconds (i.e., the duration judgment ratio) for each trial; these are averaged for each stimulus duration (i.e., 3, 6, 12, 15, 30, or 45 seconds). A score smaller than 1 represents an underestimation of the stimulus duration, whereas a score larger than 1 represents an overestimation of the stimulus duration. A mean score will be calculated by averaging all six duration judgment scores.

8.3.8.5. Time Reproduction task

During the time reproduction (TR) task (Hurks et al., 2011), the ability to reproduce a time interval is measured. During this task, subjects will be shown a stimulus (i.e., a row of light bulbs, of which only one is turned on) that is presented for a certain time interval (3, 6, 12, 15, 30, or 45 seconds). This stimulus is followed by a fixation point for 1000 milliseconds. After this, the same stimulus (i.e., a row of light bulbs, of which only one is turned on) is shown again. Once the row of light bulbs appears, subjects have to press the space bar and hold this for as long as the row of light bulbs appeared to them before, thereby reproducing the time interval. As soon as the space bar is released, the light bulb switches off and the procedure repeats itself. The Good Distribution Practice six-time intervals (3, 6, 12, 15, 30, or 45 seconds) will be presented twice in random order, summing up to 12 trials in total. It will take 10 minutes to complete the task. Dependent variables include the mean duration judgement ratio score; first the duration judgement ratio is calculated by taking the ratio of subjective and objective duration in milliseconds (i.e., the duration judgment ratio) for each trial; these are averaged for each stimulus duration (i.e., 3, 6, 12, 15, 30, or 45 seconds). A score smaller than 1 represents an

underestimation of the stimulus duration, whereas a score larger than 1 represents an overestimation of the stimulus duration. A mean score will be calculated by averaging all six duration judgment scores.

9. SAFETY

9.1. Physical Risks to the Subjects

MM-120 use is not associated with any known physical risks, but it may produce psychiatric complications as described above and below.

9.1.1. Expected acute adverse effects

The low dose (20 µg) of MM-120 used in the present study is expected to produce minimal subjective and physiological acute adverse effects, including subtle acute feelings of drug effect, and slight impairment of cognition (Bershad et al., 2019; Yanakieva et al., 2019). Very susceptible persons could potentially experience effects more typically experienced with high doses of LSD, including dysphoria, anxiety, mood swings, dream-like state, transient depersonalization and derealization phenomena, mild and transient paranoid thinking, negative experiences (anxiety, dysphoria, bad trips), tremor, restlessness, acute perceptual changes, acutely impaired psychomotor function, mild tachycardia, mild hypertension, nausea, headache, dizziness, trembling, lack of appetite (Dolder et al., 2016; Gasser, 2012b; Johnson et al., 2012; Passie et al., 2008; Schmid et al., 2015; Studerus et al., 2011). Adverse effects at a dose of 100 µg are expected to completely subside within 24 h at most (Dolder et al., 2016). No severe or serious adverse effects are expected based on previous studies using a 20 µg dose of MM-120.

9.1.2. Possible lasting adverse effects

At high doses of LSD, and in rare cases, flashback phenomena psychotic reactions have been reported. Negative experiences (bad trips) and flashback phenomena may occur, generally in uncontrolled conditions and when high doses of LSD are used (Strassman, 1984).

9.1.3. Venipuncture

There is a risk for pain, bruising and thrombophlebitis due to venipuncture.

9.2. LSD-Specific Toxicity Considerations

The primary safety concerns with hallucinogen research are psychological rather than physiological in nature (Johnson et al., 2008). Even under unsupervised and unprepared conditions, reactions to hallucinogens involving violence or self-destructive behavior are rare, and it is important not to create an unrealistic account of the dangers of hallucinogens (Johnson et al., 2008). Nonetheless, even infrequent reports of such dangers require that we take seriously such risks and take steps to avoid their occurrence. Subjects must receive the first dose of MM120 in the clinic under supervision to assess for unusual or exaggerated responses and should be taken under direct supervision of study personnel to avoid substance deviation. For home medication, study medication under the Investigator’s supervision may be administered at home to the subject to assure study medication compliance and safety and perform all protocol procedures including AE/SAE, concomitant medication, and C-SSRS assessment. Subject cannot drive or operate heavy machinery or do anything else that would be dangerous in an impaired state for the remainder of the day after receiving IMP.

9.2.1. Psychological effects

There is currently no evidence for positive or adverse psychological effects from controlled studies on repeated low dosing LSD. Survey studies among persons performing repeated low dosing explored concerns relating to adverse effects. Reported concerns were stronger-thanexpected psychedelic effects, anxiety, and physical adverse side effects (Lea et al., 2020). Other perceived limitations include issues related to dosing, taking illegal substances, limited or no mental health or cognitive improvement, unpleasant “off” days, only short-term benefits, and concerns about dependence and drug-related risks (Lea et al., 2019). In the present study, the first dosing will be under full supervision at the research sites. The use of MM-120 as study medication will be legal, and the administration of the substance will always be by the study personnel or observed by study personnel through a video conference, and as pure substance in a precisely known dose. At the dose used, MM-120 should not produce relevant autonomic system stimulation based on previous studies. However, it is possible that

MM-120 will not produce an improvement of ADHD symptomatology, and may not be effective overall, and may even worsen symptoms in some subjects. This will be tested in the present study. When used at high doses, LSD and other psychoactive substances induce mild or moderate anticipatory anxiety at the beginning of the onset of the drug effect (Griffiths et al., 2006; Liechti et al., 2001; Schmid et al., 2015). Any such reactions are expected to resolve spontaneously with supportive care by the investigators (Griffiths et al., 2011; Griffiths et al., 2006; Schmid et al., 2015). At the dose of MM-120 to be used in the present study, subjects are expected to retain their thought control, and in contrast to psychotic subjects, subjects will remain aware of the transient state of the drug-induced experience if they should at all realize any change. Events of more pronounced anxiety, panic attacks or agitation are highly unlikely to occur. In the case of any psychiatric complications after the study session, and also if the subjects would like to discuss negative experiences in association with the study, they can contact the study physician, who will offer further assistance beyond the testing days.

9.2.2. Self-injurious behavior

People who have taken LSD in uncontrolled settings may engage in reckless behavior, such as driving while intoxicated. This risk is greatly reduced by the use of very low doses of LSD. In the current study, subjects will take MM-120 under supervision, and remain at the study site for the first dosing to assess for any unusual or exaggerated effects, and when the dose is given at home it will be under the supervision of the investigator to assure compliance and safety. Prolonged psychiatric symptoms and/or psychosis after low dose LSD are rare reactions that are unlikely to occur at the dose used in this study, and particularly in the cohort of nonpsychotic subjects to be included in this study. LSD may trigger psychotic episodes in people already vulnerable to psychosis rather than directly causing it.

9.2.3. Columbia-Suicide Severity Rating Scale

The Columbia-Suicide Severity Rating Scale (C-SSRS) is a validated outcome assessment that assesses a subject’s suicidal ideation or behavior over a specified time interval (Posner et al., 2011). Suicidal ideation is assessed on a scale from 1 (wish to be dead) to 5 (active suicidal ideation with specific plan and intent). Suicidal behavior is assessed based on responses to individual prompts about prior suicidal actions. The C-SSRS will be assessed at all study visits.

9.2.4. Reproductive and developmental risks

There are mixed results from preclinical studies evaluating reproductive toxicity, chromosomal aberration, and mutagenic studies (refer to the MM-120 Investigator’s Brochure for details). Thus, to participate in this study, women of childbearing potential (WOCBP) (i.e., physiologically capable of becoming pregnant) must agree to use a highly effective method of contraception for the duration of the study, and non-sterilized men who are sexually active with a WOCBP must agree to use barrier contraception (e.g., condom with or without spermicidal cream or jelly). Subjects who do not agree to these conditions are not eligible to participate in the study.

Appendix 2 provides definitions of WOCBP and highly effective methods of contraception as well as information about unacceptable methods of contraception. Subjects will be provided with information on acceptable methods of contraception as part of the informed consent process and will confirm when they sign a consent form that they understand the requirements for avoidance of pregnancy in the subject or the subject’s sexual partner during the course of the study.

Additional Information for Female Subjects

WOCBP may participate in the study only if they have a negative urinary pregnancy test (beta human chorionic gonadotropin [β-HCG]) result at specified visits prior to dosing (i.e., Screening [Visit 1] and Day 1) and every 2 weeks during the dosing phase of the study (through Week 6).

Female subjects who are currently or planning to become pregnant or breastfeed during the study period are excluded. If a female subject withdraws early from the study, she should be advised not to become pregnant or breastfeed for 3 months after last dose of study drug. Any subject that becomes pregnant after Day 1/Dose through Week 6 is required to withdraw from the study. The subject will be monitored for the outcome of the pregnancy, and reporting will occur as described in Section 7.8.

Additional Information for Male Subjects

Male subjects should be encouraged to advise any WOCBP who are their sexual partners to use a highly effective method of contraception in addition to the male subject’s use of barrier contraception. A female condom and male condom should not be used together.

Male subjects who are planning to donate sperm during the study period are excluded. If a male subject withdraws early from the study, he should be advised not to donate sperm for at least 28 days after he received the study drug.

9.2.5. Abuse liability

MM-120 is not associated with dependence. Hallucinogens are not self-administered by animals, and there is no human LSD dependence syndrome (Passie et al., 2008). Subjects with current substance use are not included in the study, but substance use disorder in the past is not an exclusion criterion. Hallucinogens have been used and are being investigated in several substance use disorders, including opioid (Belleville et al., 1956; Ross, 2012; Savage et al., 1973), alcohol (Bogenschutz et al., 2015; Krebs et al., 2012; Kurland et al., 1967; Liester, 2014; Ludwig et al., 1969; Mangini, 1998; Pahnke et al., 1970), and nicotine dependence (Johnson et al., 2014). Illicit drug use will be monitored during the study using repeated urine drug screens.

9.2.6. Neurotoxicity

There is no evidence that the active drug substance of MM-120 (LSD) is neurotoxic (Nichols, 2016; Passie et al., 2014; Passie et al., 2008).

9.2.7. Flashbacks

Flashbacks can be defined as episodic and short (seconds or minutes) replications of elements of previous substance-related experiences (Holland et al., 2011; Passie et al., 2014). These experiences can be positive or negative. Such phenomena have been reported after the use of many substances and are also prevalent in non-substance using persons (Holland et al., 2011). Clinically significant flashbacks are also defined as hallucinogen persisting perception disorder. This disorder is considered rare, but may occur in subjects with anxiety disorders, and it typically will have a limited course of months to a year (Halpern et al., 1999; Holland et al., 2011; Passie et al., 2014). At a follow-up one month after LSD (200 µg) administration, none of 16 healthy subjects reported any perceptual changes (e.g., flashbacks) (Schmid et al., 2018). With regards to the present study, no visual alterations are expected at the doses used, and therefore flashbacks are very unlikely to occur.

9.3. Safety Monitoring

Guidelines for hallucinogen research have been published that relate to high dose (approximately 100 µg and above) use of psychedelics, and not to the low dose (20 µg) used in the present study (Fischman et al., 1998; Gouzoulis-Mayfrank et al., 1998; Johnson et al., 2008). The procedures are intended to support the safe administration of psychoactive substances while minimizing potential adverse reactions. The dose of MM-120 used in the present study is much lower at 20 µg, and safety data is based on previous clinical studies in healthy volunteers.

9.3.1. Other psychiatric disorders

The psychiatric screening criteria are important for minimizing the already low chances of precipitating a longer-term psychotic reaction to MM-120. Subjects who have a present or past history of meeting DSM-5 criteria for schizophrenia, or other psychotic disorders (or due to a medical condition), or bipolar disorder are excluded. The above are the most important conditions to exclude for ensuring safety. Subjects with a first-degree relative with these disorders are also excluded. Other psychiatric disorders such as co-morbid depression, obsessive-compulsive disorders, anxiety or previous substance use disorder are not excluded because hallucinogens have been used in subjects with these disorders, or specifically to treat these disorders (Gasser, 2012a; Gasser et al., 2015; Grob et al., 2011; Krebs et al., 2012; Moreno et al., 2006; Ross, 2012).

9.3.2. Predictors for response

Relevant for high-dose psychedelic research, factors that predict more pleasant and mysticaltype experiences following hallucinogen administration include high scores of the personality trait of absorption (open to new experiences) and having experienced few psychological problems in the past weeks before the test sessions (Studerus et al., 2012). Factors associated with an unpleasant and/or anxious reaction to the hallucinogen are low age, emotional lability, and a setting involving a brain scan (Johnson et al., 2008; Studerus et al., 2012). With regard to personality, subjects who are more open to new experiences, including the use of hallucinogens, will more likely be interested in participating in the study, and this self-selection bias enhances the safety of such research (Johnson et al., 2008; Studerus et al., 2011).

9.3.3. Drug experience

Previous experience with psychoactive drugs may influence the response to psychoactive substances. In controlled studies with psilocybin, drug use and pre-experience with hallucinogens only moderately affected the psilocybin response (Studerus et al., 2012). Hallucinogen-naïve subjects tended to report overall stronger psilocybin effects (Studerus et al., 2012). Subjects who sometimes smoked cannabis (more than once per month) experienced more pleasurable effects and a trend towards less anxiety compared to subjects who rarely used THC (Studerus et al., 2012). No difference was found in the response to LSD between healthy subjects with previous experience with hallucinogen use and hallucinogen-naïve subjects (Schmid et al., 2015). Of note, subjects with regular drug use were not included in the above study (Studerus et al., 2012). The present study will also include mostly subjects with no or only limited previous drug exposures, similar to previous studies using LSD (Dolder et al., 2017b; Gasser et al., 2014; Gasser et al., 2015; Schmid et al., 2015), and similar to studies conducted by others using psilocybin (Griffiths et al., 2011; Griffiths et al., 2006; Studerus et al., 2011).

9.4. Safety Procedures During the Session

9.4.1. Adverse cardiovascular effects

No clinically relevant cardio-stimulant effects are expected at the dose of MM-120 to be used in the present study. Only mild cardio-stimulant effects are expected at high doses of LSD (Dolder et al., 2016). Cardiovascular effects (blood pressure and heart rate) will be repeatedly measured during the first dosing to document the absence of relevant effects. Closer monitoring would be implemented in the unexpected case of blood pressure values exceeding 180/120 mm Hg or decrease below 90 mm Hg for systolic blood pressure. Treatment of a hypertensive reaction (P_{sys} > 220 mmHg) may include lorazepam and nitroglycerine, per Investigator decision and Sponsor medical monitor notified. Treatment of hypotension would include Trendelenburg position. Cardiac arrest would trigger immediate cardiopulmonary reanimation and a call to the ambulance.

9.4.2. Headaches

MM-120 may produce transient headaches (Schmid et al., 2015). One participant (out of 16) required acetaminophen for a moderate headache the day after a LSD session. Conversely, the active drug substance LSD reportedly reduces episodes of cluster headache and migraine (Davenport, 2016; Karst et al., 2010; Sewell et al., 2006), and is currently being tested for these indications in the context of compassionate use, and with authorization of the Swiss Federal Office of Public Health (BAG) in selected cases. In the pilot study with subjects suffering from anxiety associated with life-threatening diseases, LSD markedly reduced the number of migraine attacks in two migraine subjects (Gasser et al., 2014).

9.4.3. AEs between sessions

AE and SAE will be assessed at each study visit and at the follow-up visit at the end of the study.

9.5. Risk Benefit Assessment

9.5.1. Potential benefits

Based on previous anecdotal reports from LSD users practicing repeated low dosing, MM-120 may provide a benefit to ADHD subjects. There is no preliminary data from controlled studies to support a benefit. Therefore, this investigation is the first to explore potential beneficial effects of low doses of LSD in a proof-of-concept study. The study will provide information on the efficacy of MM-120 in the treatment of ADHD, and therefore potentially provide benefit to future subjects, or if efficacy is not supported, help to avoid unnecessary treatment.

9.5.2. Potential harms to subjects

The risk of lasting psychological harm is considered low, and the risks of physical harm very low (see above). The risk of not being provided alternative treatment is minimal and temporary because only subjects who have stopped other treatment options are included or subjects who do not mandatorily need other ADHD medication. The subjects might experience some disadvantage when they stop their medication, which is evaluated as low risk and not life threatening. Subjects are free to stop their study participation at any time without any justification. There are no financial risks. Travel expenses are reimbursed to the subjects.

9.6. Safety Definitions

9.6.1. Adverse Event

An Adverse Event (AE) is any untoward medical occurrence in a clinical trial subject administered an IMP and which does not necessarily have a causal relationship with this treatment. An AE can therefore be any unfavorable and unintended sign (including an abnormal laboratory finding), or any clinically significant change from baseline, whether considered positive (e.g., marked feelings of a drug high, feeling happy) or negative symptom (e.g., feeling panicked, fearful, or sad), change in sleep, quality or duration, or disease temporally associated with the use of an IMP, whether or not considered related to the IMP.

9.6.2. Adverse Reaction

An Adverse Reaction (AR) is any untoward and unintended responses to an IMP judged by Investigator/Sponsor as having a reasonable causal relationship to the IMP. The expression reasonable causal relationship means to convey in general that there is evidence or argument to suggest a causal relationship.

9.6.3. Unexpected Adverse Reaction

An Unexpected Adverse Reaction (UAR) is an adverse reaction (AR), the nature or severity of which is not consistent with the applicable product information (e.g., Investigator's brochure [IB] for an unapproved investigational product or summary of product characteristics [SmPC] for an authorized product). When the outcome of the adverse reaction is not consistent with the applicable product information, this adverse reaction should be considered as unexpected. Side effects documented in the IB, or SmPC which occur in a more severe form than anticipated, are also considered as being unexpected.

9.6.4. Serious Adverse Event or Serious Adverse Reaction

A Serious Adverse Event (SAE) is any untoward medical occurrence or effect that at any dose results in death, is life-threatening, requires hospitalization, or prolongation of existing hospitalization, results in persistent or significant disability, or incapacity, or is a congenital anomaly or birth defect. In this context, the term life-threatening refers to an event in which the trial participant was at immediate risk of death at the time of the event; it does not refer to an event which might have caused death if it were more severe.

A Serious Adverse Reaction (SAR) is an SAE that is determined to be related to the intervention. Based on the causality definitions in Section 10.7, SAEs that are determined to have a causal relationship of "possible", "probable" or "definitely" will be considered SARs.

9.6.5. Suspected Unexpected Serious Adverse Reaction

A Suspected Unexpected Serious Adverse Reaction (SUSAR) is any suspected adverse reaction related to an IMP that is both unexpected and serious.

The Investigator evaluates SAEs for seriousness and causality in consultation with the Sponsor Medical Monitor, as necessary. Expectedness is also determined by the Sponsor's Medical Monitor based on an assessment of the adverse event in the context of previously reported adverse events, as outlined in the Investigators' Brochure and/or study protocol. If the event is related to the IMP and is both serious and unexpected, it is classified as a SUSAR.

For purposes of defining a SUSAR, any SAE that has a causality of "possibly", "probably" or "definitely" will be considered to be related to the IMP.

9.7. Causality

Most adverse events and adverse reactions that occur in this study, whether they are serious or not, will be expected treatment-related toxicities due to the medication used in this study. The assignment of the causality will be made by the Investigator using the definitions in the table below.

Relationship	Description
Unrelated	There is no evidence of any causal relationship.

Unlikely	There is little evidence to suggest there is a causal relationship (e.g., the event did not occur within a reasonable time after administration of the IMP). There is another reasonable explanation for the event (e.g., the participant's clinical condition, other concomitant treatment).
Possible	There is some evidence to suggest a causal relationship (e.g., because the event occurs within a reasonable time after administration of the IMP). However, the influence of other factors may have contributed to the event (e.g. the participant's clinical condition, other concomitant treatments).
Probable	There is evidence to suggest a causal relationship and the influence of other factors is unlikely.
Definitely	There is clear evidence to suggest a causal relationship and other possible contributing factors can be ruled out.

9.8. Documentation

All adverse events occurring after the subject has signed the informed consent will be fully recorded in the subject's eCRF. Each event will be described in detail, along with start and stop dates, severity, relationship to IMP, action taken and outcome.

9.8.1. Adverse events documentation

AEs will be described and recorded on the subject's eCRF, regardless of the severity or relationship to the IMP. AEs are rated for severity, and the potential relationship regarding the study interventions will be evaluated by the Investigator according to standard criteria. Subjects with AEs will be treated appropriately. Abnormal laboratory values not explained by the subjects' disease will be repeated until normal, or until the abnormality can be explained, and the subject's safety is not at risk.

9.8.2. SAE and SUSAR reporting

Should a SAE occur, the Principal Investigator (PI) will be informed and relationship to the study drug will be assessed by the PI using the definitions shown above. Complete information concerning the SAE will be collected by the PI and documented on a standard SAE form. SAE leading to death will be reported to the EC by the PI within 7 days. SUSARs leading to death or any other SUSARs will be reported to the EC within 7 and 15 days respectively.

9.9. Medical Follow-Up of Adverse Events

The Investigator will ensure the subject receives medical follow-up as necessary until the condition has stabilized or returned to normal state, even if the period of the trial is over.

9.10. Screening Procedures

9.10.1. Informed consent

For this study, the subject must be cognitively able to understand the requirements of the study and provide the informed consent to participate in the study; use of a legally authorized representative to provide consent to participate is not allowed. Informed consent forms (ICFs), any subsequently revised written ICFs, and any written information provided to the subject must receive Institutional Ethics Committee (IEC) approval in advance of use. The subject should be informed in a timely manner if new information becomes available that may be relevant to the subject's willingness to continue participation in the study. The communication of this information will be provided and documented via a revised main ICF or addendum to the original ICF that captures the subject's dated signature.
The ICFs will adhere to IEC requirements, applicable laws and regulations and Sponsor requirements.

9.10.2. Changes to protocol and informed consent forms

Study procedures will not be changed without the mutual agreement of the Investigator and Sponsor. If there are any substantial changes to the study protocol, these changes will be documented in a study protocol amendment and, where required, in a new version of the study protocol (Revised Protocol). The Sponsor will distribute any subsequent amendments and new versions of the protocol to the Investigator. If a protocol amendment requires a change to the ICF, the Sponsor and the EC should

approve the revised ICF before the revised form is used. If local regulations require, any administrative change will be communicated to or approved by the EC.

9.10.3. Physical health

Subjects will be examined by a study physician, including medical history, physical examination, ECG, vital signs, and laboratory blood tests at screening and at the end of study visit. Body weight and height will also be measured. Additionally, a psychiatric interview will be performed at the screening.

9.10.4. Mental health

Subjects will be screened using the MINI. The psychiatric interview is conducted by a trained site study staff who also decides whether subjects meet the psychiatric inclusion criteria. The C-SSRS will be conducted to screen for suicidality.

9.10.5. History of substance use and urine drug screens

The history of substance use will be recorded during the screening and baseline visits. Substance use in the past is not an exclusion criterion if no severe adverse reactions occurred to hallucinogens. Subjects will be asked to abstain from any illicit drug use and cannabis during the study.
At Screening (Visit 1), Baseline (Visit 2), and Day 1 (Visit 3), and at 2 visits randomly selected throughout the study, the urine dipstick drug screen will be performed locally to assess for illicit/prohibited drugs (see Section 8.2.1).
Positive urine drug screens (with the exception of THC and metabolites) at Screening or Baseline will result in termination from the study.

9.10.6. Laboratory tests

A routine laboratory blood test will be performed at the screening examination, and similarly during the treatment period and at the follow-up visit (see Schedule of Events); see Section 8.2. In addition, a urine drugs screen and a urine pregnancy test (only in women) are performed at select time points before IP dosing throughout the study; see Section 8.2.

9.10.7. Re-Screening

Screen failures are subjects who consent to participate in the clinical study but are never randomized. Subjects excluded during screening should promptly be reported as a screen failure in the electronic data capture (EDC) system.
Subjects excluded during screening may be rescreened once in consultation with the Sponsor’s Medical Monitor, when the reason for meeting an exclusion is likely to resolve (e.g., the subject had an exclusionary acute infection that is resolving, or a medication taper is taking longer than 28 days). If a subject is rescreened, the subject must be reconsented to participate in the study.

9.11. Urgent Safety Measures

All arrangements described in this section apply only to the extent that protocol requirements cannot be met because of restrictions due to a crisis, such as the COVID-19 pandemic. Study center visits should take place to the extent possible and usual protocol requirements adopted for all subjects as soon as the crisis-related limitations permit. Exceptional measures taken in response to a crisis (e.g., COVID-19) and their impact on study results, such as tests done in a different local laboratory, will be explained, assessed, and reported in the clinical study report following ICH E3 guidance.
In case any visit described in the Schedule of Events (Section 6.1) that is required to be conducted in-clinic and cannot be conducted at the study center within the visit window due to restrictions related to COVID-19 or another such crisis, a telephone visit, home visit and/or video call should be arranged. The telephone visit should include all protocol assessments that can be performed remotely and should take place within the original visit window. A home visit requires the PI or delegate to conduct all safety assessments and record AEs. A supportive person of the subject’s choosing may be present with the subject in the home. The decision to replace the study center visit with an alternate contact method will be made by the Investigator on a case-by-case basis, and the Sponsor’s Medical Monitor and clinical operations project manager should be informed of each case as early as possible. In addition to the remote visit, the Investigator should complete the following assessments/procedures:

- Outcome clinical assessments e.g., AISRS, CGI
- Concomitant medication review/collection
- Check daily diary
- Adverse event collection

- Safety laboratory assessment (if required at that visit)
- Urine pregnancy test (if required at that visit)
- Urine drug screen (if required at that visit)
- C-SSRS – SLV Version

For scheduled visits that require additional assessments such as clinical laboratory measurements, the use of local practitioners and resources may be employed. In case these assessments cannot be obtained within a reasonable time after the remote visit contact, the benefit-risk ratio for the subject to continue in the study should be reassessed.

All safety data that are possible to obtain locally should be collected at the remote visit.

9.12. Special Situations

Special situations with study drug are defined as the following:

- Medication error or incorrect drug administration
- Overdose (exceeds the protocol-specified maximum; see Section 9.4 for the definition of overdose)
- Deliberate abuse
- Deliberate misuse
- Drug interaction
- Occupational exposure

If any of these special situations with study drug occur, they will be reported on the special situations with study drug eCRF page, even if there is no accompanying AE. All clinical manifestations in relation to these special situations will be reported as AEs or SAEs at the same time using the corresponding section of the eCRF.

For example, if a medication error or interaction occurred, the Investigator will assess whether to consider an AE to have occurred, and if so, will assign the possible relationship to the study drug. Additional information may be requested by the site including all AE information and all triggering drug accountability discrepancies.

10. COLLECTION OF STUDY VARIABLES

The study assessments are described in the sections below and the timing of these assessments are detailed in the Schedule of Events (Section 6.1).

10.1. Recording of Data

For this study, subject data will be collected on electronic Case Report Forms (eCRFs). The Investigator will ensure the accuracy, completeness, and timeliness of the data recorded, data queries, and all required reports according to any instructions provided in the eCRF completion guidelines.

10.2. Clinical Laboratory Assessment

The laboratory parameters listed below will be measured:

**Standard Chemistry:**	
Albumin	Chloride
Total protein	Creatinine
Alkaline phosphatase	Creatinine clearance (CKD-EPI)
ALT	FSH (to confirm postmenopausal status)
AST	GGT
Bilirubin, total (+ direct if total is >ULN)	Glucose
Blood urea nitrogen or urea	Lactate dehydrogenase
Calcium	Phosphate
Sodium	Potassium

**Hematology complete blood count, including:**<br>Hemoglobin<br>Hematocrit<br>● MCV, MCH<br>● Platelet count<br>● Red blood cell count<br>● White blood cell count<br>**Differential count if WBC is elevated, including:**<br>● Basophils, Eosinophils, Lymphocytes, Monocytes and Neutrophils
**Urine pregnancy test**
**Urine drug screening**

A urine dipstick drug screen will be used during the screening process (to identify the use of drugs of abuse/illicit drugs and/or certain prohibited medications) and at later timepoints in the study (to identify the use of cannabis, which might confound the results of the study).

The visits where a urine dipstick drug screen is performed are outlined in the Schedule of Events (Table 2). The results of the urine dipstick drug screen will be recorded in the eCRF. The urine drug screening may be performed at additional timepoints per the Investigator's discretion, and the reason for the additional testing should be recorded in the eCRF.

At specified visits, the urine dipstick drug screen will be performed locally to assess for the following drugs: Amphetamines (amphetamine, MDMA, methamphetamine), barbiturates, benzodiazepines, cocaine metabolite, opioids (morphine, methadone, buprenorphine, oxycodone, heroine metabolite), phencyclidine (PCP), tricyclic antidepressants, THC metabolite. No reflex testing is required for a positive result; retesting may be performed for an indeterminate result. The site staff/Investigator should confer with the Sponsor's Medical Monitor or designee if there are any questions about a positive result.

For all random urine drug tests through End of Study or Early Termination, the urine dipstick drug screen is performed locally; if positive or indeterminate result, a sample may be sent to the site's local laboratory for **confirmatory testing**.

10.2.1. Physical examination

A physical examination will be performed at screening, Week 6, and at Week 10 visits and include an assessment of the following: general appearance, skin, head and neck, lymph nodes, thyroid, abdomen, musculoskeletal, cardiovascular, respiratory, and neurological systems. An assessment for any signs or symptoms of skin irritation will be noted in source and eCRF.

Height will be measured in centimeters and weight in kilograms. Measurements should be taken without shoes for all measurements. Body mass index (BMI) will be calculated from the height and weight measurements at screening.

10.2.2. Resting 12-Lead ECG

A 12-lead ECG will be obtained after the subject has been resting in the supine position for at least 5 minutes, at time points outlined in the Schedule of Events. All ECGs will be documented by recording date, time of collection, all abnormal waveform noted, heart rate, all intervals including PR, RR, QRS, QT, and QTcF/QTcB intervals from the 12-lead ECG. The Investigator will judge the overall interpretation as normal or abnormal. If abnormal, it will be decided whether or not the abnormality is clinically significant or not clinically significant, and the reason for the abnormality will be recorded. The date/time and the physician interpretation (normal, abnormal clinically significant, abnormal not clinically significant) for the paper ECGs will be stored as source documents as a paper printout. Skin preparation must be thorough and electrode positions must be according to standard 12-lead ECG placement.

10.2.3. Vital signs

Vital signs will be measured according to the Schedule of Events. Vital signs should be obtained after the subject has been resting in a supine position for at least 5 minutes.

10.2.4. Supine pulse and blood pressure

Blood pressure will be measured using a semiautomatic blood pressure recording device with an appropriate cuff size. Subjects will be required to rest in a supine position for at least 5 minutes prior to taking blood pressure, and pulse rate measurements.

10.2.5. Body temperature

Body temperature (tympanic) will be measured in degrees of Celsius using an automated thermometer. No hot/cold beverage/food is allowed for 15 minutes before the temperature is measured.

10.2.6. Emergency treatment

During and following a subject's participation in the trial, the Investigator/institution should ensure that adequate medical care is provided to a subject for any AEs, including clinically significant laboratory values, related to the trial.

10.3. Pharmacokinetics

10.3.1. Collection of pharmacokinetic samples

The timing and number of PK samples will be at 0, 0.5, 1, 2, 3, 4 and 6 hours. The actual date and time of collection of each sample will be recorded on the eCRF. Samples will be collected (aliquot and back-up) to be stored at -20°C (for the back-up) and -80°C (for the aliquot). Blood samples will be collected in heparinized/EDTA tubes, centrifuged at 4°C at 3000 rpm for 10 min, and the plasma is then stored at -20°C until shipping/analysis.

10.4. Pharmacodynamics

The acute subjective effects of MM-120 and placebo will be assessed for 6 hours after administration on Day 1 and after the last dose, using the 5 Dimensions of Altered States of Consciousness Questionnaire (5D-ASC), and the Mystical Experience Questionnaire 30 item (MEQ30). The 5D-ASC and MEQ30 will also be self-administered at home on 6 hours after dosing on the first and last dosing days. Additionally, a series of single item Visual Analog Scales (VAS) will be used to self-rate acute drug effects repeatedly 0-6 hours after drug administration of the first dose of IMP.

10.4.1. 5D-ASC

The 5D-ASC scale is a visual analog scale consisting of 94 items (Dittrich, 1998; Studerus et al., 2010). The instrument is constructed of five scales, and allows assessing mood, anxiety, derealization, depersonalization, changes in perception, auditory alterations, and reduced vigilance. The scale is well-validated and has been used to characterize the acute subjective effects of LSD in experimental studies. The 5D-ASC scale will be administered 6 h after dosing of the first and last dose of MM-120, and subjects are instructed to retrospectively rate peak alterations. Each item of the scale is scored on a 0-100 mm VAS. Subjects will complete this assessment at home.

10.4.2. MEQ30

This 30-item questionnaire is rated on a six-point scale. The scale has been used to assess mystical experiences in studies using psilocybin and LSD. The MEQ30 will be administered 6 hours after drug administration of the first and last dose of MM-120, and subjects are instructed to retrospectively rate peak alterations. Data on each domain scale will be expressed as a percentage of the maximum possible score. Subjects will complete this assessment at home.

10.4.3. 8.4.3. VAS

A series of single item VAS will be used repeatedly 0-6 hours after the first drug administration. The following VAS items will be used: "any drug effect", "good drug effect", bad drug effect", "drug liking", "fear", nausea", "alteration of vision", "alteration of sense of time", and "the boundaries between myself and my surroundings seem to blur".

11. BIOLOGICAL SAMPLING PROCEDURES

11.1. Volume of Blood

Blood samples for determination of plasma concentration of MM-120 will be collected pre- and post-dose on Day 1. The sampling scheme and sampling duration, without exceeding the maximal sample number, duration, or maximal blood volume in this protocol, will be maintained.

The number of samples to be obtained, as well as the volume required for each analysis, may be changed during the study as new data become available. However, the maximum volume to be drawn from each subject will be approximately 120 mL.

11.2. Handling, Storage and Destruction of Biological Samples

The samples will be used or disposed of after analyses.

11.2.1. Quality control of PK samples

Additional analyses will be conducted on the biological samples to investigate the reproducibility of the analytical results in incurred samples. Any results from such analyses will only be used to confirm the reproducibility of the method and will be reported in a separate table in the bioanalytical study contribution report.

12. ETHICAL AND REGULATORY REQUIREMENTS

12.1. Ethical Conduct of Study

The study will be performed in accordance with ethical principles that have their origin in the Declaration of Helsinki and are consistent with International Conference on Harmonization (ICH) Good Clinical Practice (GCP).

12.2. Subject Data Protection

The informed consent form (ICF) will incorporate (or, in some cases, be accompanied by a separate document incorporating) wording that complies with relevant data protection and privacy legislation.

12.3. Ethics and Regulatory Review

This study will be conducted in compliance with the protocol, the current version of the Declaration of Helsinki (9th July 2018), the ICH-GCP as well as all national legal and regulatory requirements. The Ethics Committees (ECs) should approve the final study protocol, including the final version of the ICF and any other written information and/or materials to be provided to the subjects. The Investigators will ensure the distribution of these documents to the applicable EC, and to the study site staff. The opinion of the EC should be given in writing. The Investigator should submit the written approval to the Sponsor before enrolment of any subject into the study.
The EC should approve all advertising used to recruit subjects for the study.
The Sponsor will approve any modifications to the ICF that are needed to meet local requirements.
The IB will be updated and reviewed annually by the Sponsor.

12.4. Informed Consent

Any provisions for subjects harmed as a consequence of study participation should be described in the ICF that is approved by an EC.
The Investigator will:

- Ensure that each subject is given full and adequate oral and written information about the nature, purpose, possible risk, and benefit of the study.
- Ensure that each subject is notified that they are free to withdraw from the study at any time.
- Ensure that each subject is given the opportunity to ask questions and allowed time to consider the information provided.
- Ensure each subject provides signed and dated informed consent before conducting any procedure specifically, for the study.
- Ensure the original, signed ICF is stored in the Investigator’s Study File (ISF).
- Ensure copies of the signed ICF are given to the subject.

12.5. Changes to Protocol and Informed Consent Forms

Study procedures will not be changed without the mutual agreement of the Investigator and Sponsor. If there are any substantial changes to the study protocol, these changes will be documented in a study protocol amendment and, where required, in a new version of the study protocol (Revised Protocol). The Sponsor will distribute any subsequent amendments and new versions of the protocol to the Investigator. If a protocol amendment requires a change to the ICF, the Sponsor and the EC should approve the revised ICF before the revised form is used. If local regulations require, any administrative change will be communicated to or approved by the EC.

12.6. Audits and Inspections

Authorized representatives of the Sponsor, Health Authority, or an EC may perform audits or inspections at the center, including source data verification. The purpose of an audit or inspection is to systematically and independently examine all study-related activities and documents to determine whether these activities were conducted, and data were recorded, analyzed, and accurately reported according to the protocol, GCP, guidelines of the ICH, and any applicable regulatory requirements. The Investigator will contact the Sponsor immediately if contacted by a regulatory agency about an inspection at the center.

13. STUDY MANAGEMENT

13.1. Pre-study Activities

Before the first subject is entered into the study, it is necessary for a representative of the Sponsor, or their delegate, to visit the investigational study site to:

- Determine the adequacy of the facilities
- Determine availability of subjects appropriate for the study
- Discuss with the Investigator (and other personnel involved with the study) their responsibilities with regards to protocol adherence, and the responsibilities of the Sponsor or its representatives. This will be documented in a Clinical Study Agreement between the Sponsor and the Investigator.

13.1.1. Training of Study Site Personnel

Before the first subject is entered into the study, a Sponsor's representative will review and discuss the requirements of the study protocol and related documents with the investigational staff, and train them in any study-specific procedures and system(s) utilized.
The Investigator will ensure that appropriate training relevant to the study is given to all staff, and that any new information relevant to the performance of this study is forwarded to the staff involved.
The Investigator will maintain a record of all individuals involved in the study (medical, nursing, and other staff).

13.1.2. Monitoring of Study

During the study, a representative of the Sponsor will have regular contact with the study site for the following reasons:

- To conduct visits to provide information and support to the Investigator,
- To confirm that facilities remain acceptable,
- To confirm o that the investigational team is adhering to the protocol, o that data are being accurately and timely recorded in the eCRFs, o that biological samples are handled correctly, and o that IMP accountability checks are being performed,
- To perform source data verification (a comparison of the data in the electronic data capture [EDC] system with the subject's medical records at the hospital or practice, and other records relevant to the study) including verification of informed consent of participating subjects. o Note that this will require direct access to all original records for each subject
- To ensure that any subject's withdrawal of informed consent to use of their biological samples is correctly reported and that these biological samples are identified and disposed of/destroyed accordingly, and the action is documented and reported to the subject.

The Sponsor's representative will be available between visits if the Investigator or other staff at the site needs information and advice about the study conduct.
Each site will comply with applicable laws per each regulatory authority. The Investigator is responsible for ensuring compliance at the site with all procedures and details of drug handling, storage and accountability for dosing, tracking, control and return via reverse distributor, and prevention of unauthorized access.

13.1.3. Study agreements

The Investigator should comply with all the terms, conditions, and obligations of the Clinical Study Agreement for this study. In the event of any inconsistency between this study protocol and the Clinical Study Agreement, the study protocol shall prevail with respect to the conduct of the study and

the treatment of subjects and in all other respects, the terms of the Clinical Study Agreement shall prevail. Agreements between the Sponsor and the Investigator should be in place before any study-related procedures can take place or subjects are enrolled.

13.1.4. Archiving of study documents

The Investigator follows the principles outlined in the Clinical Study Agreement.

13.1.5. Study termination

The study may be terminated if the study procedures are not being performed according to GCP, or according to the protocol, or if recruitment is slow. The Sponsor may also terminate the entire study prematurely if concerns for safety arise within this study.

13.2. Data Management by a Delegate

Data management will be performed by the Sponsor or its delegate. Data queries will be raised for inconsistent, impossible, or missing data. All entries to the study database will be available in an audit trail. The data will be validated as defined in the Data Management Plan. Quality control procedures will be applied to each stage of data handling to ensure that all data are reliable and have been processed correctly. When all data have been coded, validated, and locked, a clean file will be declared. Any treatment-revealing data may thereafter be added, and the final database will be locked. Data storage will be done in accordance with GCP guidelines. The Data Management Plan will describe the methods used to collect, check, and process clinical data in detail. It will also clarify the roles and responsibilities for the different functions and personnel involved in the data management process. Furthermore, the Data Management Plan will describe the data flow and timelines within the study.

14. EVALUATION AND CALCULATION OF VARIABLES

14.1. Calculation or Derivation of Safety Variable(s)

The verbatim terms used in the eCRF by investigators to identify adverse events will be coded using the Medical Dictionary for Regulatory Activities (MedDRA). Intervention-emergent adverse events are adverse events with onset after the intervention phase or that are a consequence of a pre-existing condition that has worsened since treatment start. All reported adverse events will be included in the analysis. For each adverse event, the percentage of subjects who experience at least one occurrence of the given event will be summarized by treatment arm. In addition, comparisons between intervention groups will be provided as appropriate.
Summaries, listings, datasets, or subject narratives may be provided, as appropriate, for those subjects who die, or who experience a severe or a serious adverse event.
Safety outcomes, including vital signs, laboratory parameters' values, ECG parameters and abnormalities, will be reported by treatment arm. All safety data (scheduled and unscheduled) will be presented in the data listings. Continuous variables will be summarized using descriptive statistics (n, mean, SD, minimum, median, and maximum) by treatment/dose group at relevant timepoints. Categorical variables will be summarized in frequency tables (frequency and proportion) by treatment/dose group at relevant timepoints. Graphical presentations will be used as appropriate. Where applicable, data will be summarized for the absolute value at each scheduled assessment and for the corresponding change from baseline. For clinical laboratory tests, listings of values for each participant will be presented with abnormal or out-of-range values flagged. The percentage of subjects with values beyond clinically important limits will be summarized by frequency tables and shift tables. Treatment emergent ECG abnormalities will be summarized similarly to AEs.

14.2. Other Significant Adverse Events

During the evaluation of the AE data, the Sponsor’s medically qualified expert will review the list of AEs that were not reported as SAEs or AEs leading to discontinuation. Based on the expert’s judgment, significant AEs of particular clinical importance may be considered other significant adverse events (OAEs) and reported as such in the Clinical Study Report (CSR). A similar review of other data from laboratory tests, vital signs, ECGs, and other safety assessments will be performed for identification of OAEs. Examples of these are marked hematological and other laboratory abnormalities, certain events that lead to intervention (other than those already classified as serious), or significant additional treatment.

15. STATISTICAL METHODS AND SAMPLE SIZE

15.1. Data Analysis

The statistical analysis will be done by the Sponsor or under the authority of the Sponsor. A general description of the statistical methods to be used to analyze the efficacy and safety data is outlined below. Specific details will be provided in the Statistical Analysis Plan (SAP) that will be finalized prior to unblinding.

15.2. Analysis Sets

The following analysis sets will be defined for the statistical analysis. Subjects without valid written study informed consent will be excluded from all analysis sets.

- **Randomized set (RAN)** – All subjects who received a randomization number, regardless of receiving trial medication.
- **Safety set (SAF)** – All subjects who received the double-blind study drug. Subjects will be analyzed according to treatment received.
- **Full analysis set (FAS)** – All subjects in RAN who were not mis-randomized
  - † Following the intent-to-treat (ITT) principle, subjects are analyzed according to the treatment they have been assigned to at the randomization.
  - † Mis-randomized subjects are those who have not been qualified for randomization and who have been inadvertently randomized into the study but have not received doubleblind study drug.
- **Per protocol set (PPS)** – All subjects in FAS who took the study medication and had no major protocol deviations
- **Pharmacokinetic analysis set (PKAS)** –The PK analysis set will include all participants who receive a dose of MM-120 and have at least 1 postdose PK measurement without important protocol deviations or violations thought to significantly affect the PK of the drug. Data from participants with deviations determined to affect PK will be excluded from the PK analysis set. Participants that receive placebo will not be part of the PK analysis set. A strategy for dealing with data affected by protocol violations and deviations will be agreed to by the Sponsor, monitor, and pharmacokineticist, prior to clean file and code break. Participants will be analyzed according to the treatment they actually received.
  - ‡ Subjects will be analyzed according to the treatment received.
  - ‡ Major protocol deviations are those affecting the primary endpoint analyses and will be finalized prior to unblinding treatment codes for analyses. A list will be provided in the Clinical Study Report Statistical Analysis Plan.

15.3. Subject demographics and other baseline characteristics

Summary statistics will be provided by treatment group for demographics and baseline characteristics, including sex, age, weight, height, BMI, systolic blood pressure, diastolic blood pressure, geographical region, medical history, baseline values for AISRS, CGI-S, ASRS, and CAARS. These summaries will be performed for the FAS.

Continuous variables will be summarized using n, mean, standard deviation (SD), median, Q1 (25th percentile), Q3 (75th percentile), minimum, and maximum. Categorical variables will be summarized using frequency and incidence.

15.4. Treatment Exposure

Exposure to randomized treatment for the SAF set during the double-blind treatment period will be summarized in terms of treatment duration, calculated as the number of days from the date of first dose of randomized treatment received to the date of the last dose received, inclusive. Descriptive statistics (n, mean, SD, minimum, median, and maximum) will be presented by treatment group for treatment exposure. The total number of doses received by a subject during the double-blind treatment period will be summarized by treatment group for the SAF.

15.5. Prior and Concomitant Medication

Prior medication is defined as any medication taken before the date of the first dose of randomized treatment. Concomitant medication is defined as any medication taken on or after the date of the first dose of randomized treatment.

Both prior and concomitant medication use will be summarized by the number and proportion of subjects in each treatment group receiving each medication within each therapeutic class for the SAF.

15.6. Efficacy Analyses

15.6.1. Primary Estimand

The primary estimand quantifies the treatment effect of MM-120 at 6-weeks vs placebo after twice weekly administration while accounting for intercurrent events (IEs) with potential confounding effects and IEs leading to study discontinuation prior to the 6-week assessment.

The following attributes describe the primary estimand:

- Population: Subjects in the FAS
- Endpoint: Change from baseline to Week 6 in the AISRS Score
- Treatment of interest: the randomized treatment MM-120 (LSD) or placebo as a twice weekly treatment.
- Summary measure: Difference between MM-120 and placebo in mean change from baseline to Week 6 for AISRS Score.

15.6.2. Handling of missing/potentially biased values

Missing/potentially biased data will be imputed using a multiple imputation approach assuming that the missingness mechanism can be retrieved from observed data. The imputation model will include the longitudinal sequence of AISRS Scores, and the covariates used in the model as appropriate. Depending on the Intercurrent Event (IE) causing the missing or potentially biased value, two sequential policy strategies will be applied.

Strategy 1; missing at random (MAR), for:

- IEs related to COVID-19 pandemic and other intercurrent missing data,

Missing data will be imputed under a MAR assumption, borrowing information from subjects in the same treatment arm.

Strategy 2; missing not at random (MNAR) for:

- Intake or change in concomitant medications/therapies which have potential confounding effects,
- Intake of prohibited medications/therapies,
- Other IEs leading to treatment/study discontinuation (except those related to COVID-19 pandemic,

The data recorded after the IE will be imputed/substituted under a MNAR assumption by borrowing information from the placebo arm subjects (reference-based imputation).

15.6.3. Analysis details

The primary analysis will be performed according to the intent-to-treat (ITT) principle on the FAS. The effect on the primary endpoint, change in AISRS score from baseline to Week 6, will be analyzed applying Rubin's rule to a multiple imputations procedure to appropriate contrasts based on Mixed Model for Repeated Measures (MMRM) analysis of covariance with the change in the AISRS score from baseline to Week 2 and Week 6 visits as dependent variable, and treatment group, visit, visit-by treatment group interaction, baseline score of AISRS, sex and age as fixed effects. An unstructured variance-covariance matrix will be used to model within-subject errors. Satterthwaite's method will be used to approximate the degrees of freedom. If convergence problems arise (even just in one imputed dataset) the following order of matrices will be applied: Heterogeneous TOEP (TOEPH), Toeplitz (TOEP), Autoregressive(1) (AR(1)). The same variance-covariance matrix will be used in sensitivity analyses.

The imputation model includes the longitudinal sequence of AISRS Scores, and the covariates used in the model as defined above. As a general approach, imputations are performed on observed values specifying a minimum and a maximum threshold (zero and 54 respectively) for AISRS. The change from baseline in AISRS Scores will not be imputed to avoid imputation of nonsensical values. For the imputation of missing data of AISRS, one thousand (1000) data sets will be generated, and the random seed number will be 200208. MI will be implemented following the steps below:

1. Imputation assuming a MAR (strategy 1).
   MAR will be applied following the steps below:
   a) all assessments that took place after the IEs will be kept in the database;
   b) imputing all missing values already present in the data.
   MI will be carried out based on fully conditional specification method (FCS). These datasets will be utilized in Step #2.
2. Imputation assuming MNAR (strategy 2). MNAR will be applied following the steps below:
   a) assigning missing values to all assessments that took place after the IEs specified in policy strategy 2 above;
   imputing missing values based on copy reference approach (i.e. using the non-missing values of the placebo arm) will be used.
3. The MMRM model defined above will be run on each of the 1000 generated datasets with observed and imputed data. Rubin's rule will be used for combining results to draw inference.

In the evaluation of the primary endpoint will be included only subjects with NON missing baseline assessments.

The LS Means along with associated standard errors (SE) and 95%CIs will be displayed for each treatment group at each time point. For the comparison of MM-120 (LSD) vs placebo treatment effect, the LS Mean, SE, 95%CI and one-sided p-value will be provided.

A listing of AISRS values overtime will be provided for the FAS population.

15.6.4. Sensitivity analyses

Sensitivity analyses will be implemented to assess the robustness of results on primary endpoint versus assumptions used in the statistical model for the main estimator.
The following sensitivity analyses will be performed:

- Main analysis on the PPS.
- Main analysis stratified by ADHD severity at baseline.
- Main analysis with an MAR only imputation approach.
- Main analysis without any imputations.

15.6.5. Sample size determination

Randomizing 26 subjects per arm, if the true efficacy of MM-120 reaches a standardized mean improvement over placebo of 0.60, the null hypothesis that active is not better than placebo will be rejected with a one-sided p-value < 0.10 with 80% power.
The minimum estimated standardized effect size resulting in a p-value < 0.10 is 0.38 while an estimated standardized effect size < 0.56 will result in a one-sided p-value < 0.025.

15.6.6. Secondary efficacy endpoints

15.6.6.1. Key secondary efficacy endpoint

The change from baseline to Week 1 in AISRS Score will be analyzed using the same process as the primary endpoint. The outcome will be considered confirmatory, if in agreement with the primary endpoint outcome and provided that the null hypothesis is rejected for the primary outcome. This gatekeeping function of the primary outcome allows to avoid any adjustment for multiplicity of testing.

15.6.6.2. Other secondary and exploratory efficacy endpoints

Change from baseline to different timepoints in AISRS, CGI-S, ASRS, and CAARS, as well as sleep quality score and duration, will be analyzed descriptively reporting by treatment: n, mean, standard deviation, Q1 (25th percentile), Q3 (75th percentile), minimum, and maximum, and 95% Confidence Interval for the mean and for the median. Similar statistics will be reported for the difference from placebo. The occurrence of 1-point improvement from baseline to Week 1 and 6 in CGI-S will be

reported by a frequency table with proportions in each arm and by proportions' difference and ratio with relevant 95% Confidence Intervals.
For the sleep quality score and sleep duration the endpoint will be measured as the change from the mean of the 7 days preceding treatment to the 7 days preceding the relevant visit (Weeks 1 and 6).

15.6.7. Subgroup analyses

Analyses of the primary/secondary endpoints within subpopulations will be performed with exploratory purpose.

15.7. Safety analyses

No statistical inference on safety parameters is planned.

15.7.1. Adverse events

The verbatim terms used in the eCRF by investigators to identify adverse events will be coded using the Medical Dictionary for Regulatory Activities (MedDRA). Intervention-emergent adverse events are adverse events with onset after the intervention phase or that are a consequence of a pre-existing condition that has worsened since treatment start. All reported adverse events will be included in the analysis. For each adverse event, the percentage of subjects who experience at least one occurrence of the given event will be summarized by treatment arm. In addition, comparisons between intervention groups will be provided as appropriate.
Summaries, listings, datasets, or subject narratives may be provided, as appropriate, for those subjects who die, or who experience a severe or a serious adverse event.

15.7.2. Other safety parameters

Safety outcomes, including vital signs, laboratory parameters' values, ECG parameters and abnormalities, will be reported by treatment arm. All safety data (scheduled and unscheduled) will be presented in the data listings. Continuous variables will be summarized using descriptive statistics (n, mean, SD, minimum, median, and maximum) by treatment/dose group at relevant timepoints. Categorical variables will be summarized in frequency tables (frequency and proportion) by treatment/dose group at relevant timepoints. Graphical presentations will be used as appropriate. Where applicable, data will be summarized for the absolute value at each scheduled assessment and for the corresponding change from baseline. For clinical laboratory tests, listings of values for each subject will be presented with abnormal or out-of-range values flagged. The percentage of subjects with values beyond clinically important limits will be summarized by frequency tables and shift tables. Treatment emergent ECG abnormalities will be summarized similarly to AEs.

15.8. Pharmacodynamics

Pharmacodynamics data will be analyzed descriptively reporting by dose: n, mean, standard deviation, Q1 (25th percentile), Q3 (75th percentile), minimum, and maximum, and 95% Confidence Interval for the mean and for the median.

16. IMPORTANT MEDICAL PROCEDURES TO BE FOLLOWED BY THE INVESTIGATOR

16.1. Medical Emergencies and Contacts

The Investigator is responsible for ensuring that procedures and expertise are available to handle medical emergencies during the study. A medical emergency usually constitutes a SAE and is to be reported as such. In the case of a medical emergency, the Investigator may contact the Sponsor’s physician.

16.2. Overdose

In the event of an overdosage, the subject should be monitored closely and treated symptomatically. Use of IMP in doses in excess of that specified in the protocol should not be recorded in the eCRF system as an AE of "Overdose” unless there are associated symptoms or signs. The associated symptoms or signs will be the AE terms documented in the source documents. An overdose with associated SAE(s) must be recorded as the SAE diagnosis/symptoms on the relevant AE forms in the source documentation and eCRF only. An overdose with associated nonserious AEs must be recorded as the AE diagnosis/symptoms on the relevant AE forms in the source documentation and in the eCRF. Only overdoses of IMP will be reported.

16.3. Pregnancy

All pregnancies and their subsequent outcome (spontaneous miscarriage, elective termination, ectopic pregnancy, normal birth, or congenital abnormality) should be reported to the Sponsor. Pregnancy is not considered to be an SAE.

16.4. Maternal Exposure

Women of childbearing potential must use an effective means of birth control (e.g., doublebarrier method). Should a pregnancy still occur, it should be reported to the Sponsor's Medical Monitor and to the local EC as required. If a subject becomes pregnant during the study from Day 1/Dosing Session through Week 6, she is required to withdraw from the study. If a female sexual partner of a male subject becomes pregnant (by the male subject) within Day 1/Dosing
Session through Week 6, and if the subject agrees, the subject's pregnant partner should be notified. A completed ICF allows release of information, permitting transfer of information regarding the pregnancy and outcome to the Sponsor's Medical Monitor. The Investigator must inform the subject of their right to receive treatment information. If the pregnant subject (or pregnant female partner of the subject) chooses to receive unblinded treatment information, the individual blind should be broken, and the treatment assignment provided to the subject/subject's partner. The study team will remain blinded to the subject's (partner's) treatment assignment. If she agrees, the Investigator should notify the subject's (partner's) primary care physician of the pregnancy and provide details of the subject's participation in the study and treatment (blinded or unblinded, as applicable).
Pregnancy is to be reported to the Sponsor's Medical Monitor and drug safety and pharmacovigilance (PV) within 24 hours of awareness by the study site personnel, using the pregnancy reporting forms and the contact information in Section 14.1. The expected date of delivery or expected date of the end of the pregnancy, last menstruation, estimated conception date, pregnancy result, neonatal data, etc. should be included in this information, as available. The Investigator will follow the medical status of the mother, as well as the fetus, and will report the outcome to the Sponsor's Medical Monitor.

16.5. Paternal Exposure

Pregnancy of a subject's partner is not considered to be an SAE. However, any conception occurring from the date of dosing until 3 months after dosing should be reported to the Sponsor and followed.

17. REFERENCES

- Akimoto H, Oshima S, Sugiyama T, Negishi A, Nemoto T, & Kobayashi D (2019). Changes in brain metabolites related to stress resilience: Metabolomic analysis of the hippocampus in a rat model of depression. Behav Brain Res 359: 342-352.
- Anderson T, Petranker R, Christopher A, Rosenbaum D, Weissman C, Dinh-Williams LA, et al. (2019a). Psychedelic microdosing benefits and challenges: an empirical codebook. Harm Reduct J 16: 43.
- Anderson T, Petranker R, Rosenbaum D, Weissman CR, Dinh-Williams LA, Hui K, et al. (2019b). Microdosing psychedelics: personality, mental health, and creativity differences in microdosers. Psychopharmacology 236: 731-740.
- Baumeister D, Barnes G, Giaroli G, & Tracy D (2014). Classical hallucinogens as antidepressants? A review of pharmacodynamics and putative clinical roles. Ther Adv Psychopharmacol 4: 156-169.
- Belleville RE, Fraser HF, Isbell H, Wikler A, & Logan CR (1956). Studies on lysergic acid diethylamide (LSD-25): I. Effects in former morphine addicts and development of tolerance during chronic intoxication. AMA Arch Neurol Psychiatry 76: 468-478.
- Bercel NA, Travis LE, Olinger LB, & Dreikurs E (1956). Model psychoses induced by LSD25 in normals. I. Psychophysiological investigations, with special reference to the mechanism of the paranoid reaction. AMA Arch Neurol Psychiatry 75: 588-611.
- Bershad AK, Schepers ST, Bremmer MP, Lee R, & de Wit H (2019). Acute Subjective and Behavioral Effects of Microdoses of Lysergic Acid Diethylamide in Healthy Human Volunteers. Biol Psychiatry 86: 792-800.
- Biederman J, Faraone SV, Spencer TJ, Mick E, Monuteaux MC, & Aleardi M (2006a). Functional impairments in adults with self-reports of diagnosed ADHD: A controlled study of 1001 adults in the community. The Journal of clinical psychiatry 67: 524-540.

- Biederman J, Monuteaux MC, Mick E, Spencer T, Wilens TE, Silva JM, et al. (2006b). Young adult outcome of attention deficit hyperactivity disorder: a controlled 10-year follow-up study. Psychol Med 36: 167-179.
  Bogenschutz MP, Forcehimes AA, Pommy JA, Wilcox CE, Barbosa PC, & Strassman RJ (2015). Psilocybin- assisted treatment for alcohol dependence: a proof-of-concept study. J Psychopharmacol 29: 289-299.
- Carhart-Harris RL, Bolstridge M, Day CMJ, Rucker J, Watts R, Erritzoe DE, et al. (2017).
- Psilocybin with psychological support for treatment-resistant depression: six-month follow-up. Psychopharmacology.
- Carhart-Harris RL, Bolstridge M, Rucker J, Day CM, Erritzoe D, Kaelen M, et al. (2016a). Psilocybin with psychological support for treatment-resistant depression: an open-label feasibility study. Lancet Psychiatry 3: 619-627.
- Carhart-Harris RL, Kaelen M, Bolstridge M, Williams TM, Williams LT, Underwood R, et al. (2016b). The paradoxical psychological effects of lysergic acid diethylamide (LSD). Psychol Med 46: 1379-1390.
- Carhart-Harris RL, Kaelen M, Whalley MG, Bolstridge M, Feilding A, & Nutt DJ (2015). LSD enhances suggestibility in healthy volunteers. Psychopharmacology 232: 785-794.
- Carhart-Harris RL, Muthukumaraswamy S, Roseman L, Kaelen M, Droog W, Murphy K, et al. (2016c). Neural correlates of the LSD experience revealed by multimodal neuroimaging. Proc Natl Acad Sci U S A 113: 4853- 4858.
- Castle L, Aubert RE, Verbrugge RR, Khalid M, & Epstein RS (2007). Trends in medication treatment for ADHD. Journal of attention disorders 10: 335-342.
- Davenport WJ (2016). Psychedelic and nonpsychedelic LSD and psilocybin for cluster headache. CMAJ 188: 217.
- Dinges DF, & Powell JW (1985). Microcomputer analyses of performance on a portable, simple visual RT task during sustained operations. Behav Res Methods Instruments Computers 17: 652-655.
- Dittrich A (1998). The standardized psychometric assessment of altered states of consciousness (ASCs) in humans. Pharmacopsychiatry 31 (Suppl 2): 80-84.
- Dolder PC, Holze F, Liakoni E, Harder S, Schmid Y, & Liechti ME (2017a). Alcohol acutely enhances decoding of positive emotions and emotional concern for positive stimuli and facilitates the viewing of sexual images. Psychopharmacology 234: 41-51.
- Dolder PC, Liechti ME, & Rentsch KM (2015a). Development and validation of a rapid turboflow LC-MS/MS method for the quantification of LSD and 2-oxo-3-hydroxy LSD in serum and urine samples of emergency toxicological cases. Anal Bioanal Chem 407: 15771584.
- Dolder PC, Liechti ME, & Rentsch KM (2018). Development and validation of an LC-MS/MS method to quantify lysergic acid diethylamide (LSD), iso-LSD, 2-oxo-3-hydroxy-LSD, and nor-LSD and identify novel metabolites in plasma samples in a controlled clinical trial. J Clin Lab Anal 32: e22265.
- Dolder PC, Schmid Y, Haschke M, Rentsch KM, & Liechti ME (2015b). Pharmacokinetics and concentration- effect relationship of oral LSD in humans. Int J Neuropsychopharmacol 19: pii: pyv072.
- Dolder PC, Schmid Y, Mueller F, Borgwardt S, & Liechti ME (2016). LSD acutely impairs fear recognition and enhances emotional empathy and sociality. Neuropsychopharmacology 41: 2638-2646.
- Dolder PC, Schmid Y, Steuer AE, Kraemer T, Rentsch KM, Hammann F, et al. (2017b).
- Pharmacokinetics and pharmacodynamics of lysergic acid diethylamide in healthy patients.
- Clin Pharmacokinetics 56: 1219-1230.
- Eakin L, Minde K, Hechtman L, Ochs E, Krane E, Bouffard R, et al. (2004). The marital and family functioning of adults with ADHD and their spouses. Journal of attention disorders 8: 110.
- Einsatz bewusstseinsverändernden Substanzen zu therapeutischen Zwecken. [Online] Available from http://www.psychosomatikbasel.ch/deutsch/bildung/dienstagmittagfortbildung/pdf/2012/gasser220512p.pdf. [Accessed].
- EMCDDA (2016). European Drug Report 2016. European Monitoring Center for Drugs and Drug Addiction (EMCDDA) available at www.emcdda.europa.eu.

- Fadiman J, & Korb S (2019). Might Microdosing Psychedelics Be Safe and Beneficial? An Initial Exploration. J Psychoactive Drugs 51: 118-122.
- Family N, Maillet EL, Williams LTJ, Krediet E, Carhart-Harris RL, Williams TM, et al. (2020). Safety, tolerability, pharmacokinetics, and pharmacodynamics of low dose lysergic acid diethylamide (LSD) in healthy older volunteers. Psychopharmacology 237: 841-853.
- Fillmore MT, Rush CR, & Hays L (2002). Acute effects of oral cocaine on inhibitory control of behavior in humans. Drug and alcohol dependence 67: 157-167.
- Fischman MW, & Johanson CE (1998). Ethical and practical issues involved in behavioral pharmacology research that administers drugs of abuse to human volunteers. Behavioural pharmacology 9: 479-498.
- Fleming TR, & Richardson BA (2004). Some design issues in trials of microbicides for the prevention of HIV infection. J Infect Dis 190: 666-674.
- Gajria K, Lu M, Sikirica V, Greven P, Zhong Y, Qin P, et al. (2014). Adherence, persistence, and medication discontinuation in patients with attention-deficit/hyperactivity disorder - a systematic literature review. Neuropsychiatric disease and treatment 10: 1543-1569.
- Gasser P (1996). Die psycholytische Therapie in der Schweiz von 1988-1993. Schweiz Arch Neurol Psychiatr 147: 59-65.
- Gasser P, Holstein D, Michel Y, Doblin R, Yazar-Klosinski B, Passie T, et al. (2014). Safety and efficacy of lysergic acid diethylamide-assisted psychotherapy for anxiety associated with life-threatening diseases. J Nerv Ment Dis 202: 513-520.
- Gasser P, Kirchner K, & Passie T (2015). LSD-assisted psychotherapy for anxiety associated with a life- threatening disease: a qualitative study of acute and sustained subjective effects. J Psychopharmacol 29: 57- 68.
- Gouzoulis-Mayfrank E, Schneider F, Friedrich J, Spitzer M, Thelen B, & Sass H (1998). Methodological issues of human experimental research with hallucinogens. Pharmacopsychiatry 31 Suppl 2: 114-118.
- Griffiths R, Richards W, Johnson M, McCann U, & Jesse R (2008). Mystical-type experiences occasioned by psilocybin mediate the attribution of personal meaning and spiritual significance 14 months later. J Psychopharmacol 22: 621-632.
- Griffiths RR, Johnson MW, Richards WA, Richards BD, McCann U, & Jesse R (2011). Psilocybin occasioned mystical-type experiences: immediate and persisting dose-related effects. Psychopharmacology 218: 649- 665.
- Griffiths RR, Richards WA, McCann U, & Jesse R (2006). Psilocybin can occasion mysticaltype experiences having substantial and sustained personal meaning and spiritual significance. Psychopharmacology 187: 268-283; discussion 284-292.
- Grob CS, Danforth AL, Chopra GS, Hagerty M, McKay CR, Halberstadt AL, et al. (2011). Pilot study of psilocybin treatment for anxiety in patients with advanced-stage cancer. Archives of general psychiatry 68: 71-78.
- Grof S, Goodman LE, Richards WA, & Kurland AA (1973). LSD-assisted psychotherapy in patients with terminal cancer. Int Pharmacopsychiatry 8: 129-144.
- Haile CN, Murrough JW, Iosifescu DV, Chang LC, Al Jurdi RK, Foulkes A, et al. (2014). Plasma brain derived neurotrophic factor (BDNF) and response to ketamine in treatmentresistant depression. Int J Neuropsychopharmacol 17: 331-336.
- Halpern JH, & Pope HG, Jr. (1999). Do hallucinogens cause residual neuropsychological toxicity? Drug and alcohol dependence 53: 247-256.
- Hintzen A, & Passie T (2010) The pharmacology of LSD: a critical review. Oxford University Press: Oxford.
- Hofmann A (1979). How LSD originated. J Psychedelic Drugs 11: 53-60.
- Holland D, & Passie T (2011) Flaschback-Phänomene. Verlag für Wissenschaft und Bildung: Berlin, Germany.
- Holze F, Duthaler U, Vizeli P, Muller F, Borgwardt S, & Liechti ME (2019). Pharmacokinetics and subjective effects of a novel oral LSD formulation in healthy patients. Br J Clin Pharmacol 85: 1474-1483.
- Holze F, Liechti ME, Hutten N, Mason NL, Dolder PC, Theunissen EL, Duthaler U, Feilding A, Ramaekers JG, & Kuypers KPC (2021a). Pharmacokinetics and pharmacodynamics of lysergic acid diethylamide microdoses in healthy participants. Clin Pharmacol Ther 109: 658666.

- Holze F, Vizeli P, Ley L, Muller F, Dolder P, Stocker M, Duthaler U, Varghese N, Eckert A, Borgwardt S, & Liechti ME (2021b). Acute dose-dependent effects of lysergic acid diethylamide in a double-blind placebo-controlled study in healthy subjects. Neuropsychopharmacology 46: 537-544.
- Holze F, Vizeli P, Muller F, Ley L, Duerig R, Varghese N, et al. (2020). Distinct acute effects of LSD, MDMA, and D-amphetamine in healthy patients. Neuropsychopharmacology 45: 462471.
- Hurks PP, & Hendriksen JG (2011). Retrospective and prospective time deficits in childhood
- ADHD: The effects of task modality, duration, and symptom dimensions. Child Neuropsychol 17: 34-50.
- Hurks PP, & van Loosbroek E (2014). Time estimation deficits in childhood mathematics difficulties. J Learn Disabil 47: 450-461.
- Hutten N, Mason NL, Dolder P, Theunissen EL, Holze F, Liechti ME, Varghese N, Eckert A, Feilding A, Ramaekers JG, & Kuypers KP (2020a). Low dose LSD acutely increases BDNF blood plasma levels in healthy volunteers. ACS Pharmacol Transl Sci 4: 461-466.
- Hutten N, Mason NL, Dolder PC, & Kuypers KPC (2019a). Motives and Side-Effects of Microdosing With Psychedelics Among Users. Int J Neuropsychopharmacol 22: 426-434.
- Hutten N, Mason NL, Dolder PC, & Kuypers KPC (2019b). Self-Rated Effectiveness of Microdosing With Psychedelics for Mental and Physical Health Problems Among Microdosers. Front Psychiatry 10: 672.
- Hutten N, Mason NL, Dolder PC, Theunissen EL, Holze F, Liechti ME, Feilding A, Ramaekers JG, & Kuypers KPC (2020b). Mood and cognition after administration of low LSD doses in healthy volunteers: A placebo controlled dose-effect finding study. Eur Neuropsychopharmacol.
- Johansen PO, & Krebs TS (2015). Psychedelics not linked to mental health problems or suicidal behavior: a population study. J Psychopharmacol 29: 270-279.
- Johnson M, Richards W, & Griffiths R (2008). Human hallucinogen research: guidelines for safety. J Psychopharmacol 22: 603-620.
- Johnson MW, Garcia-Romeu A, & Griffiths RR (2016). Long-term follow-up of psilocybinfacilitated smoking cessation. The American journal of drug and alcohol abuse 43: 55-60.
- Johnson MW, Garcia-Romeu A, Cosimano MP, & Griffiths RR (2014). Pilot study of the 5HT2AR agonist psilocybin in the treatment of tobacco addiction. J Psychopharmacol 28: 983992.
- Johnson MW, Sewell RA, & Griffiths RR (2012). Psilocybin dose-dependently causes delayed, transient headaches in healthy volunteers. Drug and alcohol dependence 123: 132-140.
- Johnston LD, O'Malley PM, Bachmann JG, Schulenberg JE, & Miech RA (2016). Monitoring the future: national survey results on drug use, 1975-2015: Volume 2. College students and adults ages 19-55 Ann Arbor: Institute for Social Research, University of Michigan.
- Kaelen M, Barrett FS, Roseman L, Lorenz R, Family N, Bolstridge M, et al. (2015). LSD enhances the emotional response to music. Psychopharmacology 232: 3607-3614.
- Kaelen M, Roseman L, Kahan J, Santos-Ribeiro A, Orban C, Lorenz R, et al. (2016). LSD modulates music- induced imagery via changes in parahippocampal connectivity. European neuropsychopharmacology: the journal of the European College of Neuropsychopharmacology 26: 1099-1109.
- Karst M, Halpern JH, Bernateck M, & Passie T (2010). The non-hallucinogen 2-bromo-lysergic acid diethylamide as preventative treatment for cluster headache: an open, non-randomized case series. Cephalalgia 30: 1140-1144.
- Kessler RC, Adler L, Ames M, Demler O, Faraone S, Hiripi E, et al. (2005). The World Health Organization Adult ADHD Self-Report Scale (ASRS): a short screening scale for use in the general population. Psychol Med 35: 245-256.
- Kessler RC, Adler L, Barkley R, Biederman J, Conners CK, Demler O, et al. (2006). The prevalence and correlates of adult ADHD in the United States: results from the National Comorbidity Survey Replication. Am J Psychiatry 163: 716-723.
- Koelle GB (1958). The pharmacology of mescaline and D-lysergic acid diethylamide (LSD). N Engl J Med 258: 25-32.

- Kraehenmann R, Pokorny D, Aicher H, Preller KH, Pokorny T, Bosch OG, et al. (2017a). LSD Increases Primary Process Thinking via Serotonin 2A Receptor Activation. Front Pharmacol 8: 814.
- Kraehenmann R, Pokorny D, Vollenweider L, Preller KH, Pokorny T, Seifritz E, et al. (2017b). Dreamlike effects of LSD on waking imagery in humans depend on serotonin 2A receptor activation. Psychopharmacology 234: 2031-2046.
- Krebs TS, & Johansen PO (2012). Lysergic acid diethylamide (LSD) for alcoholism: metaanalysis of randomized controlled trials. J Psychopharmacol 26: 994-1002.
- Krebs TS, & Johansen PO (2013a). Over 30 million psychedelic users in the United States. F1000 Res 2: 98.
- Krebs TS, & Johansen PO (2013b). Psychedelics and mental health: a population study. PLoS One 8: e63972.
- Kupferschmidt K (2014). High hopes. Science 345: 18-23.
- Kurland AA, Unger S, Shaffer JW, & Savage C (1967). Psychedelic therapy utilizing LSD in the treatment of the alcoholic patient: a preliminary report. Am J Psychiatry 123: 1202-1209.
- Kuypers KP, Ng L, Erritzoe D, Knudsen GM, Nichols CD, Nichols DE, et al. (2019). Microdosing psychedelics: More questions than answers? An overview and suggestions for future research. J Psychopharmacol 33: 1039-1057.
- Kuypers KP, Wingen M, Samyn N, Limbert N, & Ramaekers JG (2007). Acute effects of nocturnal doses of MDMA on measures of impulsivity and psychomotor performance throughout the night. Psychopharmacology 192: 111-119.
- Lea T, Amada N, & Jungaberle H (2019). Psychedelic Microdosing: A Subreddit Analysis. J Psychoactive Drugs: 1-12.
- Lea T, Amada N, Jungaberle H, Schecke H, & Klein M (2020). Microdosing psychedelics: Motivations, subjective effects and harm reduction. The International journal on drug policy 75: 102600.
- Lebedev AV, Kaelen M, Lovden M, Nilsson J, Feilding A, Nutt DJ, et al. (2016). LSD-induced entropic brain activity predicts subsequent personality change. Hum Brain Mapp 37: 32033213.
- Liechti ME (2017). Modern clinical research on LSD. Neuropsychopharmacology 42: 21142127.
- Liechti ME, Dolder PC, & Schmid Y (2017). Alterations in conciousness and mystical-type experiences after acute LSD in humans. Psychopharmacology 234: 1499-1510.
- Liechti ME, Gamma A, & Vollenweider FX (2001). Gender differences in the subjective effects of MDMA. Psychopharmacology 154: 161-168.
- Liester MB (2014). A review of lysergic acid diethylamide (LSD) in the treatment of addictions: historical perspectives and future prospects. Curr Drug Abuse Rev 7: 146-156.
- Logan GD, Schachar R, & Tannock R (1997). Impulsivity and inhibitory control. Psychol Sci 8: 60-64.
- LSD-assisted psychotherapy in persons suffering from anxiety associated with advanced-stage life threatening diseases. A phase-II, double-blind, placebo-controlled dose-response pilot study (study protocol). [Online] [Accessed: 8/2/2012 2012].
- Ludwig A, Levine J, Stark L, & Lazar R (1969). A clinical study of LSD treatment in alcoholism. Am J Psychiatry 126: 59-69.
- Luethi D, Hoener MC, Krahenbuhl S, Liechti ME, & Duthaler U (2019). Cytochrome P450 enzymes contribute to the metabolism of LSD to nor-LSD and 2-oxo-3-hydroxy-LSD: Implications for clinical LSD use. Biochem Pharmacol 164: 129-138.
- Ly C, Greb AC, Cameron LP, Wong JM, Barragan EV, Wilson PC, et al. (2018). Psychedelics Promote Structural and Functional Neural Plasticity. Cell Rep 23: 3170-3182.
- MacLean KA, Johnson MW, & Griffiths RR (2011). Mystical experiences occasioned by the hallucinogen psilocybin lead to increases in the personality domain of openness. J Psychopharmacol 25: 1453-1461.
- Mangini M (1998). Treatment of alcoholism using psychedelic drugs: a review of the program of research. J Psychoactive Drugs 30: 381-418.
- Matheson L, Asherson P, Wong IC, Hodgkins P, Setyawan J, Sasane R, et al. (2013). Adult ADHD patient experiences of impairment, service provision and clinical management in England: a qualitative study. BMC Health Serv Res 13: 184.

- Mitchell SH (1999). Measures of impulsivity in cigarette smokers and non-smokers. Psychopharmacology 146: 455-464.
- Moreno FA, Wiegand CB, Taitano EK, & Delgado PL (2006). Safety, tolerability, and efficacy of psilocybin in 9 patients with obsessive-compulsive disorder. The Journal of clinical psychiatry 67: 1735-1740.
- Moriyama TS, Polanczyk GV, Terzi FS, Faria KM, & Rohde LA (2013). Psychopharmacology and psychotherapy for the treatment of adults with ADHD-a systematic review of available meta-analyses. CNS Spectr 18: 296-306.
- Mueller F, Lenz C, Dolder PC, Harder S, Schmid Y, Lang UE, et al. (2017a). Acute effects of LSD on amygdala activity during processing of fearful stimuli in healthy patients. Transl Psychiatry 7: e1084.
- Mueller F, Lenz C, Dolder PC, Lang UE, Schmidt A, Liechti ME, et al. (2017b). Increased thalamic resting-state connectivity as a core driver of LSD-induced hallucinations. Acta Psychiatr Scand 136: 648-657.
- Muller F, Dolder PC, Schmidt A, Liechti ME, & Borgwardt S (2018). Altered network hub connectivity after acute LSD administration. Neuroimage Clin 18: 694-701.
- Nichols DE (2004). Hallucinogens. Pharmacol Ther 101: 131-181.
- Nichols DE (2016). Psychedelics. Pharmacological reviews 68: 264-355.
- Nichols DE, Johnson MW, & Nichols CD (2017). Psychedelics as Medicines: An Emerging New Paradigm. Clinical pharmacology and therapeutics 101: 209-219.
- Oosterlaan J, Logan GD, & Sergeant JA (1998). Response inhibition in AD/HD, CD, comorbid AD/HD + CD, anxious, and control children: a meta-analysis of studies with the stop task. Journal of child psychology and psychiatry, and allied disciplines 39: 411-425.
- Pahnke WN, Kurland AA, Goodman LE, & Richards WA (1969). LSD-assisted psychotherapy with terminal cancer patients. Curr Psychiatr Ther 9: 144-152.
- Pahnke WN, Kurland AA, Unger S, Savage C, & Grof S (1970). The experimental use of psychedelic (LSD) psychotherapy. Jama 212: 1856-1863.
- Passie T (2019) The science of microdosing psychedelics. Psychedelic Press, London, UK.
- Passie T, & Halpern JH (2014). The pharmacology of hallucinogens. In The ASAM principles of addiction medicine. ed Ries R., K. Wolters Kluver: Alphen aan de Rijn, The Netherlands, pp 235-255.
- Passie T, Halpern JH, Stichtenoth DO, Emrich HM, & Hintzen A (2008). The pharmacology of lysergic acid diethylamide: a review. CNS Neurosci Ther 14: 295-314.
- Polito V, & Stevenson RJ (2019). A systematic study of microdosing psychedelics. PLoS One 14: e0211023.
- Posner, K., K Brown, G., Stanley, B., A Brent, D., Yershova, K., Oquendo, M., … Mann, J. (2011). The Columbia-Suicide Severity Rating Scale: Initial Validity and Internal Consistency Findings From Three Multisite Studies With Adolescents and Adults (Vol. 168).
- Preller KH, Burt JB, Ji JL, Schleifer CH, Adkinson BD, Stampfli P, et al. (2018). Changes in global and thalamic brain connectivity in LSD-induced altered states of consciousness are attributable to the 5-HT2A receptor. Elife 7.
- Preller KH, Herdener M, Pokorny T, Planzer A, Kraehenmann R, Stämpfli P, et al. (2017). The fabric of meaning and subjective effects in LSD-induced states depend on serotonin 2A receptor activation Curr Biol 27: 451-457.
- Preller KH, Razi A, Zeidman P, Stampfli P, Friston KJ, & Vollenweider FX (2019). Effective connectivity changes in LSD-induced altered states of consciousness in humans. Proc Natl Acad Sci U S A 116: 2743-2748.
- Prochazkova L, Lippelt DP, Colzato LS, Kuchar M, Sjoerds Z, & Hommel B (2018). Exploring the effect of microdosing psychedelics on creativity in an open-label natural setting. Psychopharmacology 235: 3401- 3413.
- Ramaekers JG, Hutten N, Mason NL, Dolder P, Theunissen EL, Holze F, Liechti ME, Feilding A, & Kuypers KP (2020). A low dose of lysergic acid diethylamide decreases pain perception in healthy volunteers. J Psychopharmacol: 269881120940937.
- Rickli A, Luethi D, Reinisch J, Buchy D, Hoener MC, & Liechti ME (2015). Receptor interaction profiles of novel N-2-methoxybenzyl (NBOMe) derivatives of 2,5-dimethoxysubstituted phenethylamines (2C drugs). Neuropharmacology 99: 546-553.
- Rickli A, Moning OD, Hoener MC, & Liechti ME (2016). Receptor interaction profiles of novel psychoactive tryptamines compared with classic hallucinogens. European

neuropsychopharmacology: the journal of the European College of Neuropsychopharmacology 26: 1327-1337.

- Roseman L, Sereno MI, Leech R, Kaelen M, Orban C, McGonigle J, et al. (2016). LSD alters eyes-closed functional connectivity within the early visual cortex in a retinotopic fashion. Hum Brain Mapp 37: 3031- 3040.
- Ross S (2012). Serotonergic hallucinogens and emerging targets for addiction pharmacotherapies. Psychiatr Clin North Am 35: 357-374.
- Rubia K, Oosterlaan J, Sergeant JA, Brandeis D, & v Leeuwen T (1998). Inhibitory Dysfunction in hyperactive boys. Behav Brain Res 94: 25-32.
- Savage C, & McCabe OL (1973). Residential psychedelic (LSD) therapy for narcotic addict: a controlled study. Archives of general psychiatry 28: 808-814.
- Schindler EA, Gottschalk CH, Weil MJ, Shapiro RE, Wright DA, & Sewell RA (2015). Indoleamine hallucinogens in cluster headache: results of the clusterbusters medication use survey. J Psychoactive Drugs 47: 372-381.
- Schmid Y, & Liechti ME (2018). Long-lasting subjective effects of LSD in normal patients. Psychopharmacology 235: 535-545.
- Schmid Y, Enzler F, Gasser P, Grouzmann E, Preller KH, Vollenweider FX, et al. (2015). Acute effects of lysergic acid diethylamide in healthy patients. Biol Psychiatry 78: 544-553.
- Schmid Y, Gasser P, Oehen P, & Liechti ME (2021). Acute subjective effects in LSD- and MDMA-assisted psychotherapy. J Psychopharmacol 35: 362-374.
- Schmidt A, Mueller F, Lenz C, Dolder PC, Schmid Y, Zanchi D, et al. (2017). Acute LSD effects on response inhibition neuronal networks. Psychol Med 48: 1464-1473.
- Sewell RA, Halpern JH, & Pope HG, Jr. (2006). Response of cluster headache to psilocybin and LSD. Neurology 66: 1920-1922.
- Speth J, Speth C, Kaelen M, Schloerscheidt AM, Feilding A, Nutt DJ, et al. (2016). Decreased mental time travel to the past correlates with default-mode network disintegration under lysergic acid diethylamide. J Psychopharmacol 30: 344-353.
- Steuer AE, Poetzsch M, Stock L, Eisenbeiss L, Schmid Y, Liechti ME, et al. (2017). Development and validation of an ultra-fast and sensitive microflow liquid chromatographytandem mass spectrometry (MFLC-MS/MS) method for quantification of LSD and its metabolites in plasma and application to a controlled LSD administration study in humans. Drug Test Anal 9: 788-797.
- Strajhar P, Schmid Y, Liakoni E, Dolder PC, Rentsch KM, Kratschmar DV, et al. (2016). Acute effects of lysergic acid diethylamide on circulating steroid levels in healthy patients. J Neuroendocrinol 28: 12374.
- Strassman RJ (1984). Adverse reactions to psychodelic drugs: a review of the literature J Nerv Ment Dis 172:577-595.
- Studerus E, Gamma A, & Vollenweider FX (2010). Psychometric evaluation of the altered states of consciousness rating scale (OAV). PLoS One 5: e12412.
- Studerus E, Gamma A, Kometer M, & Vollenweider FX (2012). Prediction of psilocybin response in healthy volunteers. PLoS One 7: e30800.
- Studerus E, Kometer M, Hasler F, & Vollenweider FX (2011). Acute, subacute and long-term subjective effects of psilocybin in healthy humans: a pooled analysis of experimental studies. J Psychopharmacol 25: 1434-1452.
- Tagliazucchi E, Roseman L, Kaelen M, Orban C, Muthukumaraswamy SD, Murphy K, et al. (2016). Increased global functional connectivity correlates with LSD-induced ego dissolution. Curr Biol 26: 1043-1050.
- Terhune DB, Luke DP, Kaelen M, Bolstridge M, Feilding A, Nutt D, et al. (2016). A placebocontrolled investigation of synaesthesia-like experiences under LSD. Neuropsychologia 88: 2834.
- Vollenweider FX, Vollenweider-Scherpenhuyzen MF, Babler A, Vogel H, & Hell D (1998). Psilocybin induces schizophrenia-like psychosis in humans via a serotonin-2 agonist action. Neuroreport 9: 3897-3902.
- Wigal SB (2009). Efficacy and safety limitations of attention-deficit hyperactivity disorder pharmacotherapy in children and adults. CNS drugs 23 Suppl 1: 21-31.
- Wilens TE, & Dodson W (2004). A clinical perspective of attention-deficit/hyperactivity disorder into adulthood. The Journal of clinical psychiatry 65: 1301-1313.

- Yanakieva S, Polychroni N, Family N, Williams LTJ, Luke DP, & Terhune DB (2019). The effects of microdose LSD on time perception: a randomised, double-blind, placebo-controlled trial. Psychpharmacology 236:1159-1167.

18. APPENDICES

18.1. APPENDIX 1 LIST OF PROHIBITED MEDICATIONS, SUPPLEMENTS, AND OTHER THERAPIES

Drug Class or Drug Name	Washout Conditions/ Prohibition Period [a]	Allowable Conditions for use during study
Benzodiazepines	Last dose must have been taken at least 5 half-lives prior to Day -1 /baseline	To treat anxiety during study at Investigator's discretion [b]
Lithium	Last dose must have been taken at least 7 days prior to Day -1 /baseline	None
Monoamine oxidase inhibitors (MAOIs)[c]	Last dose must have been taken at least 3 weeks prior to Day-1 /baseline	None
Antipsychotics – traditional<br>Antipsychotics – atypical<br>Atypical agents (e.g., bupropion, mirtazapine)<br>Barbiturates<br>Selective serotonin reuptake inhibitors (SSRIs). For fluoxetine and its active metabolite, norfluoxetine, half-life (T1/2) of metabolite is more extended than other SSRIs and T1/2 of 7 to 15 days should be allowed.<br>Serotonin-norepinephrine reuptake inhibitors (SNRIs)<br>Serotonin–norepinephrine–dopamine reuptake inhibitors (SNDRIs)<br>Serotonin modulators (e.g., vortioxetine, trazodone)<br>Tricyclic antidepressants (TCAs) Stimulants to treat AHD (e.g., methylphenidate hydrochloride, amphetamines)<br>Non-stimulants to treat ADHD	Last dose must have been taken at least 5 half-lives prior to Day -<br>1/baseline	None
Other medications, e.g., efavirenz, supplements, herbal treatments, or therapeutics that affect<br>serotonergic function (e.g., ginkgo biloba, St. John's Wort, 5hydroxytryptophan [5- HTP], ayahuasca, dimethyltryptamine [DMT], and opioids, in particular, tramadol, ketamine, dextromethorphan, meperidine, methadone, and other agents that inhibit the reuptake of	Last dose must have been taken at least 5 half-lives prior to Day 1/Baseline	None

serotonin)		

a. Subjects on prohibited medications, supplements, or other therapeutics at the time of Screening may taper off that substance(s) prior to Day -1/Baseline (Visit 2) and will be eligible to participate in the study if the required washout conditions have been met.
b. Benzodiazepines should only be offered and administered if non-pharmacological interventions (e.g., reassurance, verbal communication) do not adequately address severe anxiety symptoms.
c. Although the half-life of MAOIs is typically 1.5-4 hours (eliminated quickly), the irreversible inhibition of monoamine oxidase may persist for 2-3 weeks due to the requirement for de novo synthesis of new enzyme, which is a relatively slow process. Therefore, washout for any MAOI should be conservatively 3 weeks.

18.2. APPENDIX 2 DEFINITION OF WOMEN OF CHILDBEARING POTENTIAL (WOCBP) AND ACCEPTABLE HIGHLY EFFECTIVE METHODS OF CONTRACEPTION

A woman of childbearing potential (WOCBP) is defined as a woman (or nonbinary/transgender individual) who is physiologically capable of becoming pregnant.
Women are considered to be not of childbearing potential if they meet at least one of the following conditions:

- Has undergone total hysterectomy, bilateral salpingectomy, bilateral oophorectomy, or bilateral tubal ligation at least 2 months prior to Screening
- Has a congenitally absent uterus
- Is postmenopausal, confirmed as being age > 45 years with one of the following:
    - At least 1 year without menses prior to Screening
    - 6 months to < 1 year without menses prior to Screening and serum FSH levels > 40 mIU/mL at Screening.

Acceptable Highly Effective Methods of Contraception

Highly effective birth control methods are methods that can achieve a failure rate of less than 1% per year when used consistently and correctly. The following highly effective methods of contraception are acceptable for use by WOCBP:

- Oral, injected or implanted hormonal methods of contraception o Combined estrogen and progestogen hormonal contraception (oral, intravaginal, transdermal) associated with inhibition of ovulation; these should be initiated at least 3 months prior to Screening o Progestogen-only hormonal contraception (oral, injectable, implantable) associated with inhibition of ovulation; these should be initiated at least 3 months prior to Screening
- Established intrauterine device (IUD) or intrauterine hormone-releasing system (IUS) (in place for at least 2 months prior to Screening)
- Surgical sterilization (hysterectomy, bilateral salpingectomy, bilateral oophorectomy, or bilateral tubal ligation at least 2 months prior to Screening)
- Sexual activity is limited to a male partner who has undergone effective surgical sterilization provided that partner is the sole sexual partner of the female study subject
- Heterosexual abstinence is only considered a highly effective method if abstinence is defined as refraining from heterosexual intercourse for a period that covers the entire period of potential drug and drug effect exposures. As such, the effectiveness of sexual abstinence needs to be evaluated in relation to the duration of the clinical study, the duration of drug effects, and the preferred and usual lifestyle of the participant. In the case of this study, minimum time adherence prior to first dosing is 3 months, and must continue for 3 months after dosing.

Unacceptable Methods of Contraception

Periodic abstinence (calendar, “symptothermal”, and post-ovulation methods), withdrawal (coitus interruptus), spermicides only and lactational amenorrhea method are not acceptable methods of contraception. A female condom and male condom should not be used together.

18.3. APPENDIX 3 LIST OF CYTOCHROME P450 2D6 (CYP2D6) INHIBITORS

Strong inhibitors	Moderate inhibitors
• Bupropion<br>• Dacomitinib<br>• Fluoxetine<br>• Paroxetine<br>• Quinidine<br>• Tipranavir<br>•	• Abiraterone<br>• Cinacalcet<br>• Darifenacin<br>• Darunavir<br>• Duloxetine<br>• Givosiran<br>• Lorcaserin<br>• Mirabegron<br>• Perhexiline*<br>• Rolapitant<br>• Terbinafine (systemic)<br>• Thioridazine

- This table lists strong and moderate CYP450 2D6 inhibitors; there are no known clinically relevant inducers of CYP2D6.
- Inhibitors of CYP2D6 metabolism listed above can alter serum concentrations of other drugs that are dependent on CYP2D6 liver enzymes for activation or elimination:
  - Codeine, tamoxifen, and tramadol are examples of drugs that require transformation by CYP2D6 to their active metabolite(s). The presence of CYP2D6 inhibitors can **decrease** efficacy of these drugs.
  - Amitriptyline, clozapine, desipramine, flecainide, haloperidol, nortriptyline, risperidone, and valbenazine are examples of drugs that are eliminated by CYP2D6 metabolism. The presence of CYP2D6 inhibitors can **increase** levels of these drugs.
- The specific effect of CYP2D6 inhibition on CYP2D6 substrate blood levels varies widely among individual patients because of variability in CYP2D6 function (ie, genetic polymorphism). Poor, intermediate, extensive, and ultrarapid CYP2D6 function types have been well characterized.
- These classifications are based upon US Food and Drug Administration (FDA) guidance.[1,2] Other sources may use a different classification system resulting in some agents being classified differently.
- For additional information on CYP2D6 drug metabolism, refer to the UpToDate topic review of pharmacogenomics, section on CYP2D6 variants, and clinical topic reviews of the use of these agents and their drug interactions.
- Specific drug interactions and management suggestions may be determined by using the Lexicomp drug interactions program included with UpToDate. Refer to UpToDate topics on specific agents and indications for further details.

CYP2D6 References:

1. US Food and Drug Administration. Clinical drug interaction studies — Cytochrome P450 enzyme- and transporter-mediated drug interactions guidance for industry, January 2020. Available at: https://www.fda.gov/regulatory-information/search-fda-guidancedocuments/clinical-drug-interaction-studies-cytochrome-p450-enzyme-andtransporter-mediated-drug-interactions (Accessed on June 5, 2020).
2. US Food and Drug Administration. Drug Development and Drug Interactions: Table of Substrates, Inhibitors and Inducers. Available at: FDA.gov website.
