# Supplementary material for: Safety and Efficacy of Repeated Low-Dose LSD for ADHD Treatment in Adults: A Randomized Clinical Trial
Source: JAMA Psychiatry. 2025 Mar 19;82(6):555–62. doi: 10.1001/jamapsychiatry.2025.0044 (PMC11923771; doi:10.1001/jamapsychiatry.2025.0044)
Supplement: Supplement 2. — eTable 1. Study Schedule eMethods. Supplementary Methods eTable 2. List of Prohibited Medications eAppendix. Statistical Analysis eTable 3. Dropouts eResults. Supplementary Results eTable 4. Guessed and Actual Allocation eTable 5. MMRM of the Adult ADHD Investigator Symptom Rating Scale AISRS eTable 6. MMRM of the ADHD Self-Report Scale ASRS eTable 7. MMRM of the Conners' Adult ADHD Rating Scale A (Inattention/Memory Problems) eTable 8. MMRM of the Conners' Adult ADHD Rating Scale B (Hyperactivity/Restlessness) eTable 9. MMRM of the Conners' Adult ADHD Rating Scale C (Impulsivity/Emotional Lability) eTable 10. MMRM of the Conners' Adult ADHD Rating Scale D (Problems with Self-Concept) eTable 11. MMRM of the CAARS E (DSM-IV Inattentive Symptoms) eTable 12. MMRM of the Conners' Adult ADHD Rating Scale F (DSM-IV Hyperactive-Impulsive Symptoms) eTable 13. MMRM of the Conners' Adult ADHD Rating Scale G (DSM-IV ADHD Symptoms Total) eTable 14. MMRM of the Conners' Adult ADHD Rating Scale H (ADHD Index) eTable 15. Descriptive Statistics of Efficacy Outcomes (Change From Baseline) eTable 16. Descriptive Statistics of Efficacy Outcomes (Absolute Values) eFigure 1. AISRS, ASRS, MMRM eFigure 2. Clinical Global Impression-Severity eFigure 3. Conners' Adult ADHD Rating Scale MMRM eFigure 4. Outcomes After 6 Weeks Stratified by Guessed Allocation MMRM eTable 17. Acute Effects Measured by Visual Analog Scales eFigure 5. Acute Effects Measured by Visual Analog Scales eFigure 6. Acute Effects Measured by Visual Analog Scales (Spaghetti Plots) eTable 18. Acute Effects on the 5 Dimensions of Altered States of Consciousness Scale and Mystical Experiences Questionnaire eFigure 7. Acute Effects on the 5 Dimensions of Altered States of Consciousness Scale and Mystical Experiences Questionnaire eTable 19. Pharmacokinetic Parameters of LSD (20 µg) Determined by Non-Compartmental Analysis eFigure 8. Pharmacokinetics of LSD and O-H-LSD eTable 20. Adverse Events eTable 21. Related Adverse Event [file jamapsychiatry-e250044-s002.pdf]

Supplemental Online Content

Mueller L, Santos de Jesus J, Schmid Y, et al. Safety and efficacy of repeated low-dose LSD for ADHD treatment in adults: a randomized, double-blind, placebo-controlled randomized clinical trial. *JAMA Psychiatry*. Published online March 19, 2025. doi:10.1001/jamapsychiatry.2025.0044

eTable 1. Study Schedule
eMethods. Supplementary Methods
eTable 2. List of Prohibited Medications
eAppendix. Statistical Analysis
eTable 3. Dropouts
eResults. Supplementary Results
eTable 4. Guessed and Actual Allocation
eTable 5. MMRM of the Adult ADHD Investigator Symptom Rating Scale AISRS
eTable 6. MMRM of the ADHD Self-Report Scale ASRS
eTable 7. MMRM of the Conners' Adult ADHD Rating Scale A (Inattention/Memory Problems)
eTable 8. MMRM of the Conners' Adult ADHD Rating Scale B (Hyperactivity/Restlessness)
eTable 9. MMRM of the Conners' Adult ADHD Rating Scale C (Impulsivity/Emotional Lability)
eTable 10. MMRM of the Conners' Adult ADHD Rating Scale D (Problems with Self-Concept)
eTable 11. MMRM of the CAARS E (*DSM-IV* Inattentive Symptoms)
eTable 12. MMRM of the Conners' Adult ADHD Rating Scale F (*DSM-IV* Hyperactive-Impulsive Symptoms)
eTable 13. MMRM of the Conners' Adult ADHD Rating Scale G (*DSM-IV* ADHD Symptoms Total)
eTable 14. MMRM of the Conners' Adult ADHD Rating Scale H (ADHD Index)
eTable 15. Descriptive Statistics of Efficacy Outcomes (Change From Baseline)
eTable 16. Descriptive Statistics of Efficacy Outcomes (Absolute Values)
eFigure 1. AISRS, ASRS, MMRM
eFigure 2. Clinical Global Impression-Severity
eFigure 3. Conners' Adult ADHD Rating Scale MMRM
eFigure 4. Outcomes After 6 Weeks Stratified by Guessed Allocation MMRM
eTable 17. Acute Effects Measured by Visual Analog Scales
eFigure 5. Acute Effects Measured by Visual Analog Scales
eFigure 6. Acute Effects Measured by Visual Analog Scales (Spaghetti Plots)
eTable 18. Acute Effects on the 5 Dimensions of Altered States of Consciousness Scale and Mystical Experiences Questionnaire
eFigure 7. Acute Effects on the 5 Dimensions of Altered States of Consciousness Scale and Mystical Experiences Questionnaire
eTable 19. Pharmacokinetic Parameters of LSD (20 µg) Determined by Non-Compartmental Analysis
eFigure 8. Pharmacokinetics of LSD and O-H-LSD
eTable 20. Adverse Events
eTable 21. Related Adverse Events
eTable 22. Suicidal Ideations
eTable 23. Electrocardiographic Parameters
eFigure 9. Blood Pressure and Heart Rate During the First Dose Administration
eTable 24. Laboratory Values at Different Visits
eReferences.

This supplemental material has been provided by the authors to give readers additional information about their work.

eTable 1. Study Schedule

	**Screening**	**Baseline[a]**	**Dosing begins**	**6-week Blinded Treatment Period (20 µg LSD vs. Placebo)**			**End of study/early termination**
Time	Up to 4 weeks prior to Day -1	Day -1	Day 1	Twice weekly Dosing* (start = Day 1)	Every 2 weeks (first dose of the week)**	Week 6 (before final dose)***	Week 10 follow-up****
Medical/surgical history	X						
Inclusion/exclusion criteria	X	X					
Pregnancy test	X		X	X			X
Physical examination	X					X	X
Weight and BMI	X					X	X
Height	X					X	X
Vital signs	X	X	X	X		X	X
Clinical Laboratory Tests	X				X	X	X
Pharmacokinetics			X[1]				
Urine Drug test	X	X	X		X[2]	X[2]	
Pharmacodynamics (5D-ASC & MEQ30)			X			X[3]	
12-lead ECG[b]	X		X[4]			X[4]	
Cognitive Performance Test		X				X	
CAARS[c]	X	X			X	X	X
ASRS		X			X	X	X
AISRS[d], CGI-S[d]	X	X			X	X	X
MINI	X						
C-SSRS	X						
C-SSRS since last visit (SLV)		X	X	X		X	X
VAS			X[5]				
Check daily diary	X	X	X	X		X	X
Randomization			X				
Drug administration			X	X			
Adverse events	X	X	X	X		X	X
Concomitant medication	X	X	X	X		X	X

5D-ASC, 5 dimensions of altered states of consciousness scale; AISRS, Adult ADHD Investigator Symptom Rating Scale; ASRS = Adult ADHD Self-Report Scale; BMI = body mass index; BP = blood pressure; CAARS = Conners' Adult ADHD Rating Scale; CGI-S = Clinical Global Impression Scale; C-SSRS = Columbia-Suicide Severity Rating Scale; ECG = electrocardiogram; MEQ30 = Mystical Experience Questionnaire (30-item); VAS = Visual Analog Scale.

*	Twice weekly dosing occurred every 3 to 4 days (e.g., every Monday and Wednesday) with a ± 1 day window.
**	Outcomes of weeks 2 and 4 were assessed at the beginning of the respective dosing week. Week 2 was at day 8 or after 2 doses; week 4 at day 22 or after 6 doses. AISRS and CGI-S week 6 was assessed before the last dose at day 40.
***	CAARS and ASRS were assessed twice during week 6 (at day 36 and 40).
****	For persons withdrawing from the study the final visit was coordinated to occur up to 4 weeks after the last administered dose.

a	Baseline visit could be done up to 3 days prior to Day 1 e.g., if it was done on a Friday and Day 1 on a Monday.
b	Resting in supine position for 5 minutes prior to ECG collection.
c	CAARS-O-RS conducted at screening. All other assessments and shown results used CAARS-L-SR.
d	AISRS and CGI-S were assessed at weeks 2 and 6 during the dosing period but not week 4
X^1	PK samples collected pre-dose, 0.5, 1, 2, 3, 4, and 6 hours post-dose.
X^2	2 random drug tests were administered during the study.
X^3	Pharmacodynamic scales completed at home up to 6 hours after dosing.
X^4	2 hours after dosing.
X^5	Collected pre-dose, 0.5, 1, 2, 3, 4, and 6 hours post-dose.

eMethods. Supplementary Methods

Study approval

The trial was approved by the following authorities.
Basel:

- Ethikkommission Nord-West und Zentral-Schweiz (EKNZ), Basel, Switzerland ID: BASEC2020-01296
- Swissmedic, Bern, Switzerland
- Federal Office of Public Health, Bern, Switzerland

Maastricht:

- Medical Research Ethics Review Committee (MREC), Academic Hospital Maastricht and University of Maastricht (METC 20-037)
- Central Committee on Research Involving Human Subjects (NL73910.068.20)

Inclusion criteria

All inclusion criteria were based on the judgment of the Investigator. For inclusion in the study, subjects had to meet all the following criteria:

1. Ability and willingness to provide written, informed consent prior to initiation of any study-related procedures and to adhere to all study requirements.
2. Age ≥ 18 and ≤ 65 years at Screening.
3. Presence of the diagnosis of Diagnostic and Statistical Manual of Mental Disorders-4/5 (DSM-4/5) ADHD, as determined by previous and documented psychiatric evaluation (written report was required) and confirmed by structured interview (MINI).
4. AISRS total score of ≥ 26 at screening.
5. CGI-S score of ≥ 4 at screening.
6. Must be willing to receive IMP dose twice weekly. On day 1, the subject will come to the site clinic and must be willing to take a taxi or public transportation home or be accompanied by a caregiver and not drive a car, use heavy equipment, or participate in any other dangerous activity for the remainder of the day after receiving IMP.
7. Must be willing to refrain from consuming more than 6 standard alcoholic drinks per week (1 standard drink corresponds to 0.1 L wine, 0.3 L beer, or 40 mL liquor), more than 10 cigarettes a day, and more than 2 cups of coffee a day throughout the study treatment period (6 weeks) and until the last study visit is complete.

Exclusion criteria

1. Past or present diagnosis of a primary psychotic disorder or first-degree relative with a psychotic disorder.
2. Past or present bipolar disorder (DSM-4/5).
3. Other current psychiatric disorders that, in the opinion of the Investigator or medical supervisor, may confound the results of the study (e.g., obsessive-compulsive disorder, dysthymic disorder, panic disorder, dissociative disorder, anorexia nervosa or bulimia nervosa).
4. Subjects with past (> 1 month prior to the screening visit) or present substance use disorder (except nicotine, provided subject does not smoke more than 10 cigarettes a day).
5. Somatic disorders including Central Nervous System (CNS) involvement of cancer, severe cardiovascular disease, untreated hypertension, severe liver disease (liver enzyme increase by more than 3x the upper limit of normal except unconjugated hyperbilirubinemia due to Gilbert's Disease, per Investigator), severely impaired renal function (estimated creatinine clearance < 50 mL/min by CKD-EPI formula), or anything else that, in the judgment of the Investigator or medical supervisor, poses too great a potential for side effects.
6. Any lifetime history of suicide attempt; or recent (within 6 months prior to the screening visit) active suicidal thoughts or ideation (defined as a suicidal ideation score of 2 or greater in the Columbia-Suicide Severity Rating Scale [C-SSRS]); or endorsement of any suicidal behavior on the C-SSRS within the past 6 months prior to the screening visit.
7. Likely to require psychiatric hospitalization during the study.
8. Once consent is signed, subject not willing or able to stop any prescription or nonprescription ADHD medications during screening and prior to the baseline visit through final study visit.

9. Plan to start, stop, or alter the use of any medications, supplements, or other therapeutics from Baseline until the final visit.
10. Plan to start, stop, or alter the use of psychotherapy, massage, meditation, acupuncture, hypnosis, yoga, or other similar therapy/activity from the time of providing informed consent until the final visit.
11. Use of potent CYP2D6 inhibitors; moderate CYP2D6 inhibitors by Investigator discretion.
12. Likely to need use of any psychiatric medications with the potential to confound interpretation of study results or impact safety, at the discretion of the Investigator, in the 10 weeks following Baseline up to the final visit.
13. Use of investigational medication/treatment in the past 30 days prior to the screening visit.
14. Subjects with a positive urine drug screen (except for THC or metabolites) at Screening or Baseline.
15. Clinically significant abnormal baseline laboratory values, and ECG that include the following:
    a. Evidence of clinically significant hepatic disorder (e.g., alanine aminotransferase [ALT] or aspartate aminotransferase [AST] > 3X ULN (except for Gilbert's disease), and
    b. Any clinically significant abnormal metabolic or hematologic screen, per Investigator or medical supervisor decision.
    c. Exclusionary blood pressure: >140 mm Hg (systolic) or >90 mm Hg (diastolic); heart rate <45 beats/minute or >90 beats/minute after an approximately 5-minute supine or semi-supine rest
    d. Exclusionary ECG parameters: QTcF > 450 msec (men), QTcF >470 msec (women)
    e. Any clinically significant abnormal electrocardiogram (ECG) finding (e.g., uncontrolled atrial fibrillation, ischemia) at Screening or Baseline, as determined by the Investigator or medical supervisor (in consultation with a cardiologist, if needed.
16. Any other condition, therapy, laboratory abnormality, or other circumstance that, in the opinion of the Investigator or medical supervisor, may pose additional risk to the subject from participation in the study, may interfere with the subject's ability to comply with study procedures, may make participation in the study not in the subject's best interest or may confound the results of the study.
17. Prior history or ongoing neuropsychiatric signs or symptoms associated with COVID19 such as development of, or current disorder, during or after a COVID-19 infection including anxiety, memory loss, confusion, depression, delirium, agitation, or psychosis.
18. Women of childbearing potential (WOCBP) (i.e., physiologically capable of becoming pregnant) who are unwilling or unable to use a highly effective method of contraception, for the duration of the study, or Men physiologically capable of fathering a child who are sexually active with WOCBP but are unwilling or unable to use barrier contraception (e.g., condom with or without spermicidal cream or jelly) for the duration of the study.
19. Women who are currently pregnant or breastfeeding or plan to become pregnant or breastfeed during the study.
20. Men who plan to donate sperm during the study.
21. Use of weight loss drugs within 21 days of screening until the end of study.
22. Subjects who are either unable or unwilling to consume alcohol in any amount (including due to religious or personal reasons).
23. Subjects who have a change in AISRS score of ≥13-points between screening and baseline visits.

Randomization and blinding

Eligible participants were randomly assigned in a 1:1 ratio to receive either LSD or a placebo. Randomization was stratified by site, using computer-generated randomization with balanced blocks of sizes 2, 4, and 6. The sponsor conducted the randomization and provided the allocation details to the medication producer. The randomization list remained sealed until the completion of the study and closure of the database, ensuring that all study staff and participants remained blinded to the treatment allocation until the study's conclusion. In case of an emergency requiring immediate unblinding, the PI and the GMP facility had access to sealed envelopes containing the allocation details. One such case occurred during the study, following a positive pregnancy test and the exclusion of one participant.

Composition of the investigational medicinal product

Active: Vials contained LSD-tartrate 0.029 mg (corresponding to 0.020 mg LSD base) and ethanol 0.16 g in aqua pur ad 1 mL

Placebo: Vials contained ethanol 0.16 g aqua pur ad 1 mL

GMP-grade LSD tartrate was produced by Onyxd Scientific Inc, Sunderland, UK. The investigational medicinal products were formulated according to Good Manufacturing Practice (GMP) by a Swissmedic-licensed GMP manufacturer (Apotheke Dr. Hysek, Biel, Switzerland).

Treatment

Administration of the IMP was twice a week, every 3 to 4 days for 6 weeks. The administration occurred at the study site under supervision of study personnel. After the first dose administration participants remained at the study site for 6 hours for assessment of acute effects, pharmacokinetics, vitals, and tolerability. On further dosing days participants were released after dose administration.

Concomitant medication

Any prior and newly started concomitant medication was recorded. The use of medications, supplements, and other therapeutics (over the counter or prescribed) for treating ADHD, anxiety, depression, or mood disorders was prohibited from baseline until the end of study visit. All participants taking such medication prior to enrollment had to taper them with a sufficient wash-out period of at least 5 plasma elimination half-lives of the respective medication prior to baseline.

Psychometric scales for ADHD

Adult ADHD Investigator Symptom Rating Scale (AISRS)

The AISRS[1], is a clinician-administered scale that assesses each of the DSM-5 symptoms of ADHD. The AISRS not only uses the adult ADHD prompts from the Adult ADHD Clinical Diagnostic Scale (ACDS) v1.2, but each of the stem questions are designed to better capture symptoms of the disorder as they present in adulthood. It has been used and validated many times in a variety of clinical drug trials. The AISRS total score consists of 18 items from the original ADHD-RS, which were derived based on DSM-5 criteria for ADHD. The ADHD-RS includes 9 items that address symptoms of inattention, and 9 items that address symptoms of impulsivity and hyperactivity. Each item is rated from 0 to 3. The AISRS total score can range from 0 to 54. A higher score corresponds to a worse severity of ADHD.

Clinical Global Impression-Severity (CGI-S)

The CGI-S rating scale[2] measures symptom severity, treatment response, and the efficacy of treatments in treatment studies of a variety of psychiatric and somatic disorders. It consists of a 7-point scale that requires the trained investigator to rate the severity of the subject's illness at the time of assessment. Possible ratings are: 1: normal, not at all ill; 2: borderline mentally ill; 3: mildly ill; 4: moderately ill; 5: markedly ill; 6: severely ill; 7: among the most extremely ill subjects. We calculated within-subject difference to baseline values, as well as the number of persons achieving an improvement of ≥ 1 points.

Adult Attention-Deficit/Hyperactivity Disorder Self-Reporting Rating Scale (ASRS)

The ASRS[3] was developed in conjunction with the World Health Organization (WHO), and the Workgroup on Adult ADHD. It is a self-report questionnaire designed to assess ADHD symptoms in adults based on DSM-5 criteria and consists of 18 items, each rated on a 5-point scale from 0 to 4. The total score can range from 0 to 72, with higher scores reflecting more severe symptoms.

Connors' Adult ADHD Rating Scale (CAARS)

The CAARS Self-Report Long Form (CAARS-L-SR)[4] was used which is a 66-item measure of ADHD symptoms that was designed as a self-report assessment for adult ADHD. Responses are scored on a 4-point scale, where 0 = not at all, 1 = just a little, 2 = pretty much, and 3 = very much and show symptoms over the following 8 subscales: A=Inattention/Memory Problems, B=Hyperactivity/Restlessness, C=Impulsivity/Emotional Lability, D=Problems with Self-Concept, E=DSM-IV Inattentive Symptoms, F=DSM-IV Hyperactive-Impulsive Symptoms, G=DSM-IV ADHD Symptoms Total, H=ADHD Index. Test – retest reliabilities have been shown to be strong. The CAARS is highly correlated with other self-report ADHD measures, and initial studies on diagnostic

accuracy for adult ADHD found the CAARS to have a good diagnostic sensitivity and specificity relative to healthy controls. We used within-subject difference to baseline of raw scores for each subscale for the analysis.

Daily Diary

To explore sleep quality and duration, the subjects were given a home diary for recording subjective sleep duration and quality (data not shown in this publication). The outcome of this measure is not included in the present analysis.

The Columbia-Suicide Severity Rating Scale (C-SSRS)

The C-SSRS[5] is a validated instrument to assess a subject's suicidal ideation or behavior over a specified time interval. Suicidal ideation is assessed on a scale from 1 (wish to be dead) to 5 (active suicidal ideation with specific plan and intent). Suicidal behavior is assessed based on responses to individual prompts about prior suicidal actions.

Acute subjective effects

Acute subjective effects of LSD and placebo were assessed for 6 hours after administration on Day 1 using Visual Analog Scales (VASs), the 5 Dimensions of Altered States of Consciousness Questionnaire (5D-ASC), and Mystical Experience Questionnaire 30 item (MEQ30). The 5D-ASC and MEQ30 were administered after acute effects had subside and characterize psychedelic alterations of the mind and mystical-type experiences, respectively. In the present study the questionnaires were self-administered on site on the first dosing day and at home after the last dosing.

Visual Analog Scales (VAS)

A series of single item VAS were used at time points 0, 0.5, 1, 2, 3, 4 and 6 hours after the first drug administration. The following VAS items were used: "any drug effect", "good drug effect", bad drug effect", "drug liking", "fear", nausea", "alteration of vision", "alteration of sense of time", and "the boundaries between myself and my surroundings seem to blur"[6-8]. Effect strength is marked on a 100 mm line and then measured. Maximal effects (E_{max}) were obtained directly from the observed data. Area under the effect-time curve (AUEC) was calculated using linear trapezoidal methods in Phoenix WinNonlin 8.4 (Certara, Princeton, NJ, USA).

5 Dimensions of Altered States of Consciousness scale (5D-ASC)

The 5D-ASC scale[9,10] is a visual analog scale consisting of 94 items. The instrument is constructed of five scales, and allows assessing mood, anxiety, derealization, depersonalization, changes in perception, auditory alterations, and reduced vigilance. The scale is well-validated and has been used to characterize the acute subjective effects of LSD in experimental studies. The 5D-ASC scale was administered 6 h after dosing of the first and last dose, and subjects were instructed to retrospectively rate peak alterations. Each item of the scale is scored on a 0-100 mm VAS. Results are shown as percentages of maximal possible effects per dimension or subscale.

Mystical Experiences Questionnaire (MEQ30)

This 30-item questionnaire[11] is rated on a six-point scale. The scale has been used to assess mystical experiences in studies using psilocybin and LSD. The MEQ30 was administered 6 hours after drug administration of the first and last dose, and participants are instructed to retrospectively rate peak alterations. Reported data on each domain scale is expressed as a percentage of the maximum possible score.

Pharmacokinetics

Repeated blood sampling was done after the first dose administration at time points 0, 0.5, 1, 2, 3, 4 and 6 hours. The actual date and exact time of collection of each sample was recorded. Blood samples were collected in heparinized/EDTA tubes, centrifuged at 4°C at 3000 rpm for 10 min, and the plasma was then stored at -20°C and -80°C until analysis. Plasma levels of LSD and of the main LSD-metabolite 2-oxo-3-hydroxy-LSD (O-H-LSD) were analyzed using a fully validated ultra-high-performance liquid chromatography tandem mass spectrometry method as previously described[12]. An improved extraction method[13] was used. In contrast to other data, the pharmacokinetic data was not obtained from the sponsor but generated in the analytics unit of the Basel study site. The lower limit of quantification (LLOQ) was 1 pg/mL for LSD and 2.5 pg/mL O-H-LSD respectively.

The data for the pharmacokinetic analysis was obtained directly from the analytics unit of the Basel site. Pharmacokinetic parameters were calculated using non-compartmental analysis (NCA) in Phoenix WinNonlin 8.4 (Certara, Princeton, NJ, USA). Peak plasma concentration (C_{max}) and time to C_{max} (T_{max}) were obtained directly from the observed data. The terminal elimination rate constant (λ_z) was estimated by log-linear regression after semilogarithmic transformation of the data using at least three data points of the linear phase of the concentration-time curve. The area under the concentration–time curve (AUC) from 0 to 6 h postdosing (AUC_6) was computed employing the linear-up log-down method. The infinite AUC (AUC_∞) was derived by extrapolating the AUC_6 using the constant λ_z. The pharmacokinetic parameters were determined for each participant using the actual precise sampling times.

Laboratory measurements

Routine blood plasma laboratory analysis, urine drug screening and pregnancy tests were done according to the study schedule (eTable 1). The following parameters were measured:

Standard Chemistry:

- Albumin
- Total protein
- Alkaline phosphatase
- ALT
- AST
- Bilirubin, total (+ direct if total is >ULN)
- Blood urea nitrogen or urea
- Calcium
- Sodium
- Chloride
- Creatinine
- Creatinine clearance (CKD-EPI)
- FSH (to confirm postmenopausal status)
- GGT
- Glucose
- Lactate dehydrogenase
- Phosphate
- Potassium

Hematology complete blood count, including:

- Hemoglobin
- Hematocrit
- MCV, MCH
- Platelet count
- Red blood cell count
- White blood cell count

Differential count if WBC is elevated, including:

- Basophils, Eosinophils, Lymphocytes, Monocytes and Neutrophils

Urine pregnancy test

Urine drug screening

ALT: Alanine Aminotransferase; AST: Aspartate Aminotransferase; CKD-EPI: Chronic Kidney Disease Epidemiology Collaboration equation; FSH: Follicle-Stimulating Hormone; GGT: Gamma-Glutamyl Transferase; MCH: Mean Corpuscular Hemoglobin; MCV: Mean Corpuscular Volume; RBC: Red Blood Cell count; WBC: White blood cell count.

Resting Electrocardiogram (ECG)

A 12-lead ECG was obtained after the subject had been resting in the supine position for at least 5 minutes. All abnormal waveforms were noted, heart rate, all intervals including PR, RR, QRS, QT, and QTcF intervals from the 12-lead ECG (Fridericia's formula was used for calculation of heart-rate corrected QT-time). The Investigator judged the overall interpretation as normal or abnormal. If abnormal, it was then decided whether the abnormality was clinically significant or not clinically significant.

Adverse Events

Adverse events (AEs) were documented at each visit. Any occurrence of an AE, Adverse Reaction (AR), Serious Adverse Event (SAE), Serious Adverse Reaction (SAR), and Suspected Unexpected Serious Adverse Reaction (SUSAR) was recorded and coded using the Medical Dictionary for Regulatory Activities (MedDRA 25.1). Total counts, as well as unique AEs per subject (repeated occurrences of the same AE within one subject only counted once) were calculated. Further evaluation

included assessment of the relationship to the study medication, severity, and clinical course in accordance with Good Clinical Practice (GCP) guidelines.

Causality was described as follows:

**Relationship**	**Description**
Unrelated	There is no evidence of any causal relationship.
Unlikely	There is little evidence to suggest there is a causal relationship (e.g., the event did not occur within a reasonable time after administration of the IMP). There is another reasonable explanation for the event (e.g., the participant's clinical condition, other concomitant treatment).
Possible	There is some evidence to suggest a causal relationship (e.g., because the event occurs within a reasonable time after administration of the IMP). However, the influence of other factors may have contributed to the event (e.g. the participant's clinical condition, other concomitant treatments).
Probable	There is evidence to suggest a causal relationship and the influence of other factors is unlikely.
Definitely	There is clear evidence to suggest a causal relationship and other possible contributing factors can be ruled out.

AEs are considered related to the treatment if relationship is classified as definitely, probable, or possible.

Investigators

Assessment of all outcome measures was done by blinded, trained study personnel (study nurses and psychologists), not by independent raters.
Physical examination, as well as interpretation of ECG and laboratory values, medical history, concomitant medication, and adverse events, was done by blinded physicians.
The ADHD diagnosis was established by a psychiatrist in clinical practice outside the study prior to inclusion. A written report of the diagnosis was acquired for all participants and reviewed by a study physician.

eTable 2. List of Prohibited Medications

Drug Class or Drug Name	Washout Conditions / Prohibition Period	Allowable Conditions for use during study
Benzodiazepines	Last dose must have been taken at least 5 half-lives prior to baseline	To treat anxiety during study at Investigator's discretion
Lithium	Last dose must have been taken at least 7 days prior to baseline	None
Monoamine oxidase inhibitors (MAOIs)	Last dose must have been taken at least 3 weeks prior to baseline	None
Antipsychotics – traditional or atypical<br>Barbiturates<br>Selective serotonin reuptake inhibitors (SSRIs): For fluoxetine and its active metabolite, norfluoxetine, half-life ($T_{1/2}$) of active metabolite is more extended than other SSRIs and $T_{1/2}$ of 7 to 15 days should be allowed.<br>Serotonin-norepinephrine reuptake inhibitors (SNRIs)<br>Serotonin–norepinephrine– dopamine reuptake inhibitors (SNDRIs)<br>Serotonin modulators (e.g., vortioxetine, trazodone)<br>Tricyclic antidepressants (TCAs)<br>Stimulants to treat ADHD<br>Non-stimulants to treat ADHD	Last dose must have been taken at least 5 half-lives prior to baseline	None
Other medications, e.g., efavirenz, supplements, herbal treatments, or therapeutics that affect serotonergic function (e.g., ginkgo biloba, St. John's Wort, 5-hydroxytryptophan [5-HTP], ayahuasca, dimethyltryptamine [DMT], and opioids, in particular, tramadol, ketamine, dextromethorphan, meperidine, methadone, and other agents that inhibit the reuptake of serotonin)	Last dose must have been taken at least 5 half-lives prior to baseline	None

eAppendix. Statistical Analysis

See also the statistical analysis part of the study protocol (Supplement 1).

Primary outcome

The predetermined primary outcome was the least square mean (LSM) change in ADHD symptoms from baseline to week 6, assessed using the AISRS. This was evaluated using a Mixed Model for Repeated Measures (MMRM). Changes in AISRS scores at week 2 and week 6 were incorporated as dependent variables. Fixed effects included treatment group, visit, visit-by-treatment group interaction, and covariates of baseline AISRS score, sex, and age. An unstructured variance-covariance matrix modeled within-subject errors with Satterthwaite's method to approximate degrees of freedom. The analysis included all randomized persons who received at least one dose per the intention-to-treat principle. LSM changes from baseline with 95% confidence intervals (CIs) were calculated for each group, and between-group LSM differences were analyzed with a one-sided α-level of 0.1.

Handling of missing/potentially biased values:

Missing or potentially biased data were handled using multiple imputation (MI). Depending on the Intercurrent Event (IE) causing the missing or potentially biased value, two different strategies were applied:

Strategy 1; missing not at random (MNAR), in cases of:

- intake or change in concomitant medications/therapies which have potential confounding effects.
- intake of prohibited medications/therapies.
- dropouts possibly related to the study.

Strategy 2; missing at random (MAR), applied for:

- IEs related to COVID-19 or other intercurrent missing data.
- dropouts considered unrelated to the study.

Dropouts were assessed on a case-by-case basis for classification as MAR or MNAR (see eTable 3 for details).

The imputation model included the longitudinal sequence of AISRS scores, and the covariates specified in the primary outcome. For missing AISRS data, 1000 datasets were generated using a random seed of 200208.

The multiple imputation process followed these steps:

1. Imputation assuming MAR (strategy 2):
    a) All data collected after the IEs were kept in the database.
    b) Missing data were imputed based on MAR assumptions, borrowing information from participants in the same treatment group using the fully conditional specification (FCS) method. These datasets were used in Step 2.
2. Imputation assuming MNAR (strategy 1):
    a) data recorded after a potentially confounding IE (defined under strategy 1) were set to missing.
    b) Missing values were imputed using MNAR assumptions, borrowing information from the placebo arm subjects via reference-based imputation.
    c) The MMRM described above was run on each of the 1000 imputed datasets, and Rubin's rule was applied to combine results for statistical inference.

Sensitivity analyses

As a sensitivity analysis, the MMRM for the primary outcome was repeated using only MAR assumptions and without imputation. Additionally, subgroups based on ADHD severity were analyzed, with severe ADHD defined as a baseline AISRS score of ≥38, and moderate ADHD as a baseline score of <38.

Secondary outcomes

The statistical analysis plan did not foresee any formal statistical analyses beyond descriptive statistics for secondary efficacy outcomes. However, post hoc analyses were conducted on the AISRS, ASRS,

and CAARS, using the difference to baseline values of raw scores for all respective outcome parameters.
The same MMRM and imputation methods applied to the primary outcome were used for these analyses, with the following modifications: All available time points were included in the model and a two-sided α-level of 0.05 was used for between-group comparisons.
Since these analyses were post hoc, correction for multiple testing was only considered in the event of significant findings.
A further adjustment was done to assess the influence of guessed allocation. Here instead of the actual treatment allocation, we used the participants guessed allocation after 6 weeks of treatment.

Additionally, we investigated associations between various potential predictors, including acute drug effects (VAS, 5D-ASC, MEQ-30), baseline characteristics, and outcomes using the available data without imputation.

eResults. Supplementary Results

Blinding

Guessed treatment allocation was assessed after the first and after the last dosing day. Blinding data are missing for 3 participants who completed the study at the Maastricht site and for 4/7 dropouts. For 3 dropouts, data are available with 'Guess last dose' being assessed after 5 doses (all 3 dropped out after 5 doses).

Pharmacokinetic parameters

Pharmacokinetic parameters were determined and are shown for LSD. The mean R-squared value for determination of the terminal half-life was 0.95 and > 0.8 in all 27 profiles. The mean fraction extrapolated of the AUC from the observation period (6h) to infinity was 35%.
The main metabolite O-H-LSD was measured but still rising plasma concentrations at the last measured time point of 6 hours made a valid calculation of pharmacokinetic parameters impossible.

eTable 3. Dropouts

Allocation	Reason for dropping out	Administered doses	Handling of missing data
Placebo	positive pregnancy test	5	MAR
Placebo	consent withdrawal (recurring depression, needs to restart therapy with bupropion)	5	MNAR
Placebo	consent withdrawal (felt no benefit)	6	MNAR
LSD	consent withdrawal (acute effects too strong)	1	MNAR
LSD	consent withdrawal (daughter had accident, travel to study site takes too much time)	3	MAR
LSD	consent withdrawal (felt no benefit)	4	MNAR
LSD	consent withdrawal (acute effects too strong)	5	MNAR

All 7 dropouts with reasons, number of administered doses and handling of missing data in the mixed effect model. MAR: missing at random; MNAR: missing not at random.

eTable 4. Guessed and Actual Allocation

**After first dose**			
	Had LSD	Had Placebo	Total (%)
Guessed LSD	23	17	40/50 (80%)
Guessed Placebo	2	8	10/50 (20%)
Total	25	25	50/50 (100%)
Guessed correctly	23/25 (92%)	8/25 (32%)	31/50 (62%)

**After last dose** including 3/7 dropouts			
	Had LSD	Had Placebo	Total (%)
Guessed LSD	21	16	37/46 (80%)
Guessed Placebo	1	8	9/46 (20%)
Total	22	24	46/46 (100%)
Guessed correctly	21/22 (95%)	8/24 (33%)	29/46 (63%)

**After last dose** without dropouts			
	Had LSD	Had Placebo	Total (%)
Guessed LSD	20	15	35/43 (81%)
Guessed Placebo	1	7	8/43 (19%)
Total	21	22	43/43 (100%)
Guessed correctly	20/21 (95%)	7/22 (32%)	27/43 (63%)

eTable 5. MMRM of the Adult ADHD Investigator Symptom Rating Scale AISRS

		LSD (*n*=27)		Placebo (*n*=26)		Difference	
Model	Week	LSM	(95% CI)	LSM	(95% CI)	LSM	(95% CI)
MNAR	2	-3.4	(-4.7 to -2.0)	-5.1	(-6.5 to -3.7)	1.7	(-0.23 to 3.7)
	6	-7.1	(-10 to -4.0)	-8.9	(-12 to -5.8)	1.8	(-2.5 to 6.2)
	10 Follow-up	-7.1	(-10 to -4.0)	-6.7	(-9.8 to -3.5)	-0.38	(-4.8 to 4.0)
MAR	2	-3.3	(-4.6 to -2.0)	-5.1	(-6.5 to -3.7)	1.8	(-0.2 to 3.7)
	6	-7.1	(-10 to -4.0)	-8.9	(-12 to -5.8)	1.8	(-2.5 to 6.2)
	10 Follow-up	-7.3	(-10 to -4.3)	-6.7	(-9.8 to -3.6)	-0.64	(-5.0 to 3.7)
Missing, adjusted	2	-3.3	(-4.7 to -2.0)	-5.1	(-6.5 to -3.7)	1.8	(-0.26 to 3.8)
	6	-7.1	(-10 to -4.0)	-8.9	(-12 to -5.7)	1.8	(-2.7 to 6.3)
	10 Follow-up	-7.3	(-10 to -4.2)	-6.7	(-9.9 to -3.5)	-0.65	(-5.1 to 3.8)
Missing, not adjusted	2	-3.3	(-4.7 to -2.0)	-4.8	(-6.1 to -3.4)	1.5	(-0.45 to 3.4)
	6	-7.1	(-10 to -4.1)	-8.5	(-12 to -5.5)	1.4	(-2.9 to 5.7)
	10 Follow-up	-7.3	(-10 to -4.3)	-6.3	(-9.4 to -3.2)	-1.0	(-5.4 to 3.4)

Secondary efficacy outcomes determined by Linear Mixed effects Models (MMRM) on the intention to treat data. LSM, least square mean change from baseline; CI, Confidence interval; MNAR, missing not at random; MAR, missing at random. Model specifications were as follows: MNAR = adjusted for covariates, missing data imputed not at random and at random (see method section of primary paper); MAR = adjusted for covariates, all missing data imputed as MAR; Missing adjusted = no imputations of missing data, adjusted for covariates; Missing not adjusted = no imputations of missing data and no adjustment for covariates. Follow-up was during week 10 for persons completing the study and 2-4 weeks after the last dose for dropouts.

eTable 6. MMRM of the ADHD Self-Report Scale ASRS

		LSD (*n*=27)		Placebo (*n*=26)		Difference	
Model	Week	LSM	(95% CI)	LSM	(95% CI)	LSM	(95% CI)
MNAR	2	-6.9	(-9.9 to -4.0)	-7.0	(-10 to -3.9)	0.019	(-4.3 to 4.3)
	4	-8.5	(-12 to -5.0)	-9.5	(-13 to -5.9)	1.0	(-4.0 to 6.0)
	6/1	-11	(-15 to -6.8)	-11	(-16 to -7.3)	0.60	(-5.1 to 6.3)
	6/2	-13	(-17 to -8.5)	-13	(-17 to -8.3)	-0.043	(-6.0 to 5.9)
	10 Follow-up	-12	(-16 to -7.1)	-9.8	(-15 to -5.0)	-1.9	(-8.7 to 4.8)
MAR	2	-6.9	(-9.9 to -4.0)	-7.0	(-10 to -3.9)	0.027	(-4.3 to 4.3)
	4	-8.4	(-12 to -4.9)	-9.5	(-13 to -5.9)	1.1	(-4.0 to 6.2)
	6/1	-11	(-15 to -6.7)	-11	(-16 to -7.4)	0.74	(-5.0 to 6.5)
	6/2	-13	(-17 to -8.4)	-13	(-17 to -8.4)	0.004	(-6.0 to 6.0)
	10 Follow-up	-12	(-17 to -7.4)	-9.8	(-15 to -5.0)	-2.2	(-9.0 to 4.5)
Missing, adjusted	2	-7.1	(-10 to -4.1)	-7.1	(-10 to -4.0)	0.035	(-4.4 to 4.4)
	4	-8.5	(-12 to -4.9)	-9.6	(-13 to -6.0)	1.1	(-4.1 to 6.3)
	6/1	-11	(-15 to -6.7)	-12	(-16 to -7.4)	0.76	(-5.2 to 6.7)
	6/2	-13	(-17 to -8.4)	-13	(-17 to -8.4)	0.033	(-6.1 to 6.2)
	10 Follow-up	-12	(-17 to -7.4)	-10	(-15 to -5.0)	-2.2	(-9.2 to 4.7)
Missing, not adjusted	2	-8.1	(-11 to -4.7)	-6.7	(-10 to -3.3)	-1.4	(-6.2 to 3.3)
	4	-9.5	(-13 to -5.7)	-9.2	(-13 to -5.5)	-0.26	(-5.5 to 5.0)
	6/1	-12	(-16 to -7.2)	-11	(-16 to -6.7)	-0.48	(-6.8 to 5.9)
	6/2	-14	(-18 to -9.1)	-12	(-17 to -7.7)	-1.4	(-7.9 to 5.1)
	10 Follow-up	-13	(-18 to -7.7)	-9.7	(-15 to -4.4)	-3.3	(-11 to 4.2)

Secondary efficacy outcomes determined by Linear Mixed effects Models (MMRM) on the intention to treat data. LSM, least square mean change from baseline; CI, Confidence interval; MNAR, missing not at random; MAR, missing at random. Model specifications were as follows: MNAR = adjusted for covariates, missing data imputed not at random and at random (see method section of primary paper); MAR = adjusted for covariates, all missing data imputed as MAR; Missing adjusted = no imputations of missing data, adjusted for covariates; Missing not adjusted = no imputations of missing data and no adjustment for covariates. Time point 6/1 equals treatment day 36; 6/2 equals treatment day 40; Follow-up was at week 10 for subjects completing the study and 2-4 weeks after the last dose for dropouts.

eTable 7. MMRM of the Conners' Adult ADHD Rating Scale A (Inattention/Memory Problems)

		**LSD (*n*=27)**		**Placebo (*n*=26)**		**Difference**	
Model	Week	LSM	(95% CI)	LSM	(95% CI)	LSM	(95% CI)
MNAR	2	-3.0	(-4.8 to -1.3)	-3.7	(-5.6 to -1.9)	0.68	(-1.9 to 3.3)
	4	-5.1	(-7 to -3.3)	-6.4	(-8.3 to -4.5)	1.2	(-1.4 to 3.9)
	6/1	-5.4	(-7.5 to -3.3)	-7.6	(-9.8 to -5.4)	2.2	(-0.81 to 5.2)
	6/2	-5.9	(-8.1 to -3.6)	-6.3	(-8.6 to -4.0)	0.42	(-2.8 to 3.6)
	10 Follow-up	-5.1	(-7.6 to -2.6)	-5.7	(-8.4 to -3.1)	0.65	(-3.0 to 4.3)
MAR	2	-3.0	(-4.8 to -1.3)	-3.6	(-5.5 to -1.8)	0.60	(-2.0 to 3.2)
	4	-5.1	(-6.9 to -3.2)	-6.3	(-8.2 to -4.4)	1.2	(-1.5 to 4.0)
	6/1	-5.1	(-7.2 to -3.0)	-7.5	(-9.7 to -5.4)	2.5	(-0.56 to 5.5)
	6/2	-5.9	(-8.1 to -3.7)	-6.2	(-8.5 to -3.9)	0.33	(-2.9 to 3.6)
	10 Follow-up	-5.1	(-7.6 to -2.6)	-5.7	(-8.3 to -3.1)	0.60	(-3.0 to 4.2)
Missing, adjusted	2	-3.1	(-4.9 to -1.2)	-3.7	(-5.6 to -1.8)	0.62	(-2.0 to 3.3)
	4	-5.1	(-7.0 to -3.2)	-6.3	(-8.3 to -4.4)	1.3	(-1.5 to 4.0)
	6/1	-5.1	(-7.3 to -2.9)	-7.6	(-9.8 to -5.4)	2.5	(-0.63 to 5.6)
	6/2	-5.9	(-8.2 to -3.6)	-6.3	(-8.6 to -3.9)	0.35	(-3.0 to 3.6)
	10 Follow-up	-5.1	(-7.7 to -2.5)	-5.7	(-8.4 to -3.1)	0.61	(-3.1 to 4.3)
Missing, not adjusted	2	-3.6	(-5.5 to -1.6)	-3.1	(-5.1 to -1.2)	-0.46	(-3.2 to 2.3)
	4	-5.6	(-7.5 to -3.7)	-5.8	(-7.7 to -3.8)	0.18	(-2.6 to 2.9)
	6/1	-5.5	(-8.0 to -2.9)	-6.9	(-9.4 to -4.4)	1.4	(-2.2 to 5.0)
	6/2	-6.4	(-8.8 to -3.9)	-5.7	(-8.2 to -3.2)	-0.69	(-4.2 to 2.8)
	10 Follow-up	-5.5	(-8.3 to -2.6)	-5.1	(-8.0 to -2.1)	-0.40	(-4.5 to 3.7)

Secondary efficacy outcomes determined by Linear Mixed effects Models (MMRM) on the intention to treat data. LSM, least square mean change from baseline; CI, Confidence interval; MNAR, missing not at random; MAR, missing at random. Model specifications were as follows: MNAR = adjusted for covariates, missing data imputed not at random and at random (see method section of primary paper); MAR = adjusted for covariates, all missing data imputed as MAR; Missing adjusted = no imputations of missing data, adjusted for covariates; Missing not adjusted = no imputations of missing data and no adjustment for covariates. Time point 6/1 equals treatment day 36; 6/2 equals treatment day 40; Follow-up was at week 10 for subjects completing the study and 2-4 weeks after the last dose for dropouts.

eTable 8. MMRM of the Conners' Adult ADHD Rating Scale B (Hyperactivity/Restlessness)

		**LSD (*n*=27)**		**Placebo (*n*=26)**		**Difference**	
Model	Week	LSM	(95% CI)	LSM	(95% CI)	LSM	(95% CI)
MNAR	2	-1.9	(-3.9 to 0.13)	-4.8	(-6.9 to -2.7)	2.9	(-0.12 to 5.9)
	4	-3.3	(-5.7 to -0.93)	-5.9	(-8.3 to -3.4)	2.5	(-1.0 to 6.1)
	6/1	-4.5	(-6.6 to -2.3)	-7.1	(-9.3 to -4.9)	2.6	(-0.53 to 5.8)
	6/2	-5.0	(-7.4 to -2.6)	-5.8	(-8.3 to -3.3)	0.82	(-2.7 to 4.3)
	10 Follow-up	-4.3	(-6.5 to -2.1)	-6.2	(-8.5 to -3.8)	1.9	(-1.4 to 5.1)
MAR	2	-1.8	(-3.8 to 0.20)	-4.7	(-6.8 to -2.6)	2.9	(-0.095 to 5.9)
	4	-3.3	(-5.7 to -0.89)	-5.8	(-8.3 to -3.3)	2.5	(-0.99 to 6.1)
	6/1	-4.3	(-6.4 to -2.1)	-7.0	(-9.2 to -4.8)	2.8	(-0.40 to 5.9)
	6/2	-5.1	(-7.4 to -2.7)	-5.8	(-8.2 to -3.3)	0.68	(-2.8 to 4.2)
	10 Follow-up	-4.2	(-6.4 to -2.0)	-6.1	(-8.4 to -3.8)	1.9	(-1.4 to 5.1)
Missing, adjusted	2	-1.8	(-3.9 to 0.23)	-4.7	(-6.9 to -2.6)	2.9	(-0.16 to 6.0)
	4	-3.3	(-5.8 to -0.84)	-5.8	(-8.4 to -3.3)	2.5	(-1.1 to 6.1)
	6/1	-4.3	(-6.5 to -2.1)	-7.0	(-9.3 to -4.8)	2.8	(-0.48 to 6.0)
	6/2	-5.1	(-7.5 to -2.7)	-5.8	(-8.3 to -3.3)	0.68	(-2.9 to 4.2)
	10 Follow-up	-4.2	(-6.5 to -2.0)	-6.1	(-8.5 to -3.8)	1.9	(-1.4 to 5.2)
Missing, not adjusted	2	-3.1	(-5.4 to -0.87)	-3.3	(-5.6 to -1.1)	0.19	(-3.0 to 3.4)
	4	-4.6	(-7.1 to -2.1)	-4.4	(-6.9 to -1.9)	-0.18	(-3.8 to 3.4)
	6/1	-5.4	(-7.8 to -3.0)	-5.7	(-8.1 to -3.2)	0.24	(-3.2 to 3.7)
	6/2	-6.2	(-8.9 to -3.6)	-4.4	(-7.1 to -1.7)	-1.8	(-5.6 to 1.9)
	10 Follow-up	-5.3	(-7.9 to -2.7)	-4.7	(-7.3 to -2.2)	-0.56	(-4.2 to 3.1)

Secondary efficacy outcomes determined by Linear Mixed effects Models (MMRM) on the intention to treat data. LSM, least square mean change from baseline; CI, Confidence interval; MNAR, missing not at random; MAR, missing at random. Model specifications were as follows: MNAR = adjusted for covariates, missing data imputed not at random and at random (see method section of primary paper); MAR = adjusted for covariates, all missing data imputed as MAR; Missing adjusted = no imputations of missing data, adjusted for covariates; Missing not adjusted = no imputations of missing data and no adjustment for covariates. Time point 6/1 equals treatment day 36; 6/2 equals treatment day 40; Follow-up was at week 10 for subjects completing the study and 2-4 weeks after the last dose for dropouts.

eTable 9. MMRM of the Conners' Adult ADHD Rating Scale C (Impulsivity/Emotional Lability)

		**LSD (*n*=27)**		**Placebo (*n*=26)**		**Difference**	
Model	Week	LSM	(95% CI)	LSM	(95% CI)	LSM	(95% CI)
MNAR	2	-4.2	(-6.5 to -2.0)	-2.3	(-4.6 to 0.035)	-1.9	(-5.2 to 1.3)
	4	-5.8	(-7.8 to -3.8)	-4.8	(-6.9 to -2.8)	-0.95	(-3.8 to 1.9)
	6/1	-5.8	(-8.3 to -3.2)	-5.3	(-8.0 to -2.7)	-0.41	(-4.1 to 3.3)
	6/2	-6.2	(-8.3 to -4.0)	-5.8	(-8.0 to -3.5)	-0.4	(-3.5 to 2.7)
	10 Follow-up	-4.7	(-7.3 to -2.1)	-4.0	(-6.7 to -1.4)	-0.65	(-4.4 to 3.1)
MAR	2	-4.3	(-6.5 to -2.0)	-2.3	(-4.6 to 0.008)	-2.0	(-5.3 to 1.3)
	4	-5.8	(-7.8 to -3.8)	-4.9	(-6.9 to -2.8)	-0.93	(-3.8 to 2.0)
	6/1	-5.8	(-8.3 to -3.2)	-5.4	(-8.0 to -2.7)	-0.39	(-4.1 to 3.3)
	6/2	-6.1	(-8.3 to -4.0)	-5.8	(-8.0 to -3.6)	-0.34	(-3.5 to 2.8)
	10 Follow-up	-4.7	(-7.3 to -2.2)	-4.1	(-6.8 to -1.4)	-0.65	(-4.4 to 3.1)
Missing, adjusted	2	-4.4	(-6.7 to -2.1)	-2.4	(-4.8 to -0.035)	-2.0	(-5.3 to 1.4)
	4	-5.9	(-7.9 to -3.9)	-5.0	(-7.0 to -2.9)	-0.95	(-3.9 to 2.0)
	6/1	-5.9	(-8.5 to -3.2)	-5.4	(-8.1 to -2.8)	-0.41	(-4.2 to 3.4)
	6/2	-6.2	(-8.4 to -4.0)	-5.9	(-8.2 to -3.6)	-0.35	(-3.6 to 2.9)
	10 Follow-up	-4.8	(-7.5 to -2.2)	-4.2	(-6.9 to -1.4)	-0.67	(-4.5 to 3.2)
Missing, not adjusted	2	-5.3	(-8.0 to -2.7)	-1.8	(-4.5 to 0.80)	-3.5	(-7.2 to 0.28)
	4	-6.6	(-9.1 to -4.1)	-4.6	(-7.1 to -2.1)	-2.1	(-5.6 to 1.5)
	6/1	-6.5	(-9.7 to -3.3)	-5.2	(-8.4 to -2.0)	-1.3	(-5.8 to 3.2)
	6/2	-6.9	(-9.7 to -4.1)	-5.6	(-8.4 to -2.7)	-1.4	(-5.4 to 2.7)
	10 Follow-up	-5.5	(-8.3 to -2.7)	-3.9	(-6.7 to -1.0)	-1.7	(-5.7 to 2.4)

Secondary efficacy outcomes determined by Linear Mixed effects Models (MMRM) on the intention to treat data. LSM, least square mean change from baseline; CI, Confidence interval; MNAR, missing not at random; MAR, missing at random. Model specifications were as follows: MNAR = adjusted for covariates, missing data imputed not at random and at random (see method section of primary paper); MAR = adjusted for covariates, all missing data imputed as MAR; Missing adjusted = no imputations of missing data, adjusted for covariates; Missing not adjusted = no imputations of missing data and no adjustment for covariates. Time point 6/1 equals treatment day 36; 6/2 equals treatment day 40; Follow-up was at week 10 for subjects completing the study and 2-4 weeks after the last dose for dropouts.

eTable 10. MMRM of the Conners' Adult ADHD Rating Scale D (Problems With Self-Concept)

		**LSD (*n*=27)**		**Placebo (*n*=26)**		**Difference**	
Model	Week	LSM	(95% CI)	LSM	(95% CI)	LSM	(95% CI)
MNAR	2	-2.5	(-3.8 to -1.2)	-1.9	(-3.3 to -0.62)	-0.55	(-2.4 to 1.3)
	4	-3.4	(-4.8 to -1.9)	-2.0	(-3.5 to -0.53)	-1.3	(-3.4 to 0.75)
	6/1	-3.0	(-4.6 to -1.4)	-2.8	(-4.4 to -1.1)	-0.26	(-2.6 to 2.1)
	6/2	-3.2	(-4.7 to -1.7)	-2.4	(-3.9 to -0.79)	-0.85	(-3.0 to 1.4)
	10 Follow-up	-2.5	(-4.2 to -0.87)	-2.2	(-3.9 to -0.47)	-0.33	(-2.7 to 2.1)
MAR	2	-2.5	(-3.8 to -1.2)	-2.0	(-3.3 to -0.64)	-0.56	(-2.4 to 1.3)
	4	-3.5	(-4.9 to -2.0)	-2.0	(-3.5 to -0.56)	-1.4	(-3.5 to 0.65)
	6/1	-3.0	(-4.6 to -1.4)	-2.8	(-4.4 to -1.1)	-0.23	(-2.5 to 2.1)
	6/2	-3.3	(-4.8 to -1.8)	-2.4	(-3.9 to -0.82)	-0.91	(-3.1 to 1.3)
	10 Follow-up	-2.6	(-4.2 to -0.92)	-2.2	(-3.9 to -0.51)	-0.34	(-2.7 to 2.1)
Missing, adjusted	2	-2.5	(-3.8 to -1.2)	-1.9	(-3.3 to -0.57)	-0.55	(-2.5 to 1.4)
	4	-3.4	(-4.9 to -1.9)	-2.0	(-3.5 to -0.48)	-1.4	(-3.6 to 0.71)
	6/1	-3.0	(-4.6 to -1.3)	-2.7	(-4.4 to -1.0)	-0.22	(-2.6 to 2.2)
	6/2	-3.2	(-4.8 to -1.7)	-2.3	(-3.9 to -0.74)	-0.90	(-3.2 to 1.4)
	10 Follow-up	-2.5	(-4.2 to -0.83)	-2.2	(-3.9 to -0.43)	-0.34	(-2.8 to 2.1)
Missing, not adjusted	2	-2.8	(-4.1 to -1.4)	-1.4	(-2.8 to -0.055)	-1.3	(-3.3 to 0.59)
	4	-3.7	(-5.2 to -2.2)	-1.5	(-3.0 to -0.037)	-2.2	(-4.3 to -0.067)
	6/1	-3.2	(-4.8 to -1.6)	-2.3	(-3.9 to -0.66)	-0.55	(-2.4 to 1.3)
	6/2	-3.5	(-5.1 to -1.9)	-1.9	(-3.5 to -0.31)	-1.3	(-3.4 to 0.75)
	10 Follow-up	-2.8	(-4.5 to -1.1)	-1.7	(-3.5 to 0.013)	-0.26	(-2.6 to 2.1)

Secondary efficacy outcomes determined by Linear Mixed effects Models (MMRM) on the intention to treat data. LSM, least square mean change from baseline; CI, Confidence interval; MNAR, missing not at random; MAR, missing at random. Model specifications were as follows: MNAR = adjusted for covariates, missing data imputed not at random and at random (see method section of primary paper); MAR = adjusted for covariates, all missing data imputed as MAR; Missing adjusted = no imputations of missing data, adjusted for covariates; Missing not adjusted = no imputations of missing data and no adjustment for covariates. Time point 6/1 equals treatment day 36; 6/2 equals treatment day 40; Follow-up was at week 10 for subjects completing the study and 2-4 weeks after the last dose for dropouts.

eTable 11. MMRM of the CAARS E (DSM-IV Inattentive Symptoms)

		**LSD (*n*=27)**		**Placebo (*n*=26)**		**Difference**	
Model	Week	LSM	(95% CI)	LSM	(95% CI)	LSM	(95% CI)
MNAR	2	-2.7	(-4.2 to -1.3)	-1.8	(-3.3 to -0.31)	-0.95	(-3.0 to 1.1)
	4	-3.5	(-5.2 to -1.9)	-3.3	(-5.1 to -1.6)	-0.21	(-2.6 to 2.2)
	6/1	-4.0	(-6.0 to -2.0)	-4.5	(-6.5 to -2.5)	0.50	(-2.3 to 3.3)
	6/2	-4.1	(-6.3 to -2.0)	-4.4	(-6.6 to -2.2)	0.25	(-2.8 to 3.3)
	10 Follow-up	-3.4	(-5.8 to -0.99)	-2.7	(-5.2 to -0.23)	-0.69	(-4.2 to 2.8)
MAR	2	-2.8	(-4.2 to -1.3)	-1.8	(-3.3 to -0.33)	-0.96	(-3.0 to 1.1)
	4	-3.5	(-5.2 to -1.8)	-3.4	(-5.1 to -1.6)	-0.14	(-2.6 to 2.3)
	6/1	-3.9	(-5.9 to -1.9)	-4.5	(-6.6 to -2.5)	0.63	(-2.2 to 3.5)
	6/2	-4.0	(-6.2 to -1.9)	-4.4	(-6.6 to -2.2)	0.37	(-2.7 to 3.5)
	10 Follow-up	-3.4	(-5.9 to -1.0)	-2.8	(-5.2 to -0.25)	-0.70	(-4.2 to 2.8)
Missing, adjusted	2	-2.7	(-4.2 to -1.3)	-1.8	(-3.3 to -0.27)	-0.95	(-3.1 to 1.2)
	4	-3.4	(-5.2 to -1.7)	-3.3	(-5.1 to -1.5)	-0.12	(-2.6 to 2.4)
	6/1	-3.9	(-5.9 to -1.8)	-4.5	(-6.5 to -2.4)	0.63	(-2.3 to 3.5)
	6/2	-4.0	(-6.2 to -1.8)	-4.4	(-6.6 to -2.1)	0.39	(-2.8 to 3.6)
	10 Follow-up	-3.4	(-5.9 to -0.92)	-2.7	(-5.3 to -0.17)	-0.68	(-4.3 to 2.9)
Missing, not adjusted	2	-3.1	(-4.6 to -1.6)	-1.7	(-3.3 to -0.20)	-1.3	(-3.5 to 0.81)
	4	-3.8	(-5.6 to -1.9)	-3.3	(-5.1 to -1.4)	-0.53	(-3.1 to 2.1)
	6/1	-4.1	(-6.3 to -1.8)	-4.4	(-6.7 to -2.1)	0.33	(-2.9 to 3.6)
	6/2	-4.2	(-6.6 to -1.7)	-4.3	(-6.8 to -1.8)	0.11	(-3.4 to 3.6)
	10 Follow-up	-3.6	(-6.3 to -0.88)	-2.6	(-5.4 to 0.1)	-0.94	(-4.8 to 2.9)

Secondary efficacy outcomes determined by Linear Mixed effects Models (MMRM) on the intention to treat data. LSM, least square mean change from baseline; CI, Confidence interval; MNAR, missing not at random; MAR, missing at random. Model specifications were as follows: MNAR = adjusted for covariates, missing data imputed not at random and at random (see method section of primary paper); MAR = adjusted for covariates, all missing data imputed as MAR; Missing adjusted = no imputations of missing data, adjusted for covariates; Missing not adjusted = no imputations of missing data and no adjustment for covariates. Time point 6/1 equals treatment day 36; 6/2 equals treatment day 40; Follow-up was at week 10 for subjects completing the study and 2-4 weeks after the last dose for dropouts.

eTable 12. MMRM of the Conners' Adult ADHD Rating Scale F (*DSM-IV* Hyperactive-Impulsive Symptoms)

		**LSD (*n*=27)**		**Placebo (*n*=26)**		**Difference**	
Model	Week	LSM	(95% CI)	LSM	(95% CI)	LSM	(95% CI)
MNAR	2	-1.3	(-2.8 to 0.13)	-2.1	(-3.6 to -0.58)	0.75	(-1.4 to 2.9)
	4	-2.4	(-3.8 to -0.92)	-3.3	(-4.8 to -1.9)	0.98	(-1.1 to 3.1)
	6/1	-2.9	(-4.5 to -1.3)	-3.7	(-5.4 to -2.1)	0.86	(-1.4 to 3.2)
	6/2	-3.5	(-5.2 to -1.7)	-3.3	(-5.1 to -1.6)	-0.13	(-2.6 to 2.4)
	10 Follow-up	-2.8	(-4.4 to -1.3)	-3.1	(-4.7 to -1.5)	0.26	(-2.0 to 2.5)
MAR	2	-1.3	(-2.8 to 0.17)	-2.1	(-3.6 to -0.57)	0.79	(-1.4 to 2.9)
	4	-2.3	(-3.7 to -0.80)	-3.3	(-4.8 to -1.8)	1.1	(-1.1 to 3.2)
	6/1	-2.8	(-4.4 to -1.2)	-3.7	(-5.4 to -2.1)	0.95	(-1.4 to 3.3)
	6/2	-3.5	(-5.2 to -1.8)	-3.3	(-5.1 to -1.6)	-0.19	(-2.7 to 2.3)
	10 Follow-up	-2.8	(-4.4 to -1.3)	-3.1	(-4.7 to -1.5)	0.24	(-2.0 to 2.5)
Missing, adjusted	2	-1.4	(-2.9 to 0.089)	-2.2	(-3.8 to -0.65)	0.79	(-1.4 to 3.0)
	4	-2.4	(-3.9 to -0.89)	-3.4	(-5.0 to -1.9)	1.1	(-1.1 to 3.2)
	6/1	-2.9	(-4.5 to -1.2)	-3.8	(-5.5 to -2.1)	0.95	(-1.4 to 3.3)
	6/2	-3.6	(-5.4 to -1.9)	-3.4	(-5.2 to -1.6)	-0.18	(-2.7 to 2.4)
	10 Follow-up	-3.0	(-4.5 to -1.4)	-3.2	(-4.8 to -1.6)	0.25	(-2.1 to 2.5)
Missing, not adjusted	2	-2	(-3.7 to -0.31)	-1.5	(-3.2 to 0.19)	-0.5	(-2.9 to 1.9)
	4	-2.9	(-4.5 to -1.3)	-2.8	(-4.4 to -1.2)	-0.12	(-2.4 to 2.1)
	6/1	-3.4	(-5.2 to -1.7)	-3.2	(-4.9 to -1.4)	-0.22	(-2.7 to 2.3)
	6/2	-4.1	(-6.1 to -2.1)	-2.8	(-4.8 to -0.75)	-1.3	(-4.2 to 1.5)
	10 Follow-up	-3.4	(-5.3 to -1.5)	-2.6	(-4.5 to -0.63)	-0.85	(-3.6 to 1.9)

Secondary efficacy outcomes determined by Linear Mixed effects Models (MMRM) on the intention to treat data. LSM, least square mean change from baseline; CI, Confidence interval; MNAR, missing not at random; MAR, missing at random. Model specifications were as follows: MNAR = adjusted for covariates, missing data imputed not at random and at random (see method section of primary paper); MAR = adjusted for covariates, all missing data imputed as MAR; Missing adjusted = no imputations of missing data, adjusted for covariates; Missing not adjusted = no imputations of missing data and no adjustment for covariates. Time point 6/1 equals treatment day 36; 6/2 equals treatment day 40; Follow-up was at week 10 for subjects completing the study and 2-4 weeks after the last dose for dropouts.

eTable 13. MMRM of the Conners' Adult ADHD Rating Scale G (*DSM-IV* ADHD Symptoms Total)

		**LSD (*n*=27)**		**Placebo (*n*=26)**		**Difference**	
Model	Week	LSM	(95% CI)	LSM	(95% CI)	LSM	(95% CI)
MNAR	2	-3.9	(-6.3 to -1.5)	-4.0	(-6.5 to -1.6)	0.19	(-3.3 to 3.7)
	4	-5.5	(-8.3 to -2.6)	-6.8	(-9.7 to -4.0)	1.4	(-2.7 to 5.5)
	6/1	-6.8	(-9.9 to -3.7)	-8.4	(-12 to -5.2)	1.6	(-2.9 to 6.0)
	6/2	-7.4	(-11 to -4.1)	-8.0	(-11 to -4.6)	0.62	(-4.2 to 5.4)
	10 Follow-up	-6.4	(-9.9 to -2.9)	-6.0	(-9.6 to -2.4)	-0.38	(-5.4 to 4.7)
MAR	2	-3.8	(-6.2 to -1.4)	-4.1	(-6.5 to -1.6)	0.23	(-3.3 to 3.8)
	4	-5.3	(-8.2 to -2.4)	-6.9	(-9.8 to -3.9)	1.6	(-2.6 to 5.7)
	6/1	-6.5	(-9.7 to -3.4)	-8.4	(-12 to -5.2)	1.9	(-2.7 to 6.4)
	6/2	-7.3	(-11 to -4.0)	-8.1	(-11 to -4.6)	0.74	(-4.1 to 5.6)
	10 Follow-up	-6.4	(-9.9 to -2.9)	-6.0	(-9.6 to -2.4)	-0.38	(-5.4 to 4.7)
Missing, adjusted	2	-3.9	(-6.4 to -1.5)	-4.2	(-6.7 to -1.6)	0.24	(-3.3 to 3.8)
	4	-5.4	(-8.3 to -2.4)	-6.9	(-9.9 to -4.0)	1.6	(-2.7 to 5.8)
	6/1	-6.6	(-9.8 to -3.4)	-8.5	(-12 to -5.2)	1.9	(-2.7 to 6.5)
	6/2	-7.4	(-11 to -4.0)	-8.2	(-12 to -4.7)	0.77	(-4.2 to 5.7)
	10 Follow-up	-6.5	(-10 to -2.9)	-6.1	(-9.8 to -2.4)	-0.38	(-5.5 to 4.8)
Missing, not adjusted	2	-5.1	(-7.8 to -2.3)	-3.2	(-6.0 to -0.47)	-1.8	(-5.8 to 2.1)
	4	-6.7	(-9.6 to -3.7)	-6.0	(-9.0 to -3.1)	-0.62	(-4.8 to 3.6)
	6/1	-7.5	(-11 to -4.0)	-7.6	(-11 to -4.0)	0.065	(-4.9 to 5.1)
	6/2	-8.3	(-12 to -4.3)	-7.2	(-11 to -3.3)	-1.1	(-6.7 to 4.5)
	10 Follow-up	-7.3	(-11 to -3.1)	-5.2	(-9.4 to -1.0)	-2.1	(-7.9 to 3.8)

Secondary efficacy outcomes determined by Linear Mixed effects Models (MMRM) on the intention to treat data. LSM, least square mean change from baseline; CI, Confidence interval; MNAR, missing not at random; MAR, missing at random. Model specifications were as follows: MNAR = adjusted for covariates, missing data imputed not at random and at random (see method section of primary paper); MAR = adjusted for covariates, all missing data imputed as MAR; Missing adjusted = no imputations of missing data, adjusted for covariates; Missing not adjusted = no imputations of missing data and no adjustment for covariates. Time point 6/1 equals treatment day 36; 6/2 equals treatment day 40; Follow-up was at week 10 for subjects completing the study and 2-4 weeks after the last dose for dropouts.

eTable 14. MMRM of the Conners' Adult ADHD Rating Scale H (ADHD Index)

		**LSD (*n*=27)**		**Placebo (*n*=26)**		**Difference**	
Model	Week	LSM	(95% CI)	LSM	(95% CI)	LSM	(95% CI)
MNAR	2	-3.6	(-5.5 to -1.8)	-3.3	(-5.2 to -1.4)	-0.31	(-3.1 to 2.4)
	4	-5.7	(-7.6 to -3.7)	-5.1	(-7.1 to -3.1)	-0.61	(-3.5 to 2.3)
	6/1	-6.2	(-8.5 to -3.8)	-6.2	(-8.6 to -3.9)	0.094	(-3.3 to 3.5)
	6/2	-6.0	(-8.2 to -3.7)	-5.8	(-8.1 to -3.5)	-0.19	(-3.4 to 3.0)
	10 Follow-up	-4.7	(-7.2 to -2.3)	-4.7	(-7.3 to -2.2)	-0.011	(-3.6 to 3.6)
MAR	2	-3.6	(-5.5 to -1.8)	-3.3	(-5.2 to -1.4)	-0.31	(-3.0 to 2.4)
	4	-5.8	(-7.7 to -3.8)	-5.1	(-7.1 to -3.1)	-0.69	(-3.6 to 2.2)
	6/1	-6.1	(-8.4 to -3.8)	-6.3	(-8.6 to -3.9)	0.14	(-3.2 to 3.5)
	6/2	-6.0	(-8.2 to -3.8)	-5.8	(-8.0 to -3.5)	-0.19	(-3.4 to 3.0)
	10 Follow-up	-4.7	(-7.2 to -2.2)	-4.7	(-7.3 to -2.2)	0.019	(-3.6 to 3.6)
Missing, adjusted	2	-3.7	(-5.6 to -1.8)	-3.4	(-5.3 to -1.4)	-0.30	(-3.1 to 2.5)
	4	-5.8	(-7.8 to -3.8)	-5.1	(-7.2 to -3.1)	-0.69	(-3.6 to 2.3)
	6/1	-6.1	(-8.5 to -3.8)	-6.3	(-8.7 to -3.9)	0.15	(-3.3 to 3.6)
	6/2	-6.0	(-8.3 to -3.7)	-5.8	(-8.1 to -3.5)	-0.18	(-3.5 to 3.1)
	10 Follow-up	-4.8	(-7.3 to -2.2)	-4.8	(-7.4 to -2.2)	0.018	(-3.7 to 3.7)
Missing, not adjusted	2	-4.5	(-6.5 to -2.6)	-2.5	(-4.4 to -0.55)	-2.0	(-4.8 to 0.72)
	4	-6.6	(-8.6 to -4.6)	-4.3	(-6.3 to -2.3)	-2.3	(-5.1 to 0.55)
	6/1	-6.9	(-9.4 to -4.4)	-5.5	(-8.0 to -3.0)	-1.4	(-4.9 to 2.1)
	6/2	-6.8	(-9.2 to -4.3)	-5.0	(-7.4 to -2.6)	-1.8	(-5.2 to 1.7)
	10 Follow-up	-5.5	(-8.1 to -2.9)	-4.0	(-6.6 to -1.3)	-1.5	(-5.3 to 2.2)

Secondary efficacy outcomes determined by Linear Mixed effects Models (MMRM) on the intention to treat data. LSM, least square mean change from baseline; CI, Confidence interval; MNAR, missing not at random; MAR, missing at random. Model specifications were as follows: MNAR = adjusted for covariates, missing data imputed not at random and at random (see method section of primary paper); MAR = adjusted for covariates, all missing data imputed as MAR; Missing adjusted = no imputations of missing data, adjusted for covariates; Missing not adjusted = no imputations of missing data and no adjustment for covariates. Time point 6/1 equals treatment day 36; 6/2 equals treatment day 40; Follow-up was at week 10 for subjects completing the study and 2-4 weeks after the last dose for dropouts.

eTable 15. Descriptive Statistics of Efficacy Outcomes (Change From Baseline)

		LSD		Placebo	
	Visit	Δ to Baseline	*n*	Δ to Baseline	*n*
**AISRS**					
	Week 2	-3.3 ± 3.6	26	-4.8 ± 3.2	26
	Week 6	-7.9 ± 6.3	23	-8.4 ± 8.2	23
	Week 10 Follow-up	-6.6 ± 7.4	27	-5.8 ± 7.7	26
**ASRS**					
	Week 2	-8.1 ± 8.9	26	-6.7 ± 8.0	26
	Week 4	-10 ± 9.2	22	-9.0 ± 8.9	23
	Week 6/1	-13 ± 12	22	-11 ± 9.8	23
	Week 6/2	-15 ± 12	23	-12 ± 11	23
	Week 10 Follow-up	-13 ± 13	27	-8.9 ± 11	26
**CAARS**					
A=Inattention/Memory Problems					
	Week 2	-3.6 ± 4.3	26	-3.1 ± 5.5	26
	Week 4	-6.0 ± 4.3	22	-6.0 ± 4.9	23
	Week 6/1	-5.9 ± 5.8	22	-7.2 ± 6.3	23
	Week 6/2	-6.7 ± 4.7	23	-6 ± 7	23
	Week 10 Follow-up	-4.8 ± 6.6	27	-4.9 ± 7.2	26
B=Hyperactivity/Restlessness					
	Week 2	-3.1 ± 5.5	26	-3.3 ± 5.9	26
	Week 4	-5.1 ± 5.6	22	-4.6 ± 6.9	23
	Week 6/1	-6.0 ± 5.5	22	-5.8 ± 6.4	23
	Week 6/2	-6.8 ± 5.1	23	-4.5 ± 7.5	23
	Week 10 Follow-up	-5.3 ± 5.0	27	-4.2 ± 7.6	26
C=Impulsivity/Emotional Lability					
	Week 2	-5.3 ± 6.7	26	-1.8 ± 6.7	26
	Week 4	-7.1 ± 5.9	22	-4.4 ± 6.5	23
	Week 6/1	-7.0 ± 8.5	22	-5.1 ± 7.0	23
	Week 6/2	-7.2 ± 7.4	23	-5.4 ± 6.7	23
	Week 10 Follow-up	-5.7 ± 7.3	27	-3.4 ± 6.1	26
D=Problems with Self-Concept					
	Week 2	-2.8 ± 4.1	26	-1.4 ± 2.7	26
	Week 4	-3.8 ± 4.1	22	-1.7 ± 3.1	23
	Week 6/1	-3.5 ± 4.6	22	-2.4 ± 3.2	23
	Week 6/2	-3.6 ± 4.3	23	-2 ± 3.4	23
	Week 10 Follow-up	-2.7 ± 4.6	27	-1.4 ± 3.5	26
E=DSM-IV Inattentive Symptoms					
	Week 2	-3.1 ± 4.8	26	-1.7 ± 2.7	26
	Week 4	-4.4 ± 4.2	22	-3.3 ± 4.6	23
	Week 6/1	-4.8 ± 5.6	22	-4.4 ± 5.3	23
	Week 6/2	-4.9 ± 6.0	23	-4.3 ± 5.8	23
	Week 10 Follow-up	-3.6 ± 6.8	27	-2.8 ± 5.7	26
F=DSM-IV Hyperactive-Impulsive Symptoms					
	Week 2	-2 ± 4.0	26	-1.5 ± 4.5	26
	Week 4	-3.3 ± 3.8	22	-2.9 ± 3.9	23
	Week 6/1	-3.7 ± 4.6	22	-3.3 ± 3.9	23
	Week 6/2	-4.6 ± 5.2	23	-2.9 ± 4.6	23
	Week 10 Follow-up	-3.2 ± 4.6	27	-2.2 ± 5.0	26
G=DSM-IV ADHD Symptoms Total					
	Week 2	-5.1 ± 8.0	26	-3.2 ± 5.9	26
	Week 4	-7.7 ± 6.7	22	-6.1 ± 7.2	23
	Week 6/1	-8.5 ± 9.0	22	-7.7 ± 7.9	23
	Week 6/2	-9.5 ± 9.9	23	-7.3 ± 8.9	23
	Week 10 Follow-up	-7.0 ± 10	27	-5.0 ± 9.1	26

**CAARS**				
H=ADHD Index				
Week 2	-4.5 ± 5.4	26	-2.5 ± 4.4	26
Week 4	-6.9 ± 4.6	22	-4.5 ± 4.9	23
Week 6/1	-7.1 ± 6.2	22	-5.7 ± 6.0	23
Week 6/2	-7.0 ± 5.7	23	-5.2 ± 6.0	23
Week 10 Follow-up	-5.3 ± 6.7	27	-3.4 ± 6.3	26
**CGI-S**				
Absolute values				
Week 2	-0.3 ± 0.5	26	-0.4 ± 0.5	26
Week 6	-0.9 ± 0.7	23	-1.0 ± 1.0	23
Week 10 Follow-up	-0.8 ± 0.8	27	-0.7 ± 0.9	26
Fraction with improvement >= 1				
Week 2	0.3 ± 0.5	26	0.4 ± 0.5	26
Week 6	0.7 ± 0.5	23	0.7 ± 0.5	23
Week 10 Follow-up	0.6 ± 0.5	27	0.5 ± 0.5	26

AISRS, Adult ADHD Investigator Symptom Rating Scale; ASRS, ADHD Self-Report Scale; CAARS, Conners' Adult ADHD Rating Scale with 8 subscales; CGI-S, Clinical Global Impression-severity; data are mean ± standard deviation differences to baseline values. Week 6/1 equals treatment day 36; week 6/2 equals treatment day 40; Follow-up was during week 10 for persons completing the study and 2-4 weeks after the last dose for dropouts.

eTable 16. Descriptive Statistics of Efficacy Outcomes (Absolute Values)

	Visit	LSD		Placebo	
		mean±SD	*n*	mean±SD	*n*
**AISRS**					
	Baseline	37 ± 5.8	27	37 ± 5.1	26
	Week 2	34 ± 6.5	26	32 ± 5.0	26
	Week 6/2	30 ± 8.3	23	29 ± 8.5	23
	Week 10 Follow-up	31 ± 8.9	27	31 ± 8.2	26
**ASRS**					
	Baseline	47 ± 8.1	27	44 ± 7.3	26
	Week 2	39 ± 9.5	26	37 ± 7.5	26
	Week 4	37 ± 8.2	22	35 ± 11	23
	Week 6/1	35 ± 9.6	22	33 ± 12	23
	Week 6/2	33 ± 9.1	23	32 ± 12	23
	Week 10 Follow-up	34 ± 10	27	35 ± 12	26
**CAARS**					
A=Inattention/Memory Problems					
	Baseline	23 ± 5.2	27	22 ± 6.3	26
	Week 2	20 ± 6.2	26	19 ± 6.2	26
	Week 4	17 ± 5.8	22	16 ± 6.1	23
	Week 6/1	18 ± 5.2	22	15 ± 6.4	23
	Week 6/2	17 ± 5.3	23	16 ± 7.5	23
	Week 10 Follow-up	18 ± 7.4	27	17 ± 6.6	26
B=Hyperactivity/Restlessness					
	Baseline	23 ± 7.1	27	20 ± 6.0	26
	Week 2	20 ± 6.3	26	16 ± 5.9	26
	Week 4	17 ± 5.4	22	15 ± 7.3	23
	Week 6/1	17 ± 6.0	22	14 ± 6.4	23
	Week 6/2	16 ± 5.6	23	15 ± 7.8	23
	Week 10 Follow-up	17 ± 5.9	27	16 ± 6.6	26

**CAARS**				
C=Impulsivity/Emotional Lability				
Baseline	20 ± 7.0	27	17 ± 6.2	26
Week 2	15 ± 6.8	26	16 ± 6.3	26
Week 4	12 ± 4.4	22	14 ± 5.8	23
Week 6/1	13 ± 7.3	22	13 ± 5.6	23
Week 6/2	12 ± 5.4	23	13 ± 5.5	23
Week 10 Follow-up	14 ± 7.4	27	14 ± 6.3	26
D=Problems with Self-Concept				
Baseline	13 ± 4.2	27	11 ± 4.3	26
Week 2	10 ± 4.4	26	9.5 ± 4.2	26
Week 4	8.4 ± 4.5	22	9.7 ± 4.7	23
Week 6/1	8.8 ± 5.2	22	8.9 ± 5.1	23
Week 6/2	8.8 ± 4.7	23	9.3 ± 5.0	23
Week 10 Follow-up	10 ± 5.4	27	9.5 ± 4.8	26
E=DSM-IV Inattentive Symptoms				
Baseline	18 ± 4.3	27	17 ± 3.4	26
Week 2	15 ± 5.1	26	15 ± 3.7	26
Week 4	13 ± 4.3	22	14 ± 4.5	23
Week 6/1	13 ± 4.5	22	12 ± 5.2	23
Week 6/2	13 ± 4.5	23	13 ± 5.6	23
Week 10 Follow-up	14 ± 5.8	27	14 ± 5.2	26
F=DSM-IV Hyperactive-Impulsive Symptoms				
Baseline	14 ± 5.5	27	12 ± 5.0	26
Week 2	13 ± 5.0	26	11 ± 4.4	26
Week 4	11 ± 4.4	22	9.9 ± 4.9	23
Week 6/1	11 ± 5.0	22	9.5 ± 5.3	23
Week 6/2	9.8 ± 4.5	23	9.9 ± 5.2	23
Week 10 Follow-up	11 ± 4.4	27	10 ± 4.3	26
G=DSM-IV ADHD Symptoms Total				
Baseline	32 ± 8.0	27	29 ± 5.4	26
Week 2	27 ± 7.9	26	26 ± 5.4	26
Week 4	24 ± 6.7	22	23 ± 7.4	23
Week 6/1	23 ± 7.9	22	22 ± 8.8	23
Week 6/2	23 ± 7.4	23	22 ± 8.8	23
Week 10 Follow-up	25 ± 8.7	27	24 ± 8.3	26
H=ADHD Index				
Baseline	23 ± 5.2	27	21 ± 4.1	26
Week 2	19 ± 6.3	26	18 ± 4.1	26
Week 4	16 ± 5.0	22	17 ± 5.1	23
Week 6/1	16 ± 6.1	22	15 ± 5.9	23
Week 6/2	16 ± 5.4	23	16 ± 5.9	23
Week 10 Follow-up	18 ± 6.9	27	17 ± 5.9	26
**CGI-S**				
Baseline	4.9 ± 0.7	27	4.7 ± 0.6	26
Week 2	4.6 ± 0.7	26	4.3 ± 0.5	26
Week 6/2	4.0 ± 0.9	23	3.8 ± 0.9	23
Week 10 Follow-up	4.1 ± 0.9	27	4 ± 0.9	26

AISRS, Adult ADHD Investigator Symptom Rating Scale; ASRS, ADHD Self-Report Scale; CAARS, Conners' Adult ADHD Rating Scale with 8 subscales; CGI-S, Clinical Global Impression-severity; reported are mean values ± standard deviation according to treatment. Week 6/1 equals treatment day 36; week 6/2 equals treatment day 40; End of study was during week 10 for persons completing the study and 2-4 weeks after the last dose for dropouts.

eFigure 1. AISRS, ASRS, MMRM

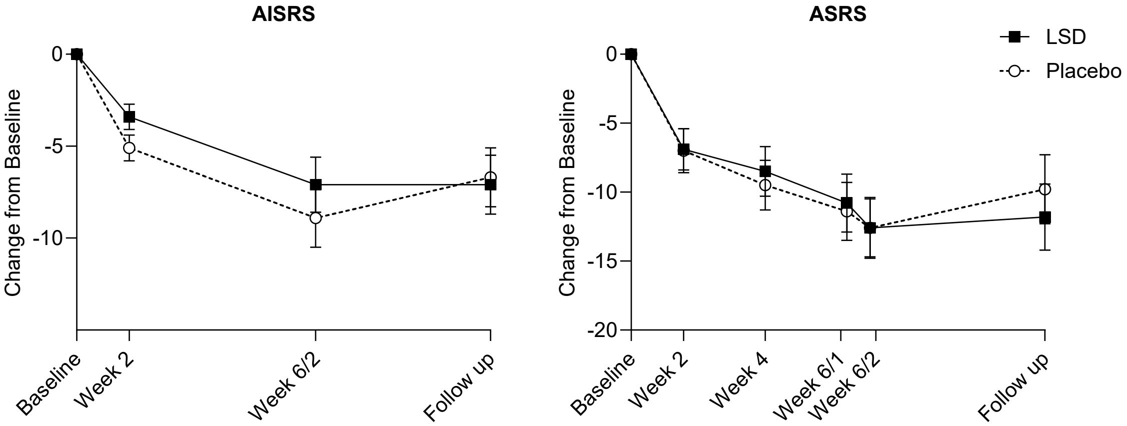

eFigure 1 Results of the Mixed Effects Models for Repeated Measures (MRMM) adjusted for covariates, using the missing not at random approach for imputation of missing data and all available time points. Data are least square mean and standard error of the mean change from baseline values. AISRS, Adult ADHD Investigator Symptom Rating Scale; ASRS, ADHD Self-Report Scale; week 6/1 equals treatment day 36; week 6/2 equals treatment day 40; *n* LSD = 27, *n* Placebo = 26.

eFigure 2. Clinical Global Impression-Severity

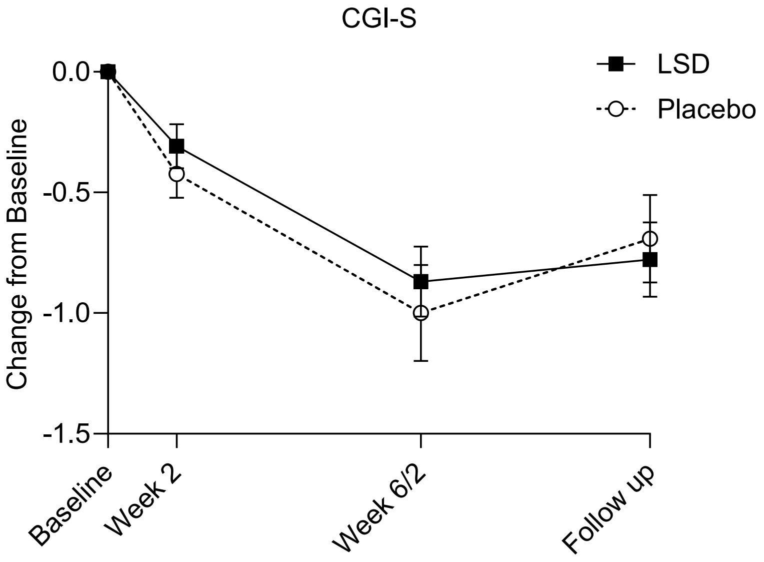

eFigure 2 Clinical Global Impression-severity (CGI-S) scale over time. Data are arithmetic mean and standard error of the mean difference from baseline values.
For *n* see eTable 15.

eFigure 3. Conners' Adult ADHD Rating Scale MMRM

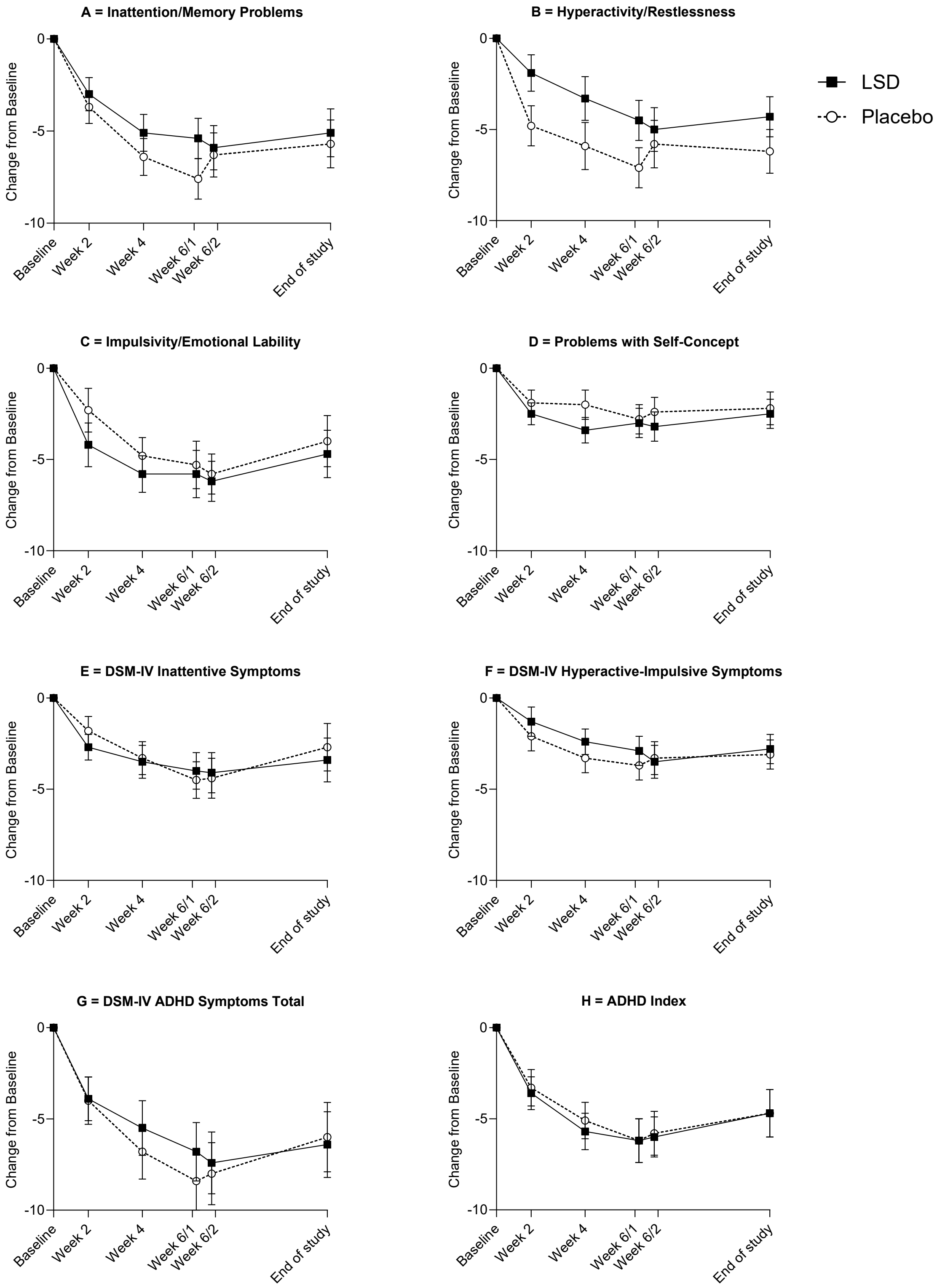

eFigure 3 Results of the Mixed Effects Models for Repeated Measures (MMRM) adjusted for covariates, using the missing not at random approach for imputation of missing data and all available time points. All subscales of the Conners' Adult ADHD Rating Scale are shown. Data are least square mean and standard error of the mean change from baseline values. Week 6/1 equals treatment day 36; week 6/2 equals treatment day 40; *n* LSD = 27, *n* Placebo = 26.

eFigure 4. Outcomes After 6 Weeks Stratified by Guessed Allocation MMRM

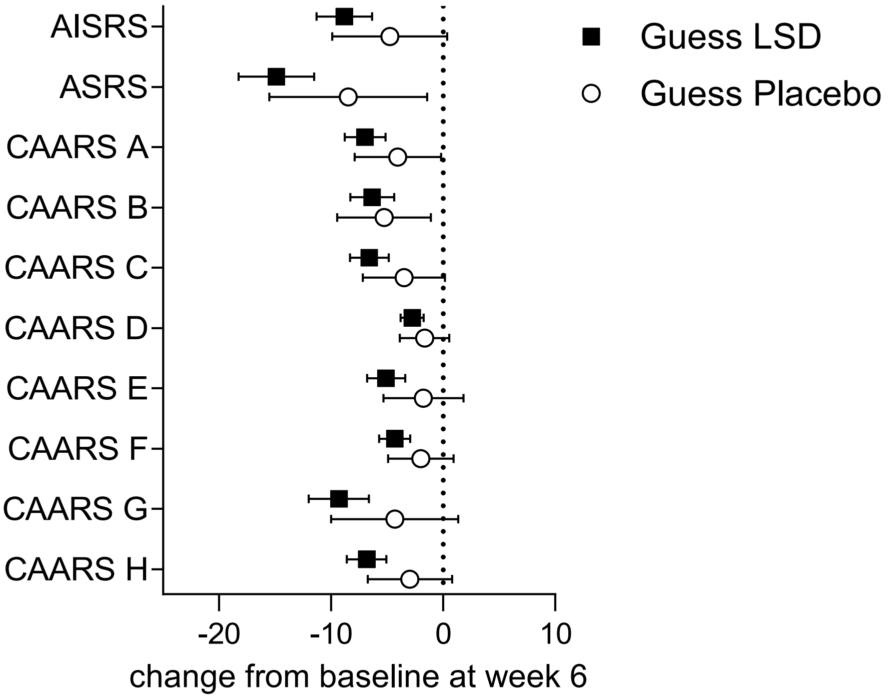

eFigure 4 Illustration of potential expectancy bias. The same Mixed Effects Models for Repeated Measures (MMRM) adjusted for covariates, using the missing not at random approach for imputation of missing data and all available time points as for other secondary endpoints was applied with the following changes: Instead of the actual allocation we used the participants guessed allocation after 6 weeks of treatment. Data are expressed as least square mean changes from baseline with error bars representing the 95% confidence intervals. Group sizes (not including dropouts) were the following: *n* “Guess LSD” = 35, *n* “Guess Placebo” = 8. AISRS, Adult ADHD Investigator Symptom Rating Scale; ASRS, Adult ADHD Self-Report Scale; CAARS, Conners' Adult ADHD Rating Scale. The blinding data was assessed only at the Basel site in addition to the per protocol measures.

eTable 17. Acute Effects Measured by Visual Analog Scales

**Emax**		**LSD (*n*=27)**	**Placebo (*n*=26)**	**t**	**p**
	Any drug effect	52 ± 33	22 ± 29	3.5	***
	Good drug effect	56 ± 35	32 ± 33	2.6	*
	Bad drug effect	13 ± 23	1.6 ± 4.1	2.5	*
	Ego dissolution	17 ± 27	2.9 ± 7.5	2.6	*
	Fear	6.3 ± 17	5.4 ± 14	0.2	NS
	Nausea	13 ± 20	1.1 ± 4.5	2.8	**
	Alteration of vision	20 ± 26	11 ± 23	1.3	NS
	Altered sense of time	19 ± 29	14 ± 22	0.7	NS
**AUEC**					
	Any drug effect	175 ± 137	70 ± 112	3.1	**
	Good drug effect	228 ± 183	124 ± 160	2.2	*
	Bad drug effect	31 ± 60	2.9 ± 10	2.4	*
	Ego dissolution	43 ± 84	3.6 ± 11	2.4	*
	Fear	15 ± 52	3.6 ± 10	1.2	NS
	Nausea	32 ± 65	1.0 ± 4	2.5	*
	Alteration of vision	51 ± 86	32 ± 81	0.9	NS
	Altered sense of time	55 ± 106	27 ± 53	1.2	NS

Acute subjective measured by visual analog scales assessed repeatedly over the observation period of 6 hours; Emax, maximal effect; AUEC, area under the effect-time curve; Values are mean ± standard deviation in % of maximal possible values; t and p values refer to two-sided t-test for independent groups. *P<0.05, **P<0.01, ***P<0.001.

eFigure 5. Acute Effects Measured by Visual Analog Scales

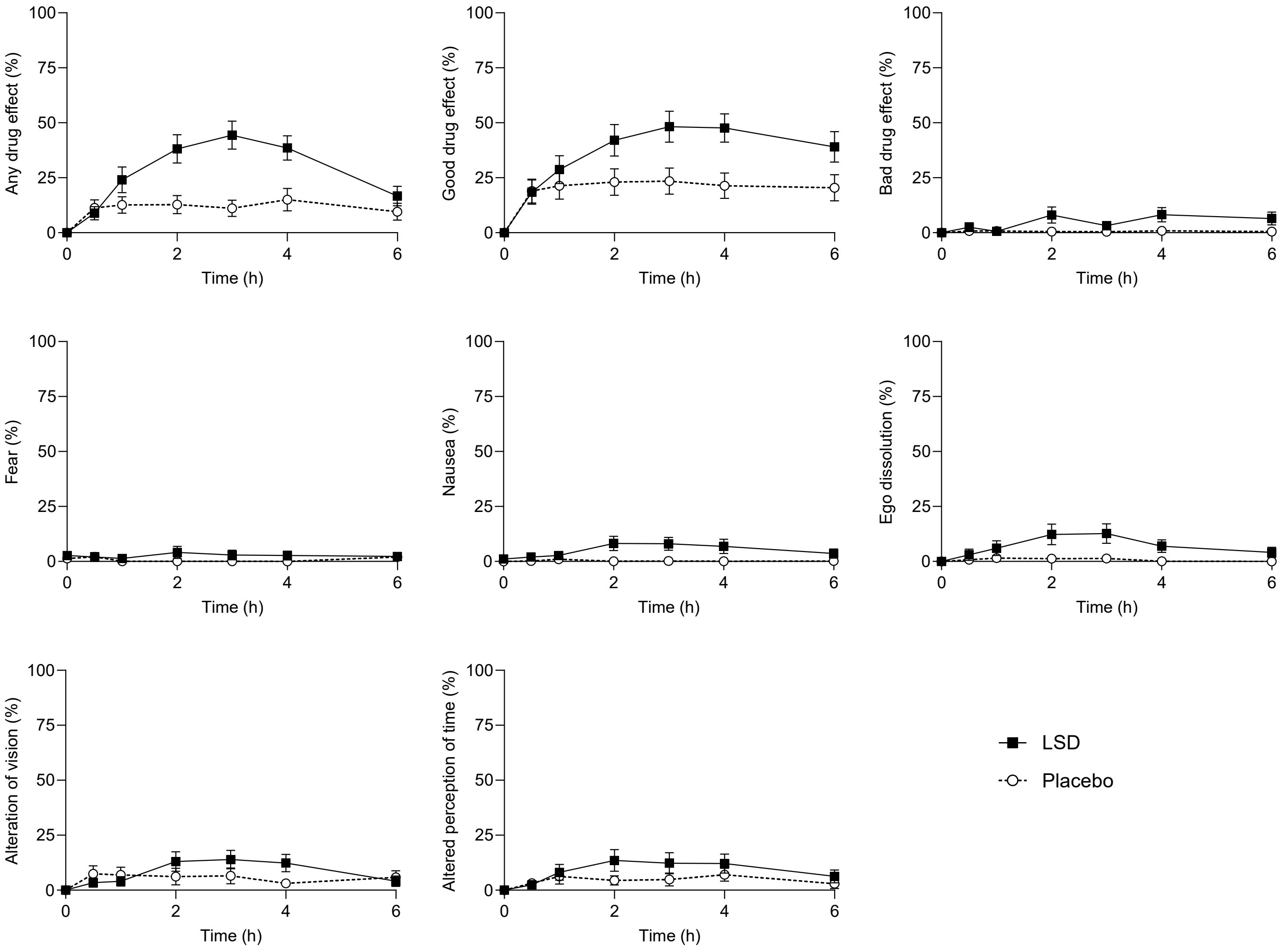

eFigure 5 Data are mean with standard error of the mean as error bars;
n LSD = 27;
n Placebo = 26.

eFigure 6. Acute Effects Measured by Visual Analog Scales (Spaghetti Plots)

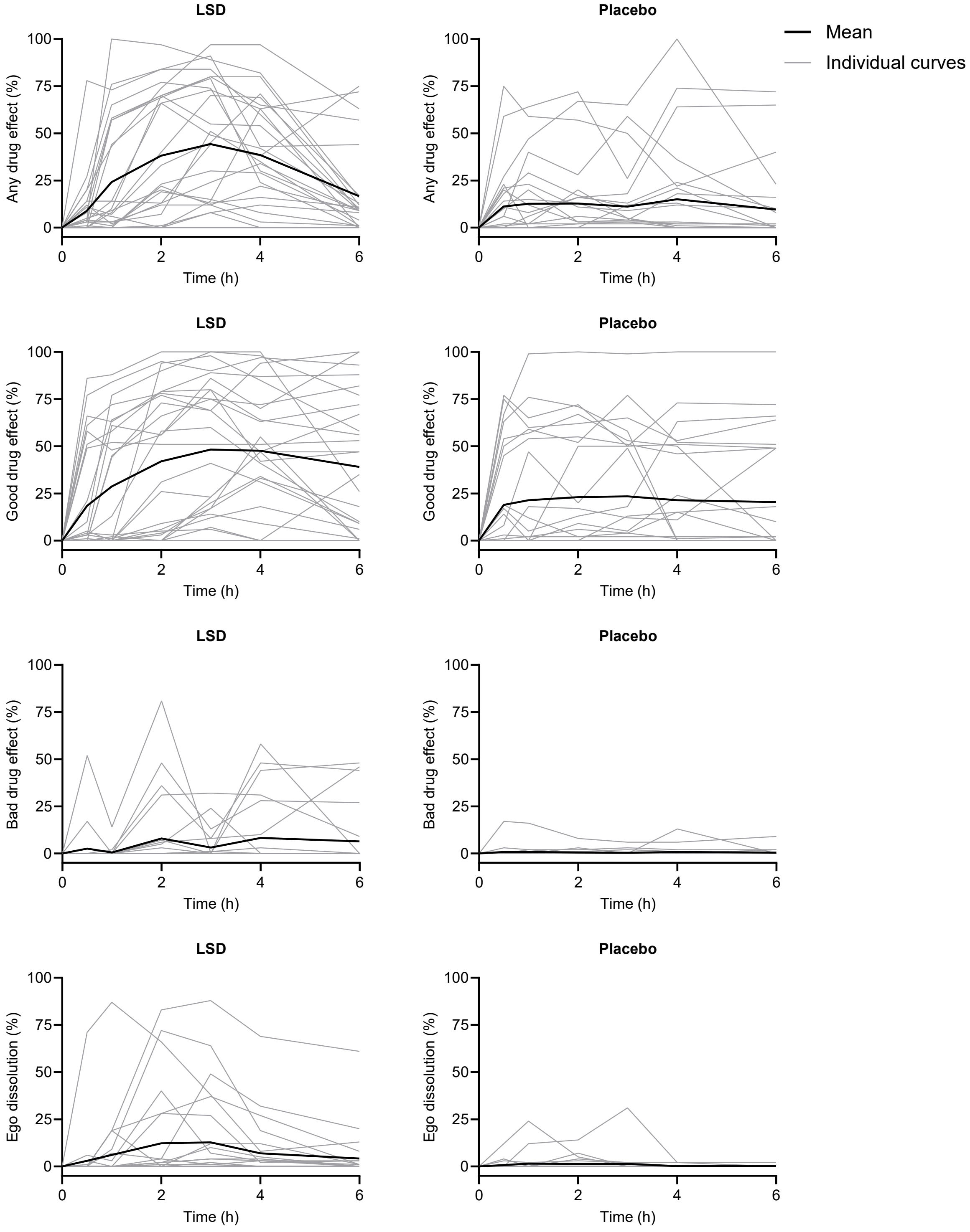

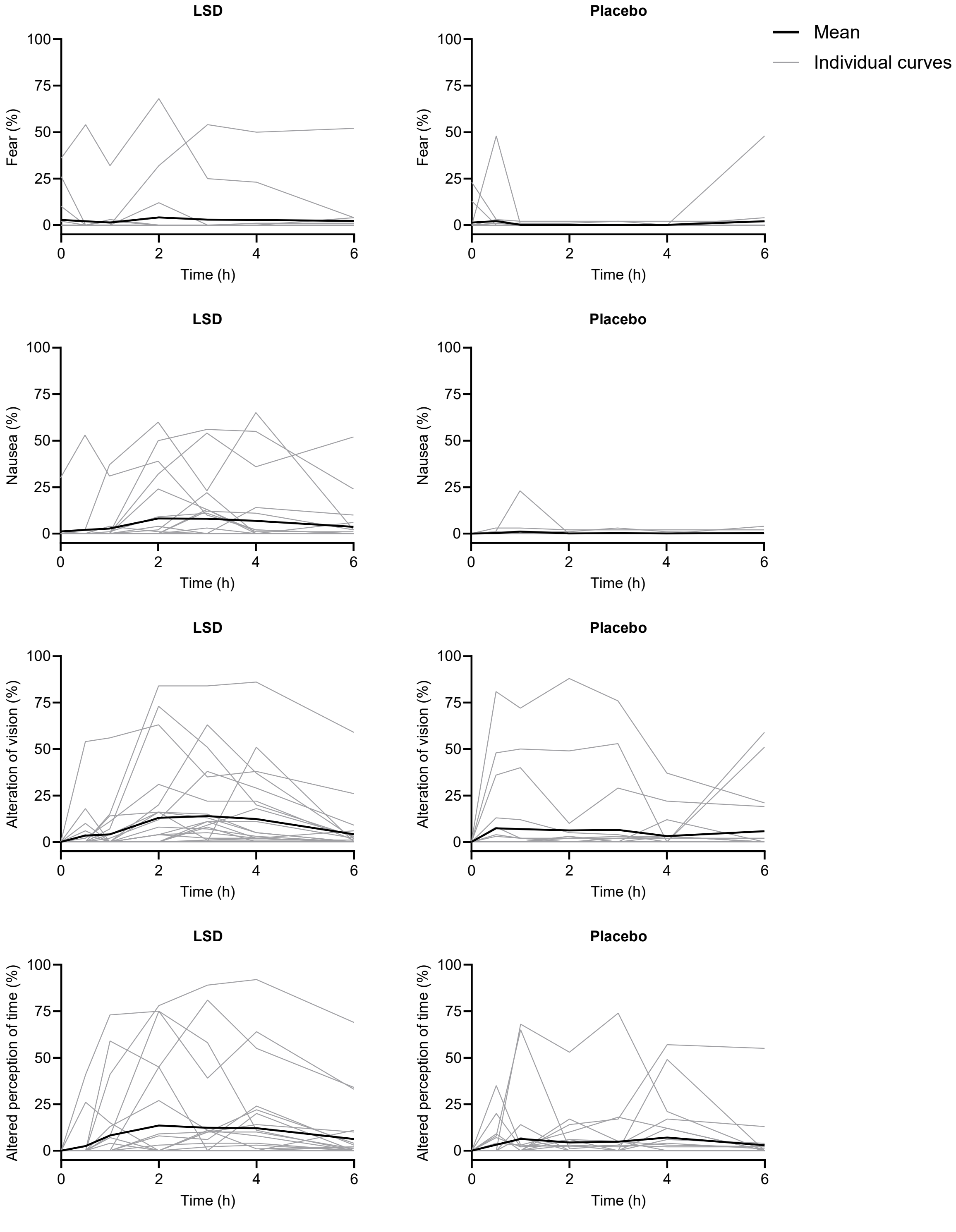

eFigure 6 Each grey line represents an individual subject; the bold line represents the mean; *n* LSD = 27; *n* Placebo = 26.

eTable 18. Acute Effects on the 5 Dimensions of Altered States of Consciousness Scale and Mystical Experiences Questionnaire

	After the first dose				After the last dose			
	LSD (*n*=27)	Placebo (*n*=26)			LSD (*n*=23)	Placebo (*n*=23)		
	% score ± SD	% score ± SD	t	p	% score ± SD	% score ± SD	t	p
5 Dimensions of Altered States of Consciousness scale								
Oceanic boundlessness	18 ± 19	6.0 ± 12	2.7	*	15 ± 18	2.6 ± 4.6	3.2	**
Anxious ego-dissolution	4.9 ± 8.2	0.91 ± 1.5	2.5	*	2.0 ± 4.6	0.2 ± 0.5	1.8	NS
Visionary restructuralization	13 ± 14	5.3 ± 10	2.3	*	11 ± 14	1.8 ± 3.4	3.0	**
3D-ASC total score	13 ± 12	4.2 ± 8.2	2.9	**	9.9 ± 12	1.7 ± 2.8	3.3	**
Auditory alterations	4.4 ± 9.3	2.0 ± 4.8	1.2	NS	1.2 ± 2.6	0.26 ± 0.61	1.6	NS
Reductions of vigilance	24 ± 19	13 ± 16	2.3	*	7.5 ± 7.4	1.7 ± 2.5	3.6	**
5D-ASC total score	13 ± 11	4.9 ± 7.8	3.0	**	8.0 ± 9.0	1.4 ± 2.0	3.4	**
Experience of unity	13 ± 21	5.4 ± 13	1.7	NS	10 ± 18	1.5 ± 2.7	2.3	*
Spiritual experience	7.4 ± 16	1.8 ± 4.4	1.7	NS	7.5 ± 18	0.6 ± 1.1	1.8	NS
Blissful state	33 ± 33	12 ± 25	2.7	**	27 ± 34	7.4 ± 18	2.5	*
Insightfulness	18 ± 23	6.8 ± 17	2.0	NS	22 ± 27	3.3 ± 6.3	3.1	**
Disembodiment	4.8 ± 16	3.1 ± 9.9	0.5	NS	3.6 ± 7.8	0.3 ± 0.7	2.1	NS
Impaired control and cognition	7.1 ± 9.8	1.5 ± 3.4	2.7	**	2.5 ± 4.8	0.3 ± 0.5	2.2	*
Anxiety	3.7 ± 11	0.3 ± 0.7	1.6	NS	1.7 ± 5.9	0.2 ± 0.5	1.2	NS
Complex imagery	17 ± 19	8.5 ± 19	1.6	NS	19 ± 30	2.1 ± 4.3	2.7	*
Elementary imagery	12 ± 21	3.2 ± 11	1.9	NS	6.0 ± 14	1.5 ± 3.7	1.5	NS
Audio-Visual synesthesiae	3.7 ± 9.6	2.2 ± 6.0	0.7	NS	3.6 ± 13	0.1 ± 0.5	1.3	NS
Changed meaning of percepts	15 ± 20	3.9 ± 11	2.5	*	9.8 ± 12	1.5 ± 2.5	3.3	**
Mystical Experiences Questionnaire								
Mystical	19 ± 22	8 ± 15	2.1	*	19 ± 23	4.2 ± 6.3	2.9	**
Positive mood	34 ± 23	16 ± 20	3.1	**	31 ± 24	11 ± 15	3.4	**
Transcendence of time/space	17 ± 17	11 ± 17	1.5	NS	14 ± 15	3.5 ± 7.4	2.9	**
Ineffability	26 ± 27	12 ± 19	2.3	*	22 ± 26	5.8 ± 16	2.6	*
MEQ30 Total score	22 ± 20	10 ± 16	2.4	*	21 ± 20	5.6 ± 8.6	3.3	**

Retrospective assessment of acute effects during the first and last dose. 5D-ASC, 5 Dimensions of Altered States of Consciousness scale; 3D-ASC 3, Dimensions of Altered States of Consciousness scale; MEQ30, Mystical Experiences Questionnaire; SD, standard deviation; p and t values refer to two-sided t-test for independent groups.
*p<0.05, **p<0.01, ***p<0.001; NS, not significant.

eFigure 7. Acute Effects on the 5 Dimensions of Altered States of Consciousness Scale and Mystical Experiences Questionnaire

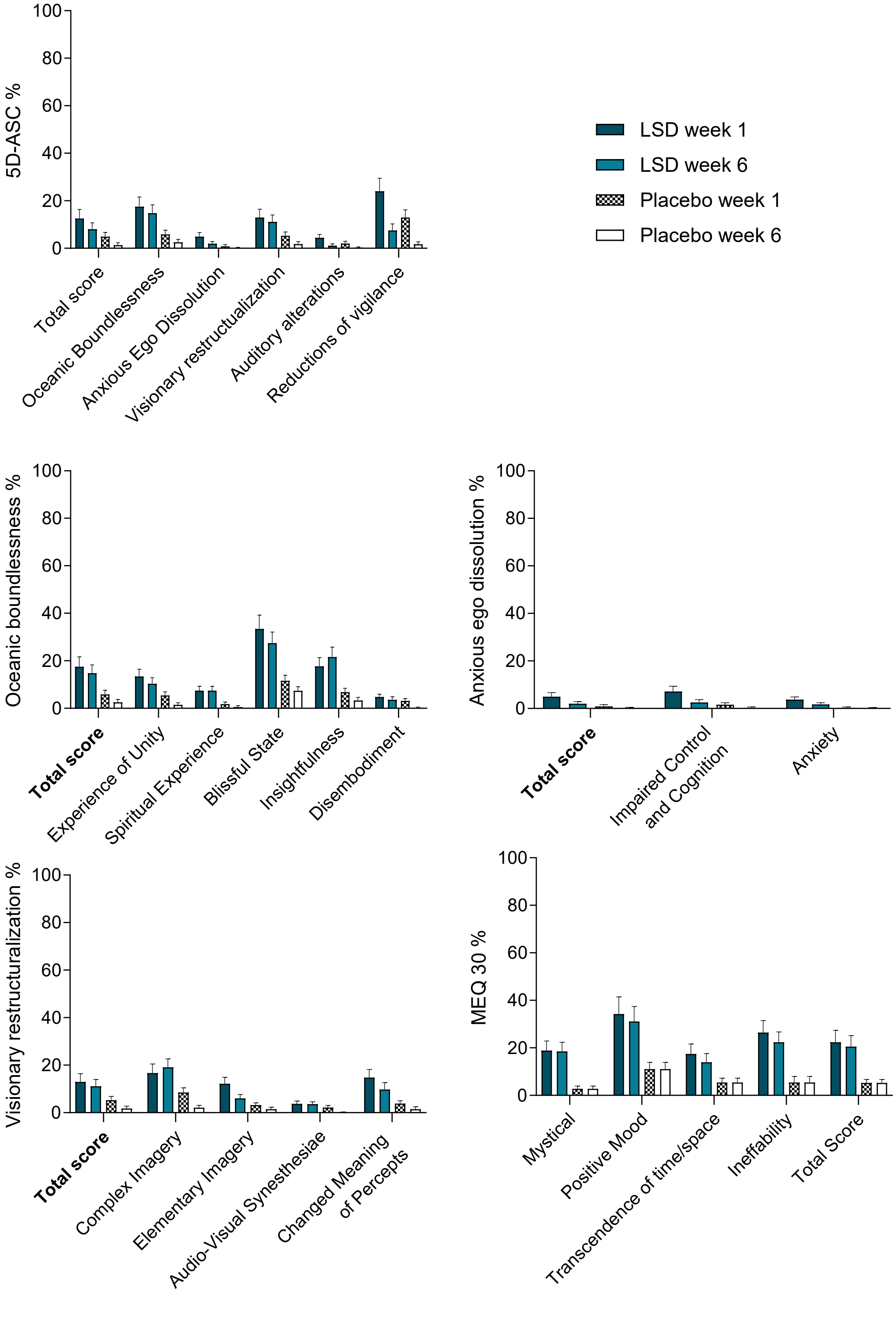

eFigure 7 5D-ASC, 5 Dimensions of Altered States of Consciousness scale; MEQ30, Mystical Experiences Questionnaire; Scores are percentages of maximal possible scores per factor/dimension. Data are mean and standard error of the mean. For *n* see eTable 17.

eTable 19. Pharmacokinetic Parameters of LSD (20 µg) Determined by Non-Compartmental Analysis

	C_{max} ng/L	t_{max} h	$t_{1/2}$ h	AUC_6 ng·h/L	AUC_∞ ng·h/L	CL/F L/h	V_z/F L
GEO mean	505	1.3	3.6	2060	3220	6.2	32
95% CI	(438-581)	(1.1-1.5)	(3.2-4.0)	(1790-2370)	(2680-3870)	(5.2-7.5)	(29-36)
range	246-908	0.58-3.1	2.0-6.4	1020-3700	1240-7640	2.6-16	19-58

C_{max}, maximum observed plasma concentration; t_{max}, time to reach C_{max}; $t_{1/2}$, plasma half-life; AUC_6, area under the plasma concentration-time curve from time 0 to 6 h; AUC_∞, AUC from time zero to infinity; CL/F, apparent total clearance; Vz/F, apparent volume of distribution; GEO mean, geometric mean; 95% CI, 95% confidence interval of the GEO mean; *n* = 27.

eFigure 8. Pharmacokinetics of LSD and O-H-LSD

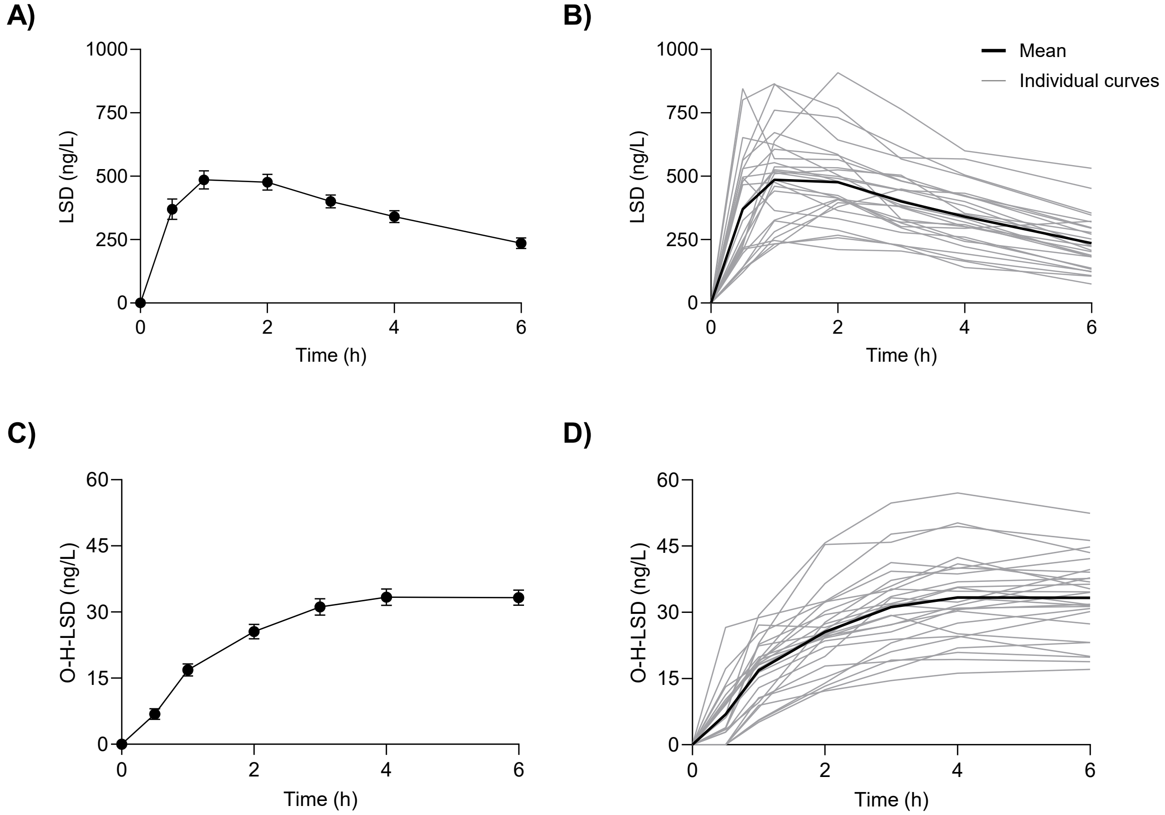

eFigure 8 A) mean LSD concentration with standard error of the mean as error bars; **B)** mean and individual LSD concentrations; **C)** mean O-H-LSD concentrations with standard error as error bars; **D)** mean and individual concentrations of O-H-LSD; *n* = 27.

eTable 20. Adverse Events

Overview	Unique count per subject and preferred term			Any count including duplicates		
	LSD (*n*=27)	Placebo (*n*=26)	Total (*n*=53)	LSD (*n*=27)	Placebo (*n*=26)	Total (*n*=53)
All adverse events	74	46	120	124	68	192
severity						
mild	54	26	80	94	38	132
moderate	20	20	40	30	30	60
severe	0	0	0	0	0	0
relation to study medication						
definitely	5	0	5	7	0	7
possibly	26	9	35	58	17	75
probably	13	1	14	18	1	19
unlikely	2	2	4	7	7	14
unrelated	28	34	62	34	43	77

According to system organ class and preferred term	Unique count per subject and preferred term			Any count including duplicates		
	LSD (*n*=27)	Placebo (*n*=26)	Total (*n*=53)	LSD (*n*=27)	Placebo (*n*=26)	Total (*n*=53)
Nervous system disorders	18	13	31	40	25	65
Headache	13	9	22	33	21	54
Disturbance in attention	2	2	4	3	2	5
Akathisia	1	1	2	1	1	2
Dizziness postural	0	1	1	0	1	1
Restless legs syndrome	1	0	1	1	0	1
Somnolence	1	0	1	2	0	2
Gastrointestinal disorders	10	8	18	23	10	33
Nausea	5	1	6	10	1	11
Abdominal pain	1	2	3	1	3	4
Abdominal pain upper	1	2	3	1	3	4
Diarrhoea	1	1	2	1	1	2
Flatulence	2	0	2	10	0	10
Abdominal pain lower	0	1	1	0	1	1
Vomiting	0	1	1	0	1	1
Psychiatric disorders	12	3	15	23	3	26
Illusion	4	0	4	12	0	12
Insomnia	3	1	4	5	1	6
Apathy	1	2	3	1	2	3
Euphoric mood	1	0	1	2	0	2
Flashbacks	1	0	1	1	0	1
Hypersomnia	1	0	1	1	0	1
Sleep disorder	1	0	1	1	0	1
Infections and infestations	5	6	11	5	9	14
Nasopharyngitis	5	5	10	5	8	13
Urinary tract infection	0	1	1	0	1	1
General disorders and administration site conditions	7	1	8	7	1	8
Fatigue	4	1	5	4	1	5
Feeling abnormal	2	0	2	2	0	2
Swelling	1	0	1	1	0	1
Injury, poisoning and procedural complications	4	1	5	4	1	5
Contusion	1	1	2	1	1	2
Ankle fracture	1	0	1	1	0	1
Palate injury	1	0	1	1	0	1
Wound	1	0	1	1	0	1

	Unique count per subject and preferred term			Any count including duplicates		
	LSD (*n*=27)	Placebo (*n*=26)	Total (*n*=53)	LSD (*n*=27)	Placebo (*n*=26)	Total (*n*=53)
Investigations	1	4	5	1	6	7
Full blood count abnormal	1	0	1	1	0	1
Heart rate increased	0	1	1	0	3	3
Pregnancy test positive	0	1	1	0	1	1
SARS-CoV-2 test positive	0	1	1	0	1	1
Urine cannabinoids positive	0	1	1	0	1	1
Respiratory, thoracic and mediastinal disorders	2	3	5	3	3	6
Oropharyngeal pain	0	2	2	0	2	2
Nasal congestion	1	0	1	1	0	1
Throat tightness	0	1	1	0	1	1
Epistaxis	1	0	1	2	0	2
Vascular disorders	4	0	4	4	0	4
Hot flush	2	0	2	2	0	2
Hypertension	2	0	2	2	0	2
Cardiac disorders	2	1	3	2	4	6
Palpitations	1	1	2	1	4	5
Syncope	1	0	1	1	0	1
Musculoskeletal and connective tissue disorders	2	1	3	3	1	4
Arthralgia	0	1	1	0	1	1
Back pain	1	0	1	1	0	1
Muscle tightness	1	0	1	2	0	2
Skin and subcutaneous tissue disorders	2	0	2	4	0	4
Hyperhidrosis	1	0	1	1	0	1
Rash	1	0	1	3	0	3
Social circumstances	2	0	2	2	0	2
High risk sexual behaviour	1	0	1	1	0	1
Substance use	1	0	1	1	0	1
Blood and lymphatic system disorders	0	1	1	0	1	1
Anaemia	0	1	1	0	1	1
Ear and labyrinth disorders	1	0	1	1	0	1
Ear pain	1	0	1	1	0	1
Eye disorders	1	0	1	1	0	1
Vision blurred	1	0	1	1	0	1
Hepatobiliary disorders	0	1	1	0	1	1
Liver disorder	0	1	1	0	1	1
Metabolism and nutrition disorders	1	0	1	1	0	1
Hypophosphataemia	1	0	1	1	0	1
Renal and urinary disorders	0	1	1	0	1	1
Urethritis noninfective	0	1	1	0	1	1
Reproductive system and breast disorders	0	1	1	0	1	1
Premenstrual pain	0	1	1	0	1	1
Surgical and medical procedures	0	1	1	0	1	1
Tooth extraction	0	1	1	0	1	1

Adverse events (AE) classification was done according to MedDRA® 25.1. Reported are unique preferred terms per subject (repeated occurences of the same AE within one participant are counted only once) and total counts (including duplicates). Classification was done according to MedDRA® 25.1.

eTable 21. Related Adverse Events

	Unique count per subject and preferred term			Any count including duplicates		
	LSD (*n*=27)	Placebo (*n*=26)	Total (*n*=53)	LSD (*n*=27)	Placebo (*n*=26)	Total (*n*=53)
Headache	10	7	17	21	12	33
Nausea	5	1	6	10	1	11
Fatigue	4	1	5	4	1	5
Insomnia	3	1	4	4	1	5
Illusion	3	0	3	11	0	11
Feeling abnormal	2	0	2	2	0	2
Flatulence	2	0	2	10	0	10
Hot flush	2	0	2	2	0	2
Hypertension	2	0	2	2	0	2
Apathy	0	1	1	0	1	1
Abdominal pain	0	1	1	0	2	2
Akathisia	1	0	1	1	0	1
Diarrhoea	1	0	1	1	0	1
Disturbance in attention	1	0	1	2	0	2
Euphoric mood	1	0	1	2	0	2
Flashbacks	1	0	1	1	0	1
Hyperhidrosis	1	0	1	1	0	1
Muscle tightness	1	0	1	2	0	2
Palpitations	1	0	1	1	0	1
Restless legs syndrome	1	0	1	1	0	1
Sleep disorder	1	0	1	1	0	1
Somnolence	1	0	1	2	0	2
Swelling	1	0	1	1	0	1
Vision blurred	1	0	1	1	0	1

All adverse events possibly, probably, or definitively related to the study medication. Adverse events (AE) classification was done according to MedDRA® 25.1. Reported are unique preferred terms per subject (repeated occurences of the same AE within one participant are counted only once) and total counts (including duplicates).

eTable 22. Suicidal Ideations

	Suicidal thoughts		Suicide attempts		Self injury	
	LSD (*n*=27)	Placebo (*n*=26)	LSD (*n*=27)	Placebo (*n*=26)	LSD (*n*=27)	Placebo (*n*=26)
**At screening**						
any during lifetime	13	9	0	0	1	0
6 months prior to inclusion	9	0	0	0	0	0
**During Study**						
Baseline	1	0	0	0	0	0
Week 1 Day 1	0	0	0	0	0	0
Week 1 Day 5	0	0	0	0	0	0
Week 2 Day 12	0	0	0	0	0	0
Week 2 Day 8	0	0	0	0	0	0
Week 3 Day 15	0	0	0	0	0	0
Week 3 Day 19	0	0	0	0	0	0
Week 4 Day 22	0	0	0	0	0	0
Week 4 Day 26	0	0	0	0	0	0
Week 5 Day 29	0	0	0	0	0	0
Week 5 Day 33	0	0	0	0	0	0
Week 6 Day 36	0	0	0	0	0	0
Week 6 Day 40	0	0	0	0	0	0
Week 10 Follow-up	1	0	0	0	0	0

Suicidal tendencies assessed by Columbia-Suicide Severity Rating Scale, C-SSRS. All occurences of suicidal thoughts, suicide attempts and non suicidal self injury before and during the study are reported.

One person in the LSD group endorsed any suicidal ideation. They responded “Yes” to Wish to be dead at Baseline and at Visit 10. The severity was 1 and the thoughts were infrequent and easy to control. The person attributed these thoughts at Baseline to a negative experience as a young adult, and these thoughts at Week 10 were “triggered by conversations with an ex who she broke up with one week ago.” All C-SSRS-SLV responses from Day 1 through Week 6 were No. All other participants had no endorsed items on the C-SSRS for the duration of the study.

eTable 23. Electrocardiographic Parameters

	absolute	Δ to Screening	absolute	Δ to Screening
**Screening**	LSD (*n*=27)		Placebo (*n*=26)	
HR (bpm)	60±11		63±10	
QT (msec)	402±29		391±26	
QTc (msec)	399±13		395±15	
**2 h after first dose**	LSD (*n*=27)		Placebo (*n*=26)	
HR (bpm)	61±9	-1±10	61±6	2±8
QT (msec)	399±24	4±19	393±23	-2±20
QTc (msec)	399±18	0±16	395±16	1±13
**2 h after last dose**	LSD (*n*=23)		Placebo (*n*=23)	
HR (bpm)	63±10	-2±10	60±11	2±12
QT (msec)	392±27	7±23	400±25	-7±25
QTc (msec)	397±14	2±15	397±14	-1±10

Electrocardiographic parameters according to treatment and visit. HR, heart rate; bpm, beats per minute; QT, QT-interval; QTc; frequency-adjusted QT-interval. Absolute values and differences to screening are in mean ± standard deviation.

eFigure 9. Blood Pressure and Heart Rate During the First Dose Administration

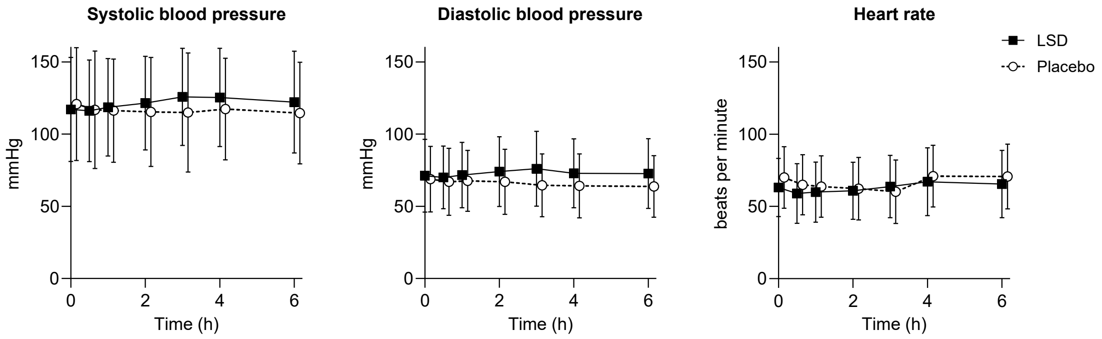

eFigure 9 Blood pressure and heart rate over 6 hours after the first dose administration. Data are mean ± SEM; *n* LSD = 27; *n* placebo = 26.

eTable 24. Laboratory Values at Different Visits

**Screening**	LSD				Placebo			
		absolute	abnormal			absolute	abnormal	
**Parameter (unit)**	*n*	mean ± SD	any	CS	*n*	mean ± SD	any	CS
**Hematology**								
Erythrocytes (10^12/L)	27	4.7 ± 0.4	4	0	26	4.7 ± 0.5	3	0
Hemoglobin (g/L)	27	142 ± 11	3	0	26	143 ± 14	2	0
Hematocrit (fraction of 1)	27	0.4 ± 0.03	2	0	26	0.4 ± 0.04	0	0
Ery. Mean Corpuscular Hemoglobin (pg)	24	31 ± 1.8	2	0	24	30 ± 1.6	1	0
Ery. Mean Corpuscular Volume (fL)	24	89 ± 4.5	1	0	24	88 ± 4.7	2	0
Leukocytes (10^9/L)	27	6.4 ± 1.9	3	0	26	6.9 ± 1.8	2	0
Platelets (10^9/L)	27	239 ± 56	0	0	26	230 ± 44	0	0
Neutrophils/Leukocytes (%)	24	61 ± 10.0	2	0	24	64 ± 5.8	0	0
Lymphocytes/Leukocytes (%)	24	29 ± 8.9	3	0	24	27 ± 4.8	2	0
Monocytes/Leukocytes (%)	24	6.5 ± 1.2	1	0	24	6.5 ± 1.4	1	0
Eosinophils/Leukocytes (%)	24	2.3 ± 1.7	2	0	24	1.7 ± 0.8	0	0
Basophils/Leukocytes (%)	24	0.6 ± 0.4	1	0	24	0.5 ± 0.2	0	0
**Chemistry**								
Sodium (mmol/L)	27	139 ± 1.9	2	0	26	138 ± 1.7	0	0
Potassium (mmol/L)	27	3.9 ± 0.2	0	0	26	3.9 ± 0.2	2	0
Chloride (mmol/L)	24	103 ± 1.9	0	0	24	103 ± 1.9	0	0
Calcium (mmol/L)	24	2.3 ± 0.07	1	0	24	2.3 ± 0.08	0	0
Phosphate (mmol/L)	24	1.0 ± 0.1	0	0	24	1.1 ± 0.2	1	0
Creatinine (umol/L)	27	76 ± 13	2	0	26	77 ± 12	1	0
Creatinine Clearance (mL/min/1.73 m2)	27	98 ± 13	0	0	26	105 ± 13	0	0
Urea Nitrogen (mmol/L)	27	4.2 ± 1.2	4	0	26	4.6 ± 1.2	1	0
Bilirubin (umol/L)	27	7.5 ± 4.2	0	0	26	8.4 ± 4.0	0	0
Direct Bilirubin (umol/L)	2	3.8 ± 1.8	1	0	1	4.3 ± 0.00	1	0
Alanine Aminotransferase (U/L)	27	22 ± 8.6	2	0	26	30 ± 25	3	0
Aspartate Aminotransferase (U/L)	26	24 ± 6.9	1	0	25	24 ± 8.8	3	0
Alkaline Phosphatase (U/L)	27	61 ± 17	2	0	26	60 ± 11	0	0
Gamma Glutamyl Transferase (U/L)	24	16 ± 7.7	2	0	24	21 ± 12	1	0
Albumin (g/L)	24	40 ± 3.9	1	0	24	41 ± 3.2	0	0
Lactate Dehydrogenase (U/L)	23	178 ± 24	3	0	24	171 ± 30	4	0
Protein (g/L)	24	72 ± 4.3	0	0	24	71 ± 4.6	1	0
Glucose (mmol/L)	27	5.1 ± 0.7	2	0	26	4.8 ± 0.5	0	0

Week 2, day 8	LSD					Placebo				
		absolute	Δ to screening	abnormal			absolute	Δ to screening	abnormal	
**Parameter (unit)**	*n*	mean ± SD	mean ± SD	any	CS	*n*	mean ± SD	mean ± SD	any	CS
**Hematology**										
Erythrocytes (10^12/L)	26	4.5 ± 0.5	-0.14 ± 0.3	8	0	26	4.7 ± 0.5	-0.07 ± 0.3	6	0
Hemoglobin (g/L)	26	138 ± 12	-3.84 ± 8.8	4	0	26	141 ± 14	-2.2 ± 7.9	4	0
Hematocrit (fraction of 1)	26	0.4 ± 0.04	-0.01 ± 0.03	3	0	26	0.4 ± 0.04	0.00 ± 0.02	2	0
Ery. Mean Corpuscular Hemoglobin (pg)	23	31 ± 1.6	0.05 ± 0.6	1	0	24	30 ± 1.8	-0.06 ± 0.7	1	0
Ery. Mean Corpuscular Volume (fL)	23	89 ± 4.9	0.2 ± 1.9	2	0	24	89 ± 5.1	0.5 ± 1.8	2	0
Leukocytes (10^9/L)	26	6.3 ± 2.0	-0.23 ± 1.9	1	0	26	6.0 ± 1.6	-0.9 ± 1.6	1	0
Platelets (10^9/L)	26	242 ± 46	1.4 ± 28	0	0	26	232 ± 47	2.8 ± 20	0	0
Neutrophils/Leukocytes (%)	23	60 ± 11	-0.9 ± 10	2	0	24	59 ± 7.4	-4.83 ± 7.6	0	0
Lymphocytes/Leukocytes (%)	23	29 ± 9.2	0.6 ± 8.0	3	0	24	30 ± 6.0	3.1 ± 5.9	0	0
Monocytes/Leukocytes (%)	23	6.5 ± 1.8	0.03 ± 1.8	1	0	24	9.5 ± 12	3.0 ± 12	1	0
Eosinophils/Leukocytes (%)	23	2.6 ± 2.0	0.3 ± 1.5	2	0	24	2.4 ± 1.7	0.8 ± 1.5	1	0
Basophils/Leukocytes (%)	23	0.5 ± 0.3	-0.06 ± 0.3	0	0	24	0.6 ± 0.3	0.08 ± 0.3	0	0
**Chemistry**										
Sodium (mmol/L)	26	138 ± 2.9	-0.88 ± 2.5	3	0	26	139 ± 1.7	0.2 ± 1.9	0	0
Potassium (mmol/L)	26	4.0 ± 0.2	0.06 ± 0.3	0	0	26	4.0 ± 0.3	0.07 ± 0.3	0	0
Chloride (mmol/L)	23	103 ± 2.6	0.3 ± 2.1	1	0	24	103 ± 1.6	0.7 ± 2.1	0	0
Calcium (mmol/L)	23	2.3 ± 0.06	-0.02 ± 0.08	0	0	24	2.3 ± 0.08	-0.01 ± 0.09	0	0
Phosphate (mmol/L)	23	1.0 ± 0.2	-0.03 ± 0.2	2	0	24	1.0 ± 0.1	-0.09 ± 0.2	0	0
Creatinine (umol/L)	26	74 ± 13	-1.38 ± 7.6	1	0	26	76 ± 13	-0.88 ± 5.5	1	0
Creatinine Clearance (mL/min/1.73 m2)	22	102 ± 14	1.5 ± 11	0	0	24	106 ± 14	0.7 ± 6.7	0	0
Urea Nitrogen (mmol/L)	26	4.4 ± 1.1	0.2 ± 1.3	3	0	26	4.7 ± 1.0	0.03 ± 0.9	0	0
Bilirubin (umol/L)	26	6.9 ± 4.3	-0.78 ± 3.7	0	0	26	7.8 ± 3.0	-0.53 ± 3.2	0	0
Direct Bilirubin (umol/L)	2	2.4 ± 0.4	-1.4 ± 2.1	0	0	1	3.1 ± 0.00	-1.2 ± 0.00	0	0
Alanine Aminotransferase (U/L)	26	22 ± 8.4	-0.62 ± 8.4	1	0	26	26 ± 19	-4.27 ± 19	1	1
Aspartate Aminotransferase (U/L)	25	22 ± 5.1	-2.21 ± 6.9	0	0	25	30 ± 39	6.5 ± 35	3	1
Alkaline Phosphatase (U/L)	26	60 ± 16	0.2 ± 5.8	3	0	26	58 ± 13	-1.58 ± 4.3	0	0
Gamma Glutamyl Transferase (U/L)	23	15 ± 7.4	-1.48 ± 2.1	1	0	24	19 ± 10	-2.71 ± 4.0	2	0
Albumin (g/L)	23	39 ± 3.1	-1.13 ± 2.9	1	0	24	39 ± 3.3	-1.37 ± 2.7	2	0
Lactate Dehydrogenase (U/L)	22	167 ± 31	-9.71 ± 30	2	0	23	171 ± 45	0.4 ± 43	3	1
Protein (g/L)	23	70 ± 4.1	-2.13 ± 3.8	2	0	24	70 ± 4.7	-1.21 ± 3.6	2	0
Glucose (mmol/L)	26	5.2 ± 0.9	0.1 ± 1.0	3	0	26	5.1 ± 0.8	0.3 ± 0.7	3	0

Week 6, day 36	LSD					Placebo				
		absolute	Δ to screening	abnormal			absolute	Δ to screening	abnormal	
**Parameter (unit)**	*n*	mean ± SD	mean ± SD	any	CS	*n*	mean ± SD	mean ± SD	any	CS
**Hematology**										
Erythrocytes (10^12/L)	22	4.6 ± 0.5	-0.07 ± 0.3	6	0	22	4.7 ± 0.4	0.01 ± 0.3	4	0
Hemoglobin (g/L)	22	140 ± 13	-2.08 ± 10	3	0	22	143 ± 14	0.3 ± 10	2	0
Hematocrit (fraction of 1)	22	0.4 ± 0.03	0.00 ± 0.03	1	0	22	0.4 ± 0.04	0.00 ± 0.03	0	0
Ery. Mean Corpuscular Hemoglobin (pg)	19	30 ± 1.8	0.02 ± 0.6	2	0	20	30 ± 1.9	-0.08 ± 0.8	2	0
Ery. Mean Corpuscular Volume (fL)	19	90 ± 5.9	0.8 ± 2.4	2	0	20	88 ± 5.4	-0.24 ± 1.9	2	0
Leukocytes (10^9/L)	22	6.4 ± 1.6	-0.31 ± 1.4	1	0	22	6.2 ± 2.1	-0.72 ± 1.3	3	0
Platelets (10^9/L)	22	231 ± 47	-10.09 ± 35	0	0	22	219 ± 43	-6.64 ± 27	0	0
Neutrophils/Leukocytes (%)	18	58 ± 6.8	-3.24 ± 8.8	0	0	20	58 ± 7.3	-5.93 ± 6.5	0	0
Lymphocytes/Leukocytes (%)	18	31 ± 7.3	3.2 ± 6.8	0	0	20	30 ± 6.9	3.9 ± 5.7	0	0
Monocytes/Leukocytes (%)	18	6.1 ± 0.8	-0.42 ± 1.1	0	0	20	7.3 ± 2.3	1.0 ± 2.3	1	0
Eosinophils/Leukocytes (%)	18	3.0 ± 1.5	0.4 ± 1.5	1	0	20	2.5 ± 1.5	0.8 ± 1.5	0	0
Basophils/Leukocytes (%)	18	0.5 ± 0.3	-0.08 ± 0.4	0	0	20	0.6 ± 0.3	0.08 ± 0.2	0	0
**Chemistry**										
Sodium (mmol/L)	22	139 ± 2.3	-0.18 ± 2.2	2	0	22	139 ± 2.3	0.5 ± 2.6	0	0
Potassium (mmol/L)	22	3.9 ± 0.2	-0.04 ± 0.3	2	0	22	4.1 ± 0.3	0.2 ± 0.3	1	0
Chloride (mmol/L)	19	104 ± 2.1	0.8 ± 2.0	0	0	20	104 ± 2.0	1.0 ± 2.2	0	0
Calcium (mmol/L)	19	2.3 ± 0.09	-0.04 ± 0.09	0	0	20	2.3 ± 0.06	-0.01 ± 0.1	0	0
Phosphate (mmol/L)	19	1.0 ± 0.1	-0.01 ± 0.1	0	0	20	1.1 ± 0.2	-0.05 ± 0.1	0	0
Creatinine (umol/L)	22	75 ± 13	-0.5 ± 6.2	1	0	22	76 ± 10	1.0 ± 7.6	0	0
Creatinine Clearance (mL/min/1.73 m2)	19	103 ± 12	1.0 ± 9.0	0	0	20	105 ± 13	-1.16 ± 9.1	0	0
Urea Nitrogen (mmol/L)	22	4.8 ± 1.4	0.5 ± 1.2	2	0	22	5.1 ± 1.2	0.4 ± 1.4	1	0
Bilirubin (umol/L)	22	7.6 ± 4.3	-0.1 ± 3.1	0	0	22	7.3 ± 2.5	-0.6 ± 3.8	0	0
Direct Bilirubin (umol/L)	2	3.5 ± 1.1	-0.35 ± 0.6	1	0	1	1.5 ± 0.00	-2.8 ± 0.00	0	0
Alanine Aminotransferase (U/L)	22	22 ± 7.3	-1.91 ± 7.2	0	0	22	21 ± 9.3	-5.32 ± 12	0	0
Aspartate Aminotransferase (U/L)	21	23 ± 4.3	-1.95 ± 7.5	0	0	22	21 ± 6.3	-1.76 ± 8.8	1	0
Alkaline Phosphatase (U/L)	22	63 ± 17	0.2 ± 3.8	2	0	22	60 ± 11	-0.05 ± 6.1	0	0
Gamma Glutamyl Transferase (U/L)	19	15 ± 5.6	-0.89 ± 3.1	0	0	20	17 ± 9.0	-3.45 ± 6.5	1	0
Albumin (g/L)	19	39 ± 2.8	-1.16 ± 2.7	0	0	20	41 ± 2.8	0.00 ± 2.9	0	0
Lactate Dehydrogenase (U/L)	18	169 ± 25	-10.65 ± 21	2	0	20	165 ± 32	-5.00 ± 23	5	0
Protein (g/L)	19	71 ± 4.1	-1.53 ± 4.0	0	0	20	72 ± 3.8	0.9 ± 4.1	0	0
Glucose (mmol/L)	22	5.3 ± 0.8	0.2 ± 1.0	3	0	22	5.2 ± 0.8	0.4 ± 0.8	3	0

Week 6, day 40	LSD					Placebo				
		absolute	Δ to screening	abnormal			absolute	Δ to screening	abnormal	
Parameter (unit)	*n*	mean ± SD	mean ± SD	any	CS	*n*	mean ± SD	mean ± SD	any	CS
**Hematology**										
Erythrocytes (10^12/L)	23	4.6 ± 0.5	-0.11 ± 0.3	5	0	23	4.7 ± 0.4	-0.02 ± 0.2	0	0
Hemoglobin (g/L)	23	140 ± 12	-2.31 ± 8.8	5	0	23	143 ± 14	-0.54 ± 7.5	1	0
Hematocrit (fraction of 1)	23	0.4 ± 0.03	-0.01 ± 0.02	1	0	23	0.4 ± 0.03	0.00 ± 0.02	1	0
Ery. Mean Corpuscular Hemoglobin (pg)	20	30 ± 1.7	0.2 ± 0.6	1	0	21	30 ± 1.8	-0.12 ± 0.7	1	0
Ery. Mean Corpuscular Volume (fL)	20	89 ± 4.8	0.3 ± 1.4	1	0	21	88 ± 5.2	-0.03 ± 1.9	1	0
Leukocytes (10^9/L)	23	6.6 ± 2.4	-0.18 ± 2.0	2	0	23	6.3 ± 1.8	-0.69 ± 1.7	1	0
Platelets (10^9/L)	23	231 ± 48	-13.26 ± 27	0	0	23	222 ± 48	-5.78 ± 26	0	0
Neutrophils/Leukocytes (%)	20	59 ± 8.0	-3.2 ± 9.9	0	0	21	59 ± 9.3	-4.31 ± 8.4	1	0
Lymphocytes/Leukocytes (%)	20	30 ± 6.9	2.6 ± 8.2	2	0	21	29 ± 7.3	2.5 ± 6.4	1	0
Monocytes/Leukocytes (%)	20	6.9 ± 1.7	0.4 ± 1.7	2	0	21	7.4 ± 3.2	1.1 ± 3.1	2	0
Eosinophils/Leukocytes (%)	20	2.6 ± 1.8	0.2 ± 1.7	2	0	21	2.4 ± 1.2	0.6 ± 1.2	0	0
Basophils/Leukocytes (%)	20	0.5 ± 0.3	-0.06 ± 0.3	0	0	21	0.5 ± 0.3	0.02 ± 0.3	0	0
**Chemistry**										
Sodium (mmol/L)	23	139 ± 2.0	0.04 ± 2.3	0	0	23	139 ± 2.4	0.3 ± 2.7	2	0
Potassium (mmol/L)	23	4.0 ± 0.2	0.05 ± 0.2	0	0	23	4.0 ± 0.3	0.1 ± 0.3	0	0
Chloride (mmol/L)	20	104 ± 2.0	0.7 ± 1.6	1	0	21	103 ± 1.9	0.4 ± 2.4	0	0
Calcium (mmol/L)	20	2.3 ± 0.09	-0.04 ± 0.07	0	0	21	2.3 ± 0.08	-0.02 ± 0.09	0	0
Phosphate (mmol/L)	20	1.0 ± 0.2	-0.02 ± 0.2	2	0	21	1.0 ± 0.1	-0.07 ± 0.2	2	0
Creatinine (umol/L)	23	73 ± 13	-2.52 ± 7.1	3	0	23	78 ± 12	2.1 ± 6.4	2	0
Creatinine Clearance (mL/min/1.73 m2)	20	103 ± 13	1.7 ± 10	0	0	21	104 ± 12	-2.21 ± 7.7	0	0
Urea Nitrogen (mmol/L)	23	4.6 ± 1.1	0.4 ± 0.8	1	0	23	4.9 ± 1.1	0.3 ± 1.2	0	0
Bilirubin (umol/L)	23	8.0 ± 4.9	0.4 ± 3.0	1	0	23	8.5 ± 4.0	0.5 ± 3.6	0	0
Direct Bilirubin (umol/L)	2	4.2 ± 1.3	0.3 ± 0.4	1	0	1	1.5 ± 0.00	-2.8 ± 0.00	0	0
Alanine Aminotransferase (U/L)	23	24 ± 8.8	0.00 ± 9.1	1	0	23	22 ± 12	-4.87 ± 11	1	0
Aspartate Aminotransferase (U/L)	21	23 ± 4.9	-2.5 ± 8.0	0	0	23	22 ± 6.2	-1.14 ± 7.6	1	0
Alkaline Phosphatase (U/L)	23	60 ± 15	-1.91 ± 5.5	2	0	23	58 ± 13	-1.3 ± 7.5	0	0
Gamma Glutamyl Transferase (U/L)	20	15 ± 6.6	-2.05 ± 3.1	1	0	21	17 ± 8.9	-3.67 ± 7.9	1	0
Albumin (g/L)	20	40 ± 3.7	-0.95 ± 2.8	1	0	21	40 ± 2.5	-0.43 ± 3.4	0	0
Lactate Dehydrogenase (U/L)	19	171 ± 27	-10.67 ± 26	1	0	21	168 ± 40	-3.00 ± 27	6	0
Protein (g/L)	20	70 ± 3.5	-2.5 ± 3.2	1	0	21	71 ± 3.3	0.05 ± 3.7	0	0
Glucose (mmol/L)	23	5.4 ± 0.8	0.3 ± 1.0	5	0	23	5.0 ± 0.7	0.1 ± 0.8	2	0

Week 10/End of study visit for dropouts	LSD					Placebo				
		absolute	Δ to screening	abnormal			absolute	Δ to screening	abnormal	
Parameter (unit)	*n*	mean ± SD	mean ± SD	any	CS	*n*	mean ± SD	mean ± SD	any	CS
**Hematology**										
Erythrocytes (10^12/L)	27	4.6 ± 0.5	-0.04 ± 0.3	8	0	26	4.7 ± 0.5	-0.02 ± 0.3	5	0
Hemoglobin (g/L)	27	140 ± 12	-1.88 ± 8.1	3	0	26	142 ± 16	-1.33 ± 8.6	5	0
Hematocrit (fraction of 1)	27	0.4 ± 0.03	0.00 ± 0.02	1	0	26	0.4 ± 0.04	0.00 ± 0.02	4	0
Ery. Mean Corpuscular Hemoglobin (pg)	24	30 ± 1.8	-0.11 ± 0.8	2	0	24	30 ± 1.7	-0.32 ± 0.6	1	0
Ery. Mean Corpuscular Volume (fL)	24	89 ± 4.7	-0.2 ± 1.4	2	0	24	88 ± 4.7	-0.43 ± 1.6	1	0
Leukocytes (10^9/L)	27	6.3 ± 1.9	-0.16 ± 1.8	2	0	26	6.4 ± 1.6	-0.51 ± 1.2	1	0
Platelets (10^9/L)	27	235 ± 52	-3.85 ± 46	1	0	26	227 ± 45	-2.96 ± 27	0	0
Neutrophils/Leukocytes (%)	24	59 ± 9.0	-1.22 ± 14	2	0	24	60 ± 7.7	-3.72 ± 7.9	0	0
Lymphocytes/Leukocytes (%)	24	29 ± 8.5	0.3 ± 12	2	0	24	29 ± 6.7	2.8 ± 6.2	0	0
Monocytes/Leukocytes (%)	24	7.0 ± 1.7	0.5 ± 1.9	4	0	24	6.5 ± 1.3	0.06 ± 1.6	1	0
Eosinophils/Leukocytes (%)	24	2.8 ± 1.5	0.5 ± 1.3	1	0	24	2.3 ± 1.1	0.6 ± 1.1	0	0
Basophils/Leukocytes (%)	24	0.5 ± 0.3	-0.05 ± 0.4	0	0	24	0.6 ± 0.3	0.07 ± 0.3	0	0
**Chemistry**										
Sodium (mmol/L)	27	140 ± 2.7	0.9 ± 2.9	1	0	26	139 ± 1.9	1.1 ± 1.9	0	0
Potassium (mmol/L)	27	3.9 ± 0.2	-0.03 ± 0.2	1	0	26	4.0 ± 0.3	0.06 ± 0.3	1	0
Chloride (mmol/L)	24	104 ± 3.0	1.0 ± 2.6	1	0	24	104 ± 2.1	1.5 ± 1.9	1	0
Calcium (mmol/L)	24	2.3 ± 0.08	-0.02 ± 0.08	0	0	24	2.3 ± 0.07	0.00 ± 0.09	0	0
Phosphate (mmol/L)	24	1.0 ± 0.1	-0.03 ± 0.1	2	0	24	1.1 ± 0.2	-0.03 ± 0.2	1	0
Creatinine (umol/L)	27	76 ± 13	0.07 ± 7.8	3	0	26	74 ± 14	-2.5 ± 7.0	0	0
Creatinine Clearance (mL/min/1.73 m2)	24	99 ± 13	-0.42 ± 11	0	0	24	108 ± 13	2.2 ± 8.1	0	0
Urea Nitrogen (mmol/L)	27	4.4 ± 1.1	0.1 ± 1.1	2	0	26	5.1 ± 1.0	0.5 ± 1.2	0	0
Bilirubin (umol/L)	27	8.2 ± 4.8	0.6 ± 3.9	1	0	26	7.4 ± 3.5	-0.96 ± 2.8	0	0
Direct Bilirubin (umol/L)	2	3.0 ± 0.1	-0.85 ± 1.9	0	0	1	4.4 ± 0.00	0.1 ± 0.00	1	0
Alanine Aminotransferase (U/L)	27	22 ± 7.8	0.1 ± 8.8	0	0	26	24 ± 13	-5.92 ± 19	1	0
Aspartate Aminotransferase (U/L)	27	23 ± 5.8	-1.00 ± 8.0	1	0	26	24 ± 7.8	0.2 ± 4.5	1	0
Alkaline Phosphatase (U/L)	27	62 ± 15	1.1 ± 9.0	2	0	26	60 ± 13	0.5 ± 5.6	0	0
Gamma Glutamyl Transferase (U/L)	24	16 ± 6.8	-0.42 ± 4.7	2	0	24	19 ± 11	-2.12 ± 5.0	2	0
Albumin (g/L)	24	39 ± 3.2	-1.29 ± 2.8	1	0	24	40 ± 2.5	-0.67 ± 2.7	0	0
Lactate Dehydrogenase (U/L)	24	170 ± 23	-7.00 ± 24	2	0	24	170 ± 33	-1.54 ± 33	5	0
Protein (g/L)	24	71 ± 3.3	-1.04 ± 4.5	1	0	24	71 ± 4.3	0.04 ± 3.3	1	0
Glucose (mmol/L)	27	5.1 ± 0.7	0.05 ± 0.9	5	0	26	5.2 ± 1.0	0.4 ± 1.2	5	0

Laboratory parameters at different visits. Mean, differences to screening and counts of values outside the normal range are shown. SD, standard deviation, CS, clinically significant.

eReferences.

1. Spencer TJ, Adler LA, Meihua Q, et al. Validation of the adult ADHD Investigator Symptom Rating Scale (AISRS). *J Atten Disord.* 2010;14(1):57-68.
2. Guy W. *ECDEU assessment manual for psychopharmacology.* Rockville, MD: US Deptment of Health, Education, and Welfare Publication (ADM); 1976.
3. Adler LA, Spencer T, Faraone SV, et al. Validity of pilot Adult ADHD Self- Report Scale (ASRS) to rate adult ADHD symptoms. *Ann Clin Psychiatry.* 2006;18(3):145-148.
4. Conners CK, Erhardt D, Sparrow MA. *Conners' Adult ADHD Rating Scale (CAARS).* New York, NY: Multihealth Systems Inc; 1999.
5. Posner K, Brown GK, Stanley B, et al. The Columbia-Suicide Severity Rating Scale: initial validity and internal consistency findings from three multisite studies with adolescents and adults. *Am J Psychiatry.* 2011;168(12):1266-1277.
6. Holze F, Ley L, Muller F, et al. Direct comparison of the acute effects of lysergic acid diethylamide and psilocybin in a double-blind placebo-controlled study in healthy subjects. *Neuropsychopharmacology.* 2022;47(6):1180-1187.
7. Holze F, Vizeli P, Ley L, et al. Acute dose-dependent effects of lysergic acid diethylamide in a double-blind placebo-controlled study in healthy subjects. *Neuropsychopharmacology.* 2021;46(3):537-544.
8. Ley L, Holze F, Arikci D, et al. Comparative acute effects of mescaline, lysergic acid diethylamide, and psilocybin in a randomized, double-blind, placebo-controlled cross-over study in healthy participants. *Neuropsychopharmacology.* 2023.
9. Dittrich A. The standardized psychometric assessment of altered states of consciousness (ASCs) in humans. *Pharmacopsychiatry.* 1998;31 (Suppl 2):80-84.
10. Studerus E, Gamma A, Vollenweider FX. Psychometric evaluation of the altered states of consciousness rating scale (OAV). *PLoS One.* 2010;5(8):e12412.
11. MacLean KA, Leoutsakos JM, Johnson MW, Griffiths RR. Factor analysis of the Mystical Experience Questionnaire: a study of experiences occasioned by the hallucinogen psilocybin. *J Sci Study Relig.* 2012;51(4):721-737.

12. Holze F, Duthaler U, Vizeli P, Muller F, Borgwardt S, Liechti ME. Pharmacokinetics and subjective effects of a novel oral LSD formulation in healthy subjects. *Br J Clin Pharmacol.* 2019;85:1474-1483.

13. Holze F, Liechti ME, Hutten N, et al. Pharmacokinetics and pharmacodynamics of lysergic acid diethylamide microdoses in healthy participants. *Clin Pharmacol Ther.* 2021;109(3):658-666.
