# Supplementary material for: Safety and Efficacy of Repeated Low-Dose LSD for ADHD Treatment in Adults: A Randomized Clinical Trial
Source: JAMA Psychiatry. 2025 Mar 19;82(6):555–62. doi: 10.1001/jamapsychiatry.2025.0044 (PMC11923771; doi:10.1001/jamapsychiatry.2025.0044)
Supplement: Supplement 3. — Data Sharing Statement [file jamapsychiatry-e250044-s003.pdf]

Data Sharing Statement

Mueller. Safety and Efficacy of Repeated Low-Dose LSD for ADHD Treatment in Adults. *JAMA Psychiatry*. Published March 19, 2025. doi:10.1001/jamapsychiatry.2025.0044

Data
Additional Information: ClinicalTrials.gov NCT05200936
Data available: No
